# Co-Designed Text Messaging Intervention for Parents of Children With Early Childhood Caries: Randomized Controlled Trial

**DOI:** 10.2196/91720

**Published:** 2026-11-03

**Authors:** Rebecca Chen, Michelle Irving, Harleen Kumar, Neeta Prabhu, Bradley Christian, Woosung Sohn, Heiko Spallek, Simone Marschner, Clara K Chow

**Affiliations:** 1 Population Oral Health, School of Dentistry, Faculty of Medicine and Health, The University of Sydney, Surry Hills, New South Wales, Australia; 2 Westmead Applied Research Centre, Faculty of Medicine and Health, The University of Sydney, Hawkesbury Road, Westmead, New South Wales, 2145, Australia, +61 28671444; 3 Paediatric Dentistry Department, Sydney Dental Hospital, Oral Health Services, Sydney Local Health District, Surry Hills, New South Wales, Australia; 4 School of Dentistry, Faculty of Medicine and Health, The University of Sydney, Surry Hills, New South Wales, Australia; 5 Paediatric Dentistry Department, Oral Health Services, Western Sydney Local Health District, Westmead, New South Wales, Australia; 6 Australian Centre for Integration of Oral Health, Australian Centre for Integration of Oral Health, School of Nursing & Midwifery, Western Sydney University, Liverpool, NSW, Australia

**Keywords:** mobile health, mHealth, dental caries, prevention, early childhood caries, co-design

## Abstract

**Background:**

Early childhood caries (ECC) is a prevalent yet preventable disease that significantly impacts young children’s health and quality of life. The World Health Organization’s *Global Strategy and Action Plan on Oral Health 2023–2030* highlights the potential of mobile health programs to enhance engagement and support sustained oral health behavior change.

**Objective:**

The aim of this study was to assess the effectiveness of the SmilesUp 12-week SMS text messaging–based education and support program to improve parents’ management of their children’s oral health behaviors compared to standard clinical care.

**Methods:**

We conducted a multisite, assessor-blinded, parallel-design randomized controlled trial among parents of children aged 0 to 6 years who had an ECC diagnosis and attended 1 of 6 public dental clinics in metropolitan, regional, and rural New South Wales, Australia. Participants were randomly assigned to either an intervention group receiving the fully automated 12-week SmilesUp program (3 SMS text messages weekly) or a control group. Both groups continued to receive usual standard dental clinical treatment. The primary behavioral outcome was parent-reported twice-daily toothbrushing with fluoride toothpaste. Secondary outcomes included adherence to oral health–promoting bedtime routines and child consumption of sugar-sweetened foods and beverages. Oral health self-efficacy was measured using 6 behavioral theory–mapped items across oral hygiene, diet, and bedtime routines.

**Results:**

A total of 154 participants were enrolled and randomized (n=78, 50.6% in the control group; n=76, 49.4% in the intervention group) across 6 sites, with 80.5% (124/154) completing the follow-up assessment. High program adherence was observed, with 96.1% (73/76) of intervention participants completing the program. At 12 weeks, a greater proportion of SmilesUp participants reported brushing their children’s teeth twice daily (45/61, 73.8% vs 42/63, 66.7%; adjusted odds ratio [aOR] 2.45, 95% CI 0.95-6.74; *P*=.07), though this difference was not statistically significant. Significant improvements were observed in adherence to bedtime routines (54/61, 88.5% vs 45/63, 71.4%; aOR 3.25, 95% CI 1.15-10.18; *P*=.03) as well as oral health knowledge on toothbrushing (46/50, 92.0% vs 41/52, 78.8%; aOR 5.47, 95% CI 1.48-25.19; *P*=.02). No statistically significant differences were found in sugar-sweetened food (10/61, 16.4% vs 4/63, 6.3%; aOR 3.72, 95% CI 1.02-17.14; *P*=.06) or beverage (25/61, 41.0% vs. 27/63, 42.9%; aOR 1.08, 95% CI 0.49-2.30; *P*=.85) consumption.

**Conclusions:**

These findings suggest that co-designed SMS text messaging–based programs show promise and may represent a feasible strategy for oral health behavior change. However, larger-scale and longer-term studies are needed to confirm sustained effectiveness using clinical outcomes. Future research should also explore ways mobile health can be scaled and cost-effectively embedded into comprehensive, interdisciplinary primary models of care.

## Introduction

Early childhood caries (ECC) affects more than 600 million children worldwide and remains one of the most common yet preventable childhood diseases [1]. Untreated ECC can lead to significant pain, infection, and impaired quality of life, often resulting in costly dental procedures, including treatments requiring general anesthesia (GA) [2]. In New South Wales (NSW), Australia, 1 in 250 children requires a GA admission for planned dental treatment, with 8.4% (1979/23,559) readmitted within 2 years and a further 9% (1641/18,194) of their siblings requiring similar care [3]. Similar rates in the United Kingdom also indicate that dental caries is the leading cause of hospital admissions for children aged 5 to 9 years (2015-2018) [4]. Preventive behaviors to reduce the risk of ECC include twice-daily toothbrushing with fluoride toothpaste, limiting sugar intake, and avoiding milk or sugar-sweetened beverages at bedtime [5]. While primary care dental clinics play a key role in diagnosis and clinical treatment, parents and caregivers are central to forming and maintaining the preventive behaviors that underpin children’s oral health [6]. The World Health Organization’s (WHO) 2019 *Ending Childhood Dental Caries* report emphasizes that parental knowledge, motivation, and capability—conceptualized as oral health self-efficacy—are essential in establishing oral health–promoting behaviors [6]. Systematic reviews [5] informing this WHO report and the 2023 update to Australia’s national oral health promotion messages [7] reinforce the preventive role that parents play. However, increasing parental knowledge alone is insufficient for sustained behavior change [8]. Traditional chairside advice and demonstration have limited effectiveness because behavior change requires repeated, practical, and accessible prompts that build parental oral health literacy and capability within the home environment [8,9].

Recognizing this gap, the WHO *Global Strategy and Action Plan on Oral Health 2023–2030* [10] advocates for the use of digital innovations, including mobile health (mHealth) [11]. mHealth interventions have shown to improve health behaviors for a broad range of cardiometabolic and chronic health conditions by increasing knowledge, capability, and motivation [12-14]. They are scalable, accessible, and effective across a range of noncommunicable diseases [12-14], yet their use in early childhood oral health remains emerging [15]. Existing programs for oral health largely target older adults and adolescents [16]. They narrowly focus on toothbrushing [17], are not of high quality [18,19], and are rarely grounded in behavioral theory [20,21].

To address these limitations, our research group undertook a comprehensive co-design process with parents to develop SmilesUp, a theory-informed mHealth intervention tailored to parental needs, preferences, and identified barriers and enablers [22]. The SmilesUp SMS text messaging program aims to enhance parental oral health self-efficacy by providing tailored information, including practical video demonstrations, and timely prompts delivered directly to parents within the home environment, where oral health habits are formed [22]. This randomized controlled trial (RCT) aimed to evaluate the impact of the SmilesUp SMS text messaging program on parental oral health behaviors, knowledge, oral health self-efficacy (capability, opportunity, and motivation), and overall engagement, addressing a critical gap in mHealth strategies to prevent ECC.

## Methods

### Study Design

This single-blinded parallel RCT (1:1 allocation ratio) was assessor blinded and carried out within NSW public dental clinics. The protocol has been previously published [23], and a concise summary of our methods is provided below. The trial adhered to the CONSORT-EHEALTH (Consolidated Standards of Reporting Trials of Electronic and Mobile Health Applications and Online Telehealth; Checklist 1) reporting guidelines.

### Participant Eligibility and Recruitment

Parents of children diagnosed with ECC who were either awaiting or undergoing dental treatment in the NSW public dental service in Australia were approached face-to-face for the study at 6 community dental health services, including 2 tertiary pediatric sites across metropolitan, rural, and regional areas. These areas collectively serve a population of approximately 4 million people and cover an area of 155,000 square kilometers. All clinics primarily offer a fee-free dental service for families unable to afford private care, with clinical and preventive care provided based on existing NSW public dental policies [24]. Prior to enrollment, all participants were given access to a participant information and consent form via eConsent through the REDCap platform (Multimedia Appendix 1). Parents of children who met all the following criteria were eligible to participate: (1) a clinical diagnosis of ECC, (2) age of 0 to 6 years inclusive, and (3) awaiting (or having attended) a public NSW dental service for dental caries treatment. Only 1 parent and 1 child per family were recruited. Parents were excluded if they (1) were already participating in an SMS text messaging–based study on lifestyle risk factors for health, (2) did not have proficient English-language skills to provide written informed consent, or (3) were unable to comply with study requirements. Parents of these children must also have basic computer and smartphone literacy and own an operational mobile phone capable of sending and receiving SMS text messages and accessing the internet.

### Procedures

Following completion of eConsent (Multimedia Appendix 1) through the REDCap web link provided via their smartphones, general demographic data, including the parent’s gender, ethnicity, country of birth, age category (18-20, 20-25, 26-35, and 36-45 years), and level of education, were collected via self-report. Information on the child’s age, number and order of siblings, Socio-Economic Indexes for Areas (SEIFA) rank [25,25] and whether the child had a planned dental procedure requiring GA was also collected from the parent. At the 12-week follow-up, data were collected through an online questionnaire available via the REDCap link that was provided as an SMS text message to the parent at the conclusion of the program. If the questionnaire was not completed, follow-up phone calls were made at the end of the 12 weeks.

### Randomization and Blinding

Participants were randomly assigned to either the intervention or control group at a 1:1 allocation ratio stratified by recruitment site (community dental clinics vs tertiary pediatric settings) and modality of care (dental procedures requiring GA vs dental procedures not requiring GA). Randomization was generated and accessed only by the study statistician using the *randomizeR* library in R (version 4.3.2; R Foundation for Statistical Computing) and was based on permuted block sizes (2,4,6) [26]. Clinicians recruited parent participants who self-enrolled in the study, and participants were randomly assigned to the study groups via computer generation and notified via SMS text message. All researchers were blinded as the TextCare system automatically implemented the SMS text messaging program in the intervention arm. Due to the nature of the intervention, participants were not blinded, but dental care providers and those analyzing the data were.

### Intervention

The SmilesUp intervention is a 12-week SMS text messaging program co-designed with parents and dental practitioners to target key ECC-related oral health behaviors, including twice-daily toothbrushing with fluoride toothpaste, bedtime routines, and consumption of sugar-sweetened foods and drinks [22]. The intervention aligns with WHO guidance to improve oral health through behavior change and is informed by the behavior change wheel (BCW) theory [27]. The BCW synthesizes 19 major behavior change theories to identify the capabilities, opportunities, and motivations required and the most appropriate intervention functions to support effective behavior change [27]. Messages aimed to improve oral health literacy and self-efficacy and provide brief reminders, reassurance, and links to smartphone-accessible video resources (Multimedia Appendix 2).

Participants directly received 36 semipersonalized messages (3 per week) to their mobile phones, delivered through the TextCare platform via an automated and preprogrammed schedule shaped by consumer engagement. Personalization included the children’s names and parents’ choice of morning or evening SMS text message delivery. The program was primarily one way but allowed for two-way communication, monitored asynchronously by a clinician researcher. Participants requiring clinical assistance received automated messages that contained the contact details for their nearest NSW public dental clinic or emergency services if the dental concern was urgent. Feedback on the program, including notifications on whether messages were received and read, was monitored asynchronously by a clinician researcher, with additional feedback collected at the 12-week follow-up.

The SmilesUp program was implemented alongside standard clinical dental care. The control group received standard care only, consisting of clinical assessment, treatment, and preventative advice provided by a dental practitioner. After completing the study period, parents in the control group were offered access to the SmilesUp intervention.

### Outcomes

The primary outcome was achieving the target behavior of brushing the children’s teeth twice a day with fluoride toothpaste. This item was analyzed dichotomously in response to an existing question used by NSW Health to evaluate other oral health programs: “How often do you brush your child’s teeth with toothpaste?” [28]. The primary outcome was achieved if participants responded “Twice a day” or “More than twice a day”; all other responses were classified as not meeting the outcome. Secondary outcomes were oral health–promoting behaviors, including use of fluoride toothpaste, reduced sugar-sweetened beverage consumption, reduced sugar-sweetened food consumption, bedtime routines related to the frequency and timing of milk consumption at night, and water consumption. Outcomes were ascertained through parental self-report using a standardized NSW Health questionnaire [28]. All definitions for primary and secondary behavioral outcomes are described in an a priori statistical analysis plan, which can be found in Multimedia Appendix 1.

Additional oral health self-efficacy outcomes were assessed using an adapted questionnaire based on the capability, opportunity, and motivation–behavior (COM-B) framework [29]. These questions were optional to reduce survey fatigue at enrollment. Existing oral health self-efficacy tools primarily focus on oral hygiene and are not typically grounded in behavioral theory [21]. In contrast, the questionnaire used in this study was aligned with the COM-B framework, which also underpinned the co-design of the SmilesUp intervention [22]. This approach was selected because it could be applied to a broad range of oral health–promoting behaviors addressed by the intervention (oral hygiene, diet, and bedtime routines). Accordingly, the questionnaire applied the COM-B framework to each behavior and addressed (1) physical capability, (2) psychological capability (ie, knowledge capability), (3) physical opportunity, (4) social opportunity, (5) reflective motivation, and (6) automatic motivation (Multimedia Appendix 1). Acceptance of the program was also assessed via the number of participants who completed the SmilesUp SMS text messaging intervention [23].

### Sample Size Calculation

A sample size of 150 participants provided 80% statistical power to detect a 25% increase in the target toothbrushing behavior in the intervention group from 53% to 78% compared to the control group at 12 weeks considering a 5% significance level and a 25% dropout rate. A cross-sectional study of a similar Australian population accessing public dental services found that 53% of children aged 3 to 8 years brushed their teeth twice a day with toothpaste [30].

### Statistical Analysis

Our statistical analysis followed an intention-to-treat principle and a prespecified statistical analysis plan (Multimedia Appendix 1). To assess the effectiveness of the intervention, a logistic regression model adjusting for whether parents performed toothbrushing for their children twice a day with toothpaste at baseline and the stratification variables was conducted, and adjusted odds ratios (aORs) with 95% CIs were reported. Similarly, all secondary behavioral outcomes were analyzed using the same logistic regression model. A statistical significance level of .05 was used. An interaction analysis of treatment and prespecified covariates (planned dental procedure requiring GA vs non-GA and tertiary vs nontertiary sites) was conducted to explore the heterogeneity of our results. No multiplicity adjustments were performed for secondary outcomes as they were exploratory by nature. If missingness was greater than 5%, missingness at random was assessed using 2-tailed *t* tests or chi-square tests for the relevant covariate. Additionally, a sensitivity analysis was conducted using multivariate imputation by chained equations with 50 imputations. No interim analyses were needed in alignment with stopping guidelines. All analyses were undertaken using R.

### Ethical Considerations

This study was conducted in accordance with the principles of the Declaration of Helsinki. The protocol was reviewed and approved by the Western Sydney Local Health District Human Research Ethics Committee (2022/ETH01920). All participants took part voluntarily without compensation and provided written informed consent prior to enrollment, with the ability to opt out at any time. To ensure privacy and confidentiality, all collected data were deidentified. Additionally, the trial was prospectively registered with the Australian New Zealand Clinical Trials Registry (ACTRN12623000325606).

## Results

### Study Participants

Of the 154 participants who were recruited and randomized in this study between August 24, 2023, and May 10, 2024, a total of 76 (49.4%) in the intervention group and 78 (50.6%) in the control group (n=124, 80.5%) completed follow-up data collection of primary and secondary behavioral outcomes at the end of the 12-week intervention (Figure 1). Additional outcomes focused on oral health literacy and self-efficacy were collected from 66.2% (n=102) of the participants at 12 weeks. Recruitment was completed once the anticipated sample size was reached.

Most parent participants (144/154, 93.5%) were above 25 years of age, 74.0% (114/154) were female, 47.4% (73/154) had completed a university degree, 57.1% (88/154) were born overseas, 18.8% (29/154) used general health apps, and 5.2% (8/154) used oral health apps (Table 1). The mean age of the children was 4.2 (SD 1.7) years, 31.8% (49/154) had a planned dental procedure requiring GA, 83.1% (128/154) had a sibling, 21.4% (33/154) had 4 or more siblings, 33.8% (52/154) had a younger sibling in the family, and 37.7% (58/154) were from lower–socioeconomic status areas as determined via postcodes (Socio-Economic Indexes for Areas 1-3; Table 2).

**Figure 1.**
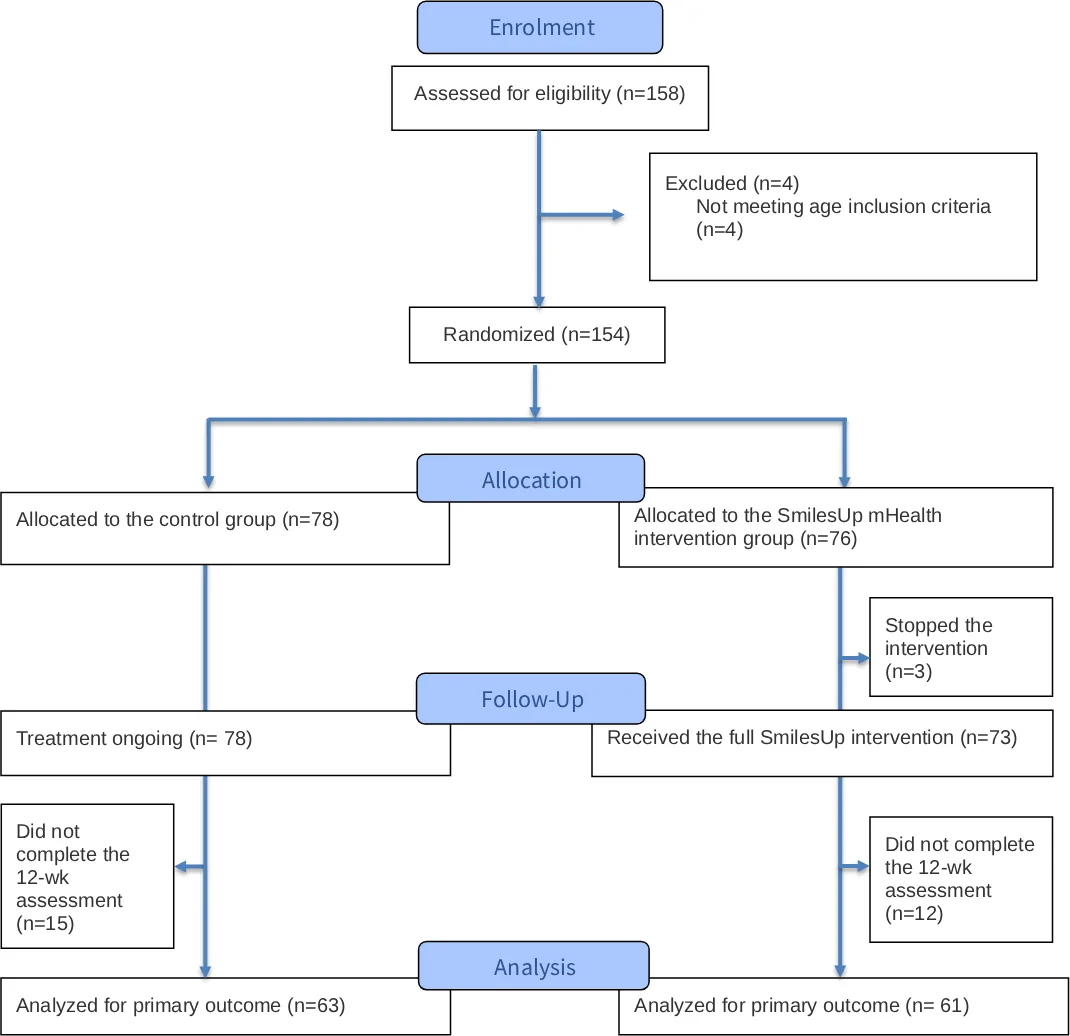
CONSORT (Consolidated Standards of Reporting Trials) diagram.

**Table 1.** Baseline demographic characteristics of parents by treatment group (N=154).

Characteristics	Control (n=78), n (%)	Intervention (n=76), n (%)	Overall, n (%)
Parent’s gender			
Men	20 (25.6)	20 (26.3)	40 (26.0)
Women	58 (74.4)	56 (73.7)	114 (74.0)
Parent’s age (y)			
18-25	3 (3.8)	3 (3.9)	6 (3.9)
26-35	31 (39.7)	25 (32.9)	56 (36.4)
36-45	35 (44.9)	43 (56.6)	78 (50.6)
≥46	6 (7.7)	4 (5.3)	10 (6.5)
Prefer not to answer	3 (3.8)	1 (1.3)	4 (2.6)
Parent’s level of education			
University degree	34 (43.6)	39 (51.3)	73 (47.4)
Nonuniversity degree	39 (50)	35 (46.1)	74 (48.1)
Prefer not to answer	5 (6.4)	2 (2.6)	7 (4.5)
Country of birth			
Australia	32 (41.0)	34 (44.7)	66 (42.9)
Overseas	46 (59.0)	41 (53.9)	88 (57.1)
Do you use oral health apps?	5 (6.4)	3 (3.9)	8 (5.2)
Do you use other health apps?	16 (20.5)	13 (17.1)	29 (18.8)

**Table 2.** Baseline demographic characteristics of the children by treatment group (N=154).

Characteristics	Control (n=78)	Intervention (n=76)	Overall
Child’s age (y), mean (SD)	4.3 (1.5)	4.1 (1.8)	4.2 (1.7)
Is your child receiving or have they received care with a general anesthetic?, n (%)	25 (32.1)	24 (31.6)	49 (31.8)
Total number of children in your family, n (%)			
1	13 (16.7)	13 (17.1)	26 (16.9)
2	36 (46.2)	29 (38.2)	65 (42.2)
3	11 (14.1)	19 (25)	30 (19.5)
4 or more	18 (23.1)	15 (19.7)	33 (21.4)
Do you have any other children who are younger than the child receiving dental treatment? (“yes” responses), n (%)^a^	25 (32.1)	27 (35.5)	52 (33.8)
Do you have any other children who are older than the child receiving dental treatment? (“yes” responses), n (%)	36 (46.2)	28 (36.8)	64 (41.6)
I have children that are both older and younger (“yes” responses), n (%)	9 (11.5)	9 (11.8)	18 (11.7)
Not applicable to me (I have one child) (“yes” responses), n (%)	13 (16.7)	16 (21.1)	29 (18.8)
SEIFA^b^ (IRSAD^c^ quintile), n (%)			
1 (most disadvantaged)	16 (20.5)	9 (11.8)	25 (16.2)
2	8 (10.3)	12 (15.8)	20 (13.0)
3	7 (9.0)	6 (7.9)	13 (8.4)
4	18 (23.1)	15 (19.7)	33 (21.4)
5 (most advantaged)	29 (37.2)	34 (44.7)	63 (40.9)

a Responses for the four questions on siblings do not sum to 100% because multiple responses were allowed.

b SEIFA: Socio-Economic Indexes for Areas.

c IRSAD: Index of Relative Socio-economic Advantage and Disadvantage.

### Primary Outcomes

Of the 76 parents who were randomized to the intervention group, 73 (96.1%) completed the 12-week SmilesUp program. At 12 weeks, 73.8% (45/61) of participants who received the SmilesUp SMS text messaging program reported brushing their children’s teeth twice a day compared to 66.7% (42/63) in the control group (aOR 2.45, 95% CI 0.95-6.74; *P*=.07; Table 3). The primary outcome was missing for 19.5% (30/154) and similar in both groups. Examination for bias indicated missingness to be at random (Multimedia Appendix 3). The sensitivity analysis using multiple imputation by chained equations analysis demonstrated that the study groups yielded similar results (aOR 1.93, 95% CI 0.75-4.96; *P*=.17) and was not statistically significant.

Our interaction analysis showed similar intervention effects among families who had or had not undergone a planned dental procedure requiring GA (*P*=.95) and whether families were recruited from tertiary or nontertiary settings (*P*=.12).

**Table 3.** Primary and secondary behavioral outcomes.

	Control, n (%)		Intervention, n (%)		aOR^a^ (95% CI)	*P* value^b^
	Baseline (n=78)	12 wk (n=63)	Baseline (n=76)	12 wk (n=61)		
Primary outcome						
Met recommended toothbrushing requirements	49 (62.8)	42 (66.7)	43 (56.6)	45 (73.8)	2.45 (0.95-6.74)	.07
Secondary outcomes						
Met recommended fluoride toothpaste use	68 (87.2)	62 (98.4)	62 (81.6)	57 (93.4)	0.27 (0.01-2.18)	.27
Met oral health–promoting bedtime routines	58 (74.4)	45 (71.4)	60 (78.9)	54 (88.5)	3.25 (1.15-10.18)	.03
Met recommended sugar-sweetened beverage consumption	27 (34.6)	27 (42.9)	20 (26.3)	25 (41.0)	1.08 (0.49-2.39)	.85
Met recommended sugar-sweetened food consumption	8 (10.3)	4 (6.3)	7 (9.2)	10 (16.4)	3.72 (1.02-17.14)	.06
Met recommended water consumption	74 (94.9)	57 (90.5)	73 (96.1)	60 (98.4)	6.53 (0.90-139.76)	.11

a aOR: adjusted odds ratio.

b *P* value using logistic regression adjusting for the corresponding outcome at baseline and stratification variables (general anesthesia [GA] vs non-GA and tertiary vs nontertiary sites).

### Secondary Outcomes

For secondary outcomes, intervention participants reported better oral health bedtime routines (54/61, 88.5% vs, 45/63, 71.4%; aOR 3.25, 95% CI 1.15-10.18; *P*=.03). Sugar-sweetened food consumption showed a consistent direction of effect, although it was not statistically significant (aOR 3.72, 95% CI 1.02-17.14; *P*=.06). There was no statistically significant difference between the groups for sugar-sweetened beverage consumption (aOR 1.08, 95% CI 0.49-2.39; *P*=.85), use of fluoride toothpaste (aOR 0.27, 95% CI 0.01-2.18; *P*=.27), or adequate water consumption (aOR 6.53, 95% CI 0.90-139.76; *P*=.11; Table 3).

### Oral Health Self-Efficacy Outcomes

Additional outcomes of oral health self-efficacy were reported in a subset of participants in the intervention group (50/102, 49.0%) and the control group (52/102, 51.0%). They were overall suggestive of a pattern of improved self-efficacy, although few measures met statistical significance. A greater percentage of intervention participants reported improved knowledge on how to brush their children’s teeth (aOR 5.47, 95% CI 1.48-25.19; *P*=.02) and how to maintain a healthy bedtime routine (aOR 5.76, 95% CI 1.38-32.16; *P*=.03). There was no difference in knowledge on providing healthy drink and snack options (aOR 2.7, 95% CI 0.60-14.97; *P*=.21) or automatic motivation regarding oral health–promoting bedtime routines (aOR 3.01, 95% CI 0.96-10.54; *P*=.07; Table 4).

**Table 4.** Oral health self-efficacy outcomes.

Statement (capability, opportunity, and motivation–behavior domain related to oral health self-efficacy)	...brush my child’s teeth (oral hygiene)		...provide healthy drink and snack options (diet)		...support oral health–promoting bedtime routines	
	aOR^a^ (95% CI)	*P* value^b^	aOR (95% CI)	*P* value	aOR (95% CI)	*P* value
I can physically... (physical capability)	1.13 (0.38-3.33)	.82	0.830 (0.17-3.91)	.81	5.19 (1.16-31.33)	.045
I have all the knowledge to... (psychological capability)	5.47 (1.48-25.19)	.02	2.7 (0.60-14.97)	.21	5.76 (1.38-32.16)	.03
I have all the things I need to... (physical opportunity)	0.98 (0.08-12.04)	.99	1.66 (0.36-8.32)	.51	4.42 (0.92-28.38)	.08
People around me think it is normal for me to... (social opportunity)	2.34 (0.55-11.78)	.26	1.56 (0.40-6.54)	.53	2.22 (0.64-8.53)	.22
I am motivated to... (reflective motivation)	0.87 (0.24-3.01)	.83	1.77 (0.15-22.59)	.64	2.03 (0.40-12.20)	.40
This is something I do automatically without thinking about whether to... (automatic motivation)	1.50 (0.60-3.84)	.39	2.43 (0.70-9.54)	.18	3.01 (0.96-10.54)	.07

a aOR: adjusted odds ratio.

b *P* value using logistic regression adjusting for the corresponding outcome at baseline and stratification variables (general anesthesia [GA] vs non-GA and tertiary vs nontertiary sites).

## Discussion

### Principal Findings

The SmilesUp trial is the first SMS text messaging randomized controlled clinical trial specific for ECC home management, using the BCW framework [27] tested in an Australian public dental setting. Most participants completed the SmilesUp program, and while the primary outcome (twice-daily brushing) was not statistically significantly different, some secondary outcomes suggested improvements in oral health behaviors and oral health self-efficacy. Together, these findings suggest that the program may have potential benfit if implemented at scale; however, larger and longer confirmatory trials are needed to determine effectiveness over time.

In our sample, 18.8% (29/154) of parents reported using health apps, but only 5.2% (8/154) used oral health apps. This highlights both a gap and an opportunity to design high-quality mHealth oral health programs that respond to growing demand for digital preventive health care [31-33]. No interaction effects were observed among families whose children had a planned dental procedure requiring GA and those children who received care in community dental clinics without a GA. These findings indicate an opportunity to test the effect for families using community dental clinics with and without a GA in a future full-scale RCT and ensure that the study is adequately powered to enable subgroup analyses. Additionally, the intervention was highly acceptable, with a 96.1% (73/76) completion rate, indicating strong feasibility for a future confirmatory trial. Given the growing consumer preference for and acceptance of digital health support, particularly in the post–COVID-19 context [3,34], value-based health care systems should prioritize the integration of effective prevention-focused digital tools into routine health care delivery [31,34].

### Comparison With Prior Studies

This trial extends existing evidence in the field by demonstrating that mHealth interventions showed a consistent direction of effect to influence behaviors alongside improved parental knowledge on ECC risk factors. Our findings on parental knowledge are consistent with those of the systematic review by Ajay et al [35] on mHealth interventions targeting ECC risk factors, which showed improvements in oral health knowledge only. Importantly, this study moves beyond knowledge outcomes by providing evidence that mHealth interventions have the potential to affect behavioral outcomes, including oral health–promoting bedtime routines. Existing peer-reviewed literature demonstrates that changes in these behavioral outcomes are associated with favorable ECC clinical indicators, including the decayed, missing, filled teeth index; caries incidence; and the need for repeat invasive dental treatment [1,5,6].

### Strengths

The strengths of this study include its randomized clinical trial design; its evaluation of a broad set of oral health–promoting behavioral outcomes; and its implementation across a diverse participant population in metropolitan, regional, and rural NSW. The findings demonstrate the feasibility of an SMS text messaging–based program irrespective of socioeconomic status, mobile phone type, and geographical location, as well as the low cost of implementation for health services [36]. Importantly, the intervention was co-designed with parents and pre-evaluated for acceptability and usefulness [22], which likely contributed to the high engagement and completion rates. Evaluating the intervention alongside standard clinical care demonstrated the added value of the intervention and highlights the importance of multilevel approaches to support parents in maintaining sustained preventative oral health–promoting behaviors [37].

### Limitations

This study has limitations, including a follow-up rate of 80.5% (124/154), although this was balanced between the intervention and control groups [38]. The use of self-reported outcomes may have introduced social desirability bias compared to the use of clinical measures such as plaque scores; however, the randomized design ensured that this was balanced between the groups. The proportion of families reporting the primary outcome (twice-daily toothbrushing) at baseline was higher than anticipated (92/154, 59.7% compared to the anticipated 53%). Despite achieving close to the anticipated target of 78%, the study had limited ability to detect a statistically significant difference. This smaller-than-expected difference reduced the contrast between groups and, consequently, diminished the detectable effect size. Given the suggestions of benefit and low cost of the intervention, larger RCTs powered to detect smaller effect sizes would be justifiable. Recruitment within public dental clinics introduced selection bias; however, the inclusion of participants from diverse settings in metropolitan, regional, and rural areas may also support representativeness for the health system. The high level of missingness could have influenced the results; however, this was accounted for as participant characteristics were similar between groups (Multimedia Appendix 3). In fact, the high completion rate of participants previously not engaged in the target behavior of toothbrushing suggests that the intervention is particularly relevant for those most in need, highlighting potential benefits in health equity (Multimedia Appendix 3). The short follow-up period (12 weeks) limits conclusions regarding long-term behavioral maintenance and related clinical outcomes, highlighting the need for a full-scale RCT [38]. These future studies should explore whether mHealth initiatives such as SmilesUp can be effective in preventing longer-term clinical and service use related to ECC. Recruiting from a larger sample may also enhance the generalizability of these findings.

### Future Directions

Clinically, the results of the SmilesUp trial indicate that digital interventions have the potential to improve oral health behaviors and parental self-efficacy, which may translate into reduced ECC outcomes. Compared to traditional chairside advice, mHealth offers a less resource intensive approach that directly supports families in their home settings. The limited effect on some behaviors, including sugar consumption, also highlights the need for multipronged and interdisciplinary collaborative mHealth approaches to address common risk factors [39,40]. From a policy perspective, future efforts should focus on integrating mHealth into value-based, interdisciplinary models of care that recognize preventative behavior change in home settings as a critical component of health care delivery, complementing clinical dental treatment to achieve long-term oral and general health [38].

A process evaluation is also required to inform implementation strategies for scale-up, including culturally appropriate adaptation of the programs and alignment with other interdisciplinary family-focused preventive mHealth programs delivered in primary care settings [41]. Interoperability with electronic oral health records could enhance uptake and data collection to allow health systems to prospectively and longitudinally measure the program’s long-term effects, including clinical dental caries measures (decayed, missing, and filled teeth index), service use, and hospital admissions related to ECC [33,38]. Leveraging technologies including AI may further support tailored content delivery [42] and continuous improvement within learning health systems [43]. Future research should examine the impact of scaling mHealth programs that leverage AI on sustained oral health behavior change, long-term effectiveness, and cost-effectiveness.

### Conclusions

The SmilesUp mHealth program signals benefits and the potential of mHealth interventions to enhance oral health behaviors and underscores the need for larger studies with longer follow-up to determine the sustainability of behavior change and its translation into clinical outcomes. Future research should also explore optimal strategies for embedding mHealth innovations within broader primary prevention and interdisciplinary primary care frameworks and assess long-term preventative effectiveness and cost-effectiveness when delivered at scale.

## Supplementary material

10.2196/91720 Multimedia Appendix 1 Trial protocol and statistical analysis plan including the participant information and consent form.

10.2196/91720 Multimedia Appendix 2 Sample messages from the SmilesUp program.

10.2196/91720 Multimedia Appendix 3 Missingness data.

10.2196/91720 Checklist 1 CONSORT-EHEALTH checklist (V 1.6.1).

## Abbreviations

aOR: adjusted odds ratio
BCW: behavior change wheel
COM-B: capability, opportunity, and motivation–behavior
CONSORT-EHEALTH: Consolidated Standards of Reporting Trials of Electronic and Mobile Health Applications and Online Telehealth
ECC: early childhood caries
GA: general anesthesia
mHealth: mobile health
NSW: New South Wales
RCT: randomized controlled trial
WHO: World Health Organization
