# Supplementary material for: Co-Designed Text Messaging Intervention for Parents of Children With Early Childhood Caries: Randomized Controlled Trial
Source: JMIR Mhealth Uhealth. 2026 Nov 3;14:e91720. doi: 10.2196/91720 (PMC13730512; doi:10.2196/91720)
Supplement: Supplementary file 1 [file mhealth-v14-e91720-s001.pdf]

THE UNIVERSITY OF SYDNEY

SMILESup: Supporting parents/carers of young children with tooth decay to use a **M**obile health **I**ntervention targeting oral health **L**ifestyle behaviours and **E**valuating opportunities to **S**cale-**up**: a randomised control trial

SMILESup

CONFIDENTIAL

This document is confidential and the property of WARC / Rebecca Chen
No part of it may be transmitted, reproduced, published, or used without prior written authorisation from the institution.

STATEMENT OF COMPLIANCE FOR NON-DRUG OR DEVICE CLINICAL TRIALS

This document is a protocol for a clinical research study. The study will be conducted in compliance with all stipulations of this protocol, the conditions of ethics committee approval, the NHMRC National Statement on Ethical Conduct in Human Research (as updated) and the Handbook for Good Clinical Research Practice (GCP). The Therapeutic Goods Act has adopted ICH Guideline for Good Clinical Practice

TABLE OF CONTENTS

1.GENERAL INFORMATION ***3***

2. SYNOPSIS ***3***

3. RATIONALE / BACKGROUND ***4***

3.1 Dental caries (tooth decay) prevalence, current care **5**

3.2 Mobile Health and Dental Caries (tooth decay) **5**

3.3 Oral health literacy to prevent dental caries (tooth decay) **5**

3.4 Co-designing a theory informed text message intervention for dental caries (tooth decay) prevention **5**

5. AIMS / OBJECTIVES / HYPOTHESES5. .... ***6***

5.1 Primary hypothesis **6**

5.2 Aims **6**

5.3 Objectives **6**

6. RESEARCH PLAN *6*
6.1 Study design 6
6.2 Selection and recruitment and consent of study participants 7
6.3 Randomisation and blinding processes 8
6.4 Participant Withdrawal 10
6.5 Participant reimbursement 11
6.6 Monitoring of the clinical trial 11
6.7 Continuation of the intervention 11
7. STUDY INTERVENTION *11*
8. EVALUATION and OUTCOMES *13*
8.1 Outcome Measures & data collection instruments 13
8.2 Population and sample size including power calculation 15
8.3 Statistical analyses 15
9. SAFETY CONSIDERATIONS *15*
10. ETHICAL CONSIDERATIONS *16*
10.1 Risks 16
10.2 Benefits 16
11. DATA MANAGEMENT *17*
12. TIMELINES / MILESTONES *18*
All phases of the study will be complete within 24 months. *18*
13. DISSEMINATION OF RESULTS / PUBLICATION POLICY *19*
14. REFERENCES *19*

1.**GENERAL INFORMATION**

**Protocol title**	**SMILESup: S**upporting parents/carers of young children with tooth decay to use a **M**obile health **I**ntervention targeting oral health **L**ifestyle behaviours and **E**valuating opportunities to **S**cale-**up**: a randomised control trial
**Sponsor**	WSLHD
**Funder**	WSLHD
**Principal Investigators**	Professor Clara Chow – Director Westmead Applied Research Centre, The University of Sydney and WSLHD
**Contact**	Rebecca Chen (The University of Sydney) Rebecca.chen@sydney.edu.au
**Trial registration**	To be placed on the Australian New Zealand Clinical Trials Registry (ANZCTR) http://www.anzctr.org.au/ Currently under ethics review at WSLHD.

2. SYNOPSIS

**TITLE**	**SMILESup: S**upporting parents/carers of young children with tooth decay to use a **M**obile health **I**ntervention targeting oral health **L**ifestyle behaviours and **E**valuating opportunities to **S**cale-**up**: a randomised control trial
**CLINICAL PHASE**	PHASE 2 Randomised control trial (RCT)
**OBJECTIVES**	PHASE 2<br>To determine in a single-blinded RCT whether a 3-month text-messaging program will improve self-reported oral health behaviours (tooth brushing) of parents/carers with children at high risk of dental caries (tooth decay), compared to usual care.
**PRIMARY HYPOTHESIS**	Co-designed text message interventions will increase oral health behaviours more effectively than current practice.
**DESIGN**	Phase 2 is a single-blinded, two-arm parallel, RCT of 150 parents/carers of children with dental caries (tooth decay).
**BLINDING/MASKING**	Phase 2 is a single-blinded RCT. Researchers analysing the data will be blinded to treatment allocation. Participants and researchers involved in recruitment will not be blinded to allocation.
**OUTCOMES**	*Primary outcome*<br>PHASE 2

	Self-reported tooth brushing behaviours (yes or no to brushing their child's teeth twice a day) at three months.<br>*Secondary outcome*<br>PHASE 2<br>Self-reported oral health behaviours, this includes dietary related behaviours number of sweet snacks, number of sweet drinks and water consumption at three months.<br><br>Self-reported oral health self-efficacy measured using a questionnaire adapted from the Capability, Opportunity, and Motivation (COM) from the BCW[1] theoretical framework at three months.
**STUDY DURATION**	All phases of the study will be completed in 24 months.
**INTERVENTION/S**	A co-designed 3-month text messaging program to support parents/carers of children with dental caries.
**NUMBER OF PARTICIPANTS**	PHASE 2 (n=150) randomised 1:1 to either the intervention arm (text messaging program) or the usual care arm.
**POPULATION**	PHASE 2 – Parents/carers of children with dental caries. We will recruit from a NSW public health hospitals and clinics parents/carers of children waiting for a dental general anaesthetic hospital appointment. Sample size of (n=150), female and male, between the ages of 18-40.
**SELECTION AND ENROLMENT**	PHASE 2<br>Inclusion criteria are parents/carers of children who have all the following:<br>I. diagnosis of dental caries (tooth decay)<br>II. age 0 to 6<br><br>Parents/carers of these children must also own an operational mobile phone, capable of sending and receiving SMS text messages; and provide written informed consent.<br><br>Parents/carers will be excluded if they:<br>I. are already participating in a text message-based study on lifestyle risk factors for health.<br>II. have insufficient English skills to provide informed consent or read and understand text messages; and<br>III. are unable to comply with study requirements.

3. RATIONALE / BACKGROUND

3.1 Dental caries (tooth decay) prevalence, current care

In Australia 55% of children between the ages of 0-6, have Early Childhood Caries (ECC)[2]. Early Childhood dental caries (tooth decay) is a preventable non-communicable chronic disease with a complex aetiology that is closely associated with lifestyle related health behaviours of the parents/carers within family structures[3,4]. Although preventable for children, in the 0 to 6 age group our research team found that in NSW currently, one in every 250 children require hospitalisation each year, with a 9% repeat admission rate within a two-year period[5].

The key dental caries (tooth decay) risk factors are known[4], and oral health information to prevent tooth decay is usually provided to parents/carers at the clinical appointment. Standard treatment involves the provision of oral health education at the clinical appointment and the provision of a health promotion pamphlet to take home. The text messaging intervention seeks to innovatively provide these oral health messages in a timely piecemeal way (4 times per week). This will be an adjunct to standard treatment. This protocol outlines a set of research activities that will allows us to find out if providing prevention focused oral health information through text messages will influence oral health behaviour change in parents/carers with children who have dental caries more than usual care.

3.2 Mobile Health and Dental Caries (tooth decay)

Mhealth is the use of mobile technologies, such as mobile phones and applications, to support healthcare delivery[6]. There is growing evidence that mhealth interventions are effective in lifestyle and behavioural risk factor modification[7-9]. The largest evidence base is in text-messaging based programs that have been shown in various studies to offer a simple and proven way to provide support to people with chronic disease[10,11]. However, there is less application of mHealth interventions in oral health, and specifically little evidence for acceptability, effectiveness on behaviour change, for dental caries (tooth decay) prevention[12].

3.3 Oral health literacy to prevent dental caries (tooth decay)

WHO's definition on health literacy represents the cognitive and social skills which determine the motivation and ability of individuals to gain access to, understand and use information in ways which promote and maintain good health[13,14]. When applied to preventing tooth decay this definition moves beyond providing a pamphlet. Oral health literacy should include elements of motivation and empowerment related to the skills (e.g., tooth brushing) and self-efficacy to maintain sustained preventive behaviours.

3.4 Co-designing a theory informed text message intervention for dental caries (tooth decay) prevention

Co-design is the meaningful end-user engagement in research design and includes instances of engagement from a diverse range of relevant stakeholders that occur across all stages of the research process[15]. Health services are emphasising the need to involve consumers prior, during and in the development of health programs[16]. The WHO's implementation guide *Mobile technologies for Oral Health*[14], highlights the need to engage key implementation stakeholders and multiple end

users during the design and implementation of mobile oral health programs[14]. Although this guideline provides a library of messages, it emphasises the need to adapt these messages for the local context. It also emphasises the need to ground the program content in behaviour change theory. The Behaviour Change Wheel theory[1] has been suggested by the WHO and has been utilised in the rigorous design of other successful mHealth programs in Australia[8,17].

Our study seeks to implement principles of consumer engagement and the use of Behaviour Change theory[1] to firstly design an mHealth intervention specific to the context of our target population (parents/carers with children at high risk of dental caries). We will then assess the acceptability of the program content, before testing the effectiveness of the co-designed text message program to support oral health behaviours and improve the oral health literacy and self-efficacy of parents with children who have dental caries (tooth decay). We will conclude with a process evaluation to understand the barries, enablers to inform program improvements and opportunities to scale the program.

5. AIMS / OBJECTIVES / HYPOTHESES5.

5.1 Primary hypothesis

Co-designed text message intervention in addition to usual care will change oral health behaviours (tooth brushing) more effectively than current practice.

5.2 Aims

This randomised control trial, aims to co-design, assess the effectiveness, and evaluate a text message program to support oral health behaviours, self-efficacy and increase the oral health literacy of parents, compared to usual care.

5.3 Objectives

PHASE 2

Determine the effectiveness of the text message intervention in a single-blinded randomised control trial (RCT) on oral health behaviours (tooth brushing) of parents compared to those in usual care.

6. RESEARCH PLAN

6.1 Study design

PHASE 2 (RCT)

Single-blinded RCT to test the effectiveness of the text message intervention on the parental/carer self-reported oral health behaviours (e.g., toothbrushing), and oral health self-efficacy compared to usual care. Participants and researchers involved in recruitment will not be blinded to allocation. Only the researchers analysing the data will be blinded to treatment allocation.

6.2 Selection and recruitment and consent of study participants

The selection and recruitment strategy for each of the phases is outlined in table 1 below. Although all participants must fulfill the inclusion criteria, we do not expect the same participants to participate in all three phases of the study.

The participant information sheet and a participant consent form will be different for the different phases of the study and are outlined in the supplementary material to this protocol. All participants will be given an participant information sheet and a participant consent form. They will be required to provide informed consent through the return of consent form electronically, or via paper.

Table 1 Selection and recruitment

Selection	Recruitment & Consent [PICF that corresponds to this section]
PHASE 2 Randomised Clinical Trial (n=150)	
Inclusion criteria are parents/carers of children who have all the following:<br>I. diagnosis of dental caries (tooth decay)<br>II. age 0 to 6<br><br>Parents/carers of these children must also own an operational mobile phone, capable of sending and receiving SMS text messages; and provide written informed consent.<br><br>Parents/carers will be excluded if they:<br>I. are already participating in a text message-based study on lifestyle risk factors for health.<br>II. have insufficient English skills to provide informed consent or read and understand text messages; and<br>III. are unable to comply with study requirements.	Dental Practitioners at the WSLHD and SLHD paediatric dental departments will approach the parents/carers of patients attending their dental clinics. Clinics targeted will be the pre- general anaesthetic (pre-GA) assessment clinics prior to a GA procedure for dental caries. Dental practitioners will screen parents/carers for eligibility.<br>Only parents/carers of children who fit the eligibility criteria will be invited to take part in the study. These parents/carers will be provided with a recruitment information flyer (attached separately to this protocol), by the dental practitioner or delegated clinician/researcher.<br><br>In addition, we will reach out to other NSW Health public dental clinics listed in the recruitment sites and advertise the study to these communities.<br><br>The recruitment information flyer will contain a QR code to a REDCap link. This direct link will give parents the option to provide their details so that the research team can contact them or provide them the opportunity to complete the enrolment process at home in their own time.

	All the information about the study to enable informed consent and the PICF to enrol into the SMILESup text message program will be held on REDCap. Contact details of the research team will also be available in case any participant has further questions to enable informed consent.<br><br>After consenting, participants will be asked to complete the demographic questionnaire and a standardised oral health literacy questionnaire to measure the baseline oral health behaviours and self-efficacy (primary and secondary outcomes). Randomisation will also occur on completion of baseline data collection. Parents/carers in the intervention group will be sent a welcome text message that enrols them into the SMILESup text messaging program.<br>Parents/carers in the control group will be sent one text message with a link to oral health education information on the NSW Health website. At the end of the study period those in the control group have the option of receiving the text message program.<br><br>Where requested by a parent/carer a paper version of PICF and enrolment forms may be made available.<br><br>[MASTER PICF Phase 2 consumer intervention]

6.3 Randomisation and blinding processes

Participants will be randomly assigned to either the control or the intervention group with a 1:1 allocation ratio, stratified by recruitment site type (tertiary public setting vs. community dental clinics) and whether the patient will/was attending a General Anaesthetics (GA) appointment or non-GA . This will be managed by a computer-generated randomisation schedule. Participants will be notified of their treatment allocation via text message from the Textcare platform. Participants and researchers involved in recruitment will not be blinded to allocation. Only the researchers analysing the data will be blinded to treatment allocation (see figure 1 and Table 5).

6.3.1 Intervention group

Those who have been randomised into the intervention group will be sent text messages developed in phase one, for a period of three months. The text message program developed in phase 1 will be called SMILESup and is a three-month semi-personalised co-designed text messaging program. It is anticipated that there will be four messages/ per week for a period of three months. This is in alignment with suggestions in the WHO's Implementation Guidelines.

6.3.2 Control group

Those who have been randomly allocated to the control group will be sent a text message that links them to the NSW Health webpage for health promotion information. They will continue to receive one text message a month asking about their brushing habits. After the three-month RCT trial, they will have the option to be provided with the SMILESup text message program.

Figure 1 Study flow process

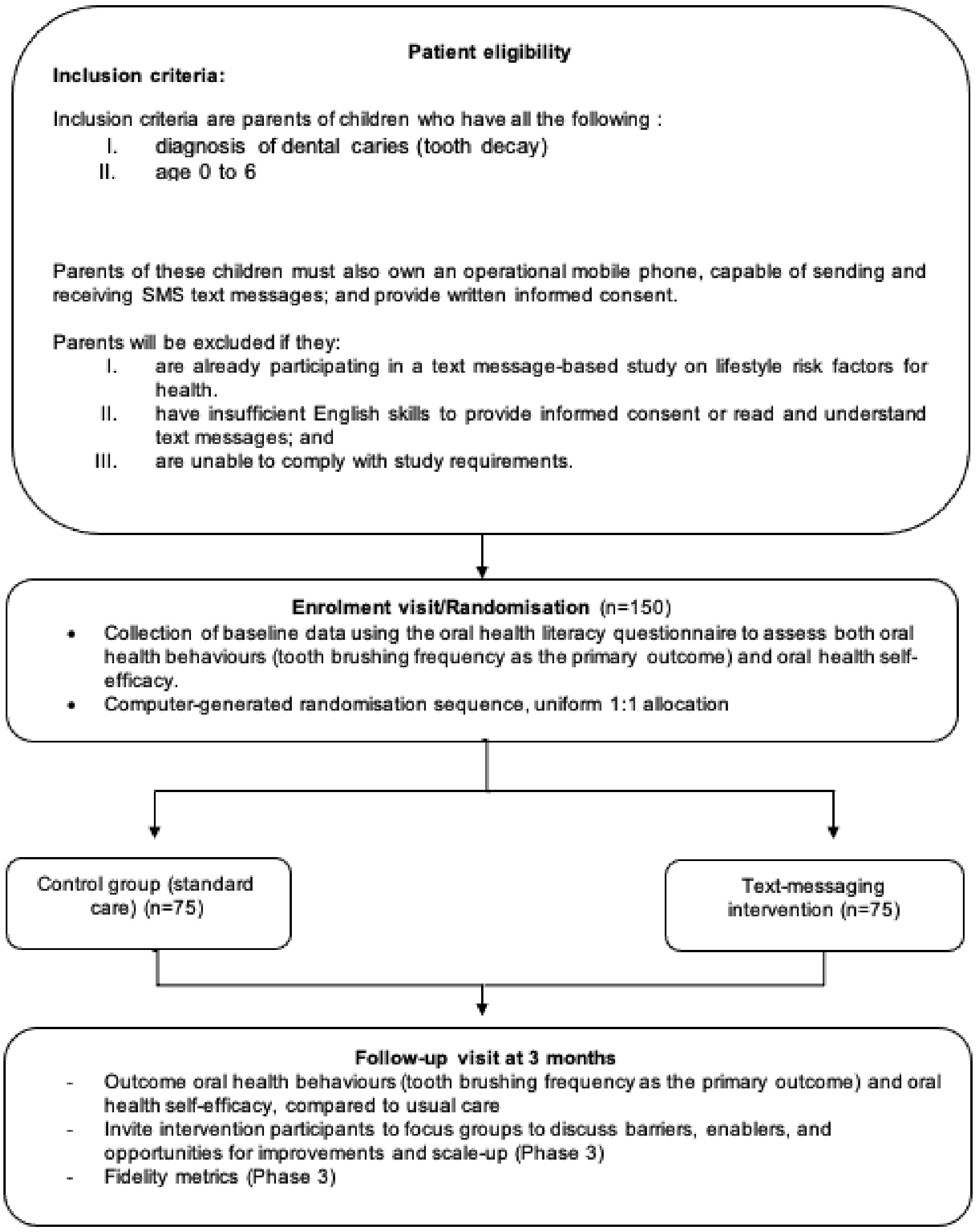

6.4 Participant Withdrawal

6.4.1 Reasons for withdrawal

Participants can withdraw from the study at any time in any of the phases without giving a reason, by informing researchers. Requests for study withdrawal can also be made at any time via phone call or email to the research team. For participants who will withdraw from the study, no further data will be collected. Withdrawal from the study will not affect the treatment or any follow-up care that participants receive, or their relationship with their dental practitioners, or study organisers.

For phase 2, participants can stop the intervention at any time by replying ‘STOP’ to any of the messages or contacting a member of the research team. The research team will stop the text messages within 72 hours.

6.4.2 Handling of withdrawals and losses to follow-up

Our power sample analysis allows for a 25% drop out rate. If a participant withdraws, we will not collect additional personal information from them, although information already collected will be retained to ensure that the results of the research project can be measured properly (through an intention to treat principle) and to comply with law. If participants stop the messages, they will still be able to complete the final questionnaire.

6.5 Participant reimbursement

Patients will only be reimbursement for associated travel costs (max. $20) if they choose to attend face to face workshops or face to face focus groups in phase 1 and 3 of the study.

6.6 Monitoring of the clinical trial

Monitoring process will be in alignment with the NSW Clinical trial monitoring framework. Participants will be informed that the intervention is a two-way text messaging program, meaning that they can reply to the messages. The Westmead Applied Research Centre (WARC) has a successful system Textcare (software program and a centrally located research assistant with a clinical background) who will monitor any replies for safety, according to the safety notification and reporting pathways for non-therapeutic goods trials.

If any participants require clinical assistance with their child's oral health during the study period, they will be provided contact details to their nearest NSW public dental clinic. An appointment may be provided according to the current eligibility policies in place within NSW public dental services. The details of a clinical contact person will also be provided on the PICF.

6.7 Continuation of the intervention

At the end of the three months clinical trial, upon completion of the close-out questionnaire, participants in the control group will be offered the option of receiving the text message program. All participants are free to save or share any of the messages they had received. Receiving this program will be completely optional for participants and free of charge.

7. STUDY INTERVENTION

The proposed intervention is a co-designed three-month long text message intervention.

The text messages will be developed using an established, co-designed process[18]. Figure 2 outlines the Modified WARC Co-design workshop process. The initiation meetings will confirm consensus on the themes of the text message content, which are anticipated to be related to the known key dental caries (tooth decay) risk factors.[4,19] Risk factors identified in the literature include: 1) oral hygiene, including age-appropriate use of fluorides, 2) diet, including increased consumption of water

and 3) night time rotuine, specific to the timing of diet and tooth brushing habits[4,19]. The modified WARC process will be implemented over 3 workshops to reduce participant burden. Each workshop meeting will last 1-1.5 hours, depending on whether data saturation of concepts is covered. It is ideal if workshop attendees are present at all three workshops.

Figure 2 Modified WARC co-design process

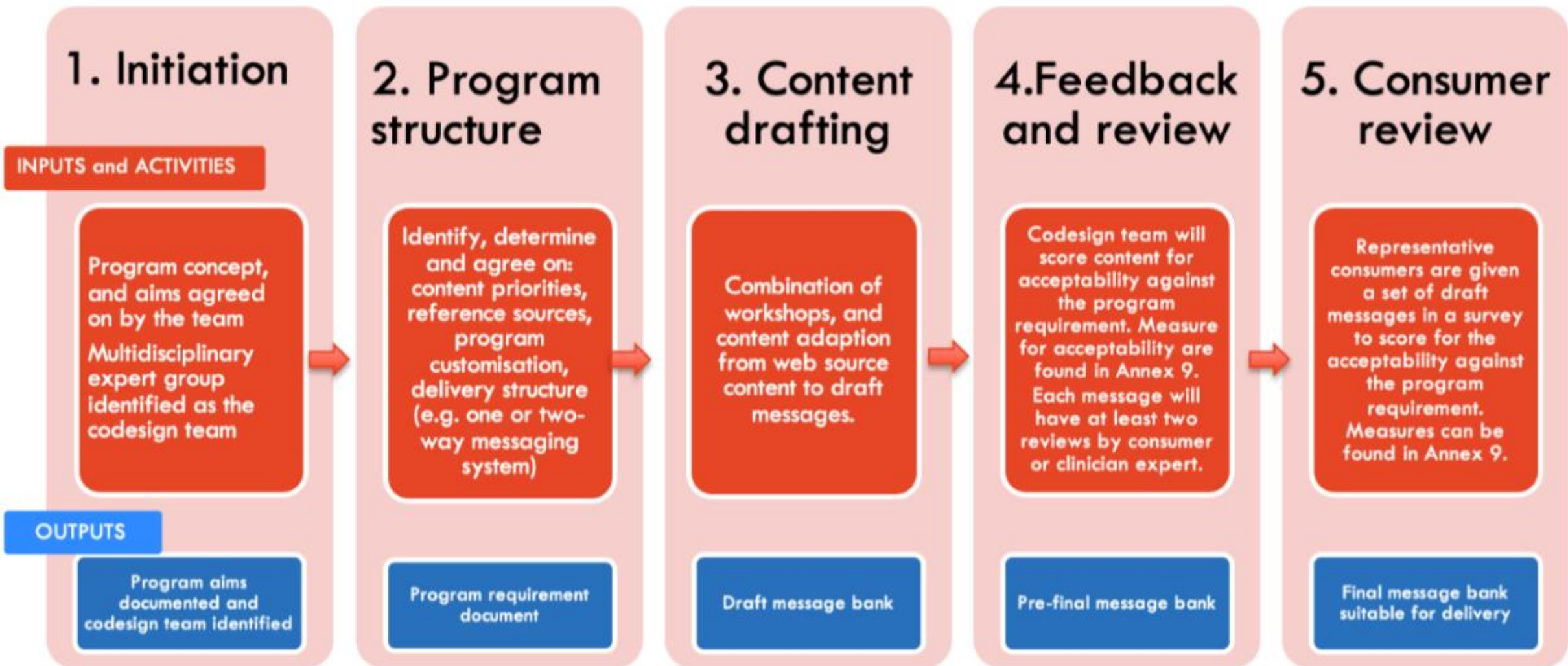

All messages that are included in the text message program for phase 2 are semi-personalised. This may mean that they will include the participant child's preferred name and specifications, such as the timing of when they would like to receive the messages. Examples of text messages are presented in Table 2. Each message will be reviewed on paper or on the Western Sydney Local Health District/ Office of Health Medical Research's Research Electronic Data Capture (REDCap) questionnaire by at least two health professionals and two consumers (parents/carers) for appropriateness, usefulness, and clarity on a 5-point Likert scale. All low-ranking text messages will be deleted from the final message bank. The study anticipates a one-way message delivery system, where messages will be sent four times per week from Monday to Saturday, at nominated time periods. Like other previously published text messaging studies[18], participants may, reply to some messages related to data collection as outlined in (table 5). They may also save, delete, or share messages.

Table 2. Examples of evidence-based text messages with different themes from or edited from the WHO's Be Healthy Be Mobile oral health literacy program[14] and previous studies including, Borelli (2019)[9] and the TEXT-ME study[18]

Category	Text message example
Oral Hygiene	Toothbrushing should start once your child starts getting their teeth.
Low-sugar diets	Feel like a snack for your child, <Insert name>? Fresh fruit, or veggie sticks are a healthy option for children.
Night-time diet and oral hygiene routine	Hi <Insert name>, even milk just before bedtime, without tooth brushing can increase the risk of tooth decay for your child.

8. EVALUATION and OUTCOMES

8.1 Outcome Measures & data collection instruments

All primary (table 3) and secondary outcomes (table 4) and associated data collection instruments are outlined below.

Standardised Oral health literacy questionnaire

WHO's definition on health literacy represents the cognitive and social skills which determine the motivation and ability of individuals to gain access to, understand and used information in ways which promote and maintain good health[13].

Without a validated Oral Health literacy measure suitable for the intervention a standardised self- reported Oral Health literacy measure was developed to measure key oral health behaviours . This questionnaire also measures self-efficacy mapped to the COM-B theoretical framework[1] . These questions are focused on three key dental caries oral health related behaviours: 1) oral hygiene 2) diet 3) night-time routine [3,4].

Question 1-15 will be asked at baseline and at three months. This questionnaire is attached separately to the protocol. Additionally, question 1 (primary outcome measure) will be asked via text message at the end of every month of all (control and intervention) participants in the program.

Table 3 Primary Outcomes

**Outcome**	**Instrument to measure outcome**
PHASE 2 Randomised control trial	
Self-reported tooth brushing behaviours. The primary outcome will be a binary (yes/no) response to the question "*How often do you brush your child's teeth with toothpaste?"* A yes response will be considered for patients answering, "Twice a day" or "More than twice a day" and a no response to those answering, "once a day", "few times a week", or "don't know /unsure".	How often do you brush your child's teeth with toothpaste?

Table 4 Secondary Outcomes

**Outcome**	**Instrument to measure outcome**
PHASE 2 Single-blinded randomised control trial	
Self-reported oral health behaviours, this includes dietary related behaviours number of sweet snacks, number of sweet drinks and water consumption three months.	Standardised oral health literacy questionnaire.

Self-reported oral health self-efficacy measured using a questionnaire adapted from the Capability, Opportunity, and Motivation (COM) from the BCW[1] theoretical framework at three months.	

<u>Table 5 Data collection time points.</u>

Example of recommended content for the schedule of assessments*	STUDY PERIOD					
	**Eligibility**	**Enrolment /Randomisation / Allocation**	**Post-allocation****			**Close-out**
**TIME POINT****	**$-t_1$**	**0**	**t_1**	**t_2**	**t_3**	**T_x**
**ENROLMENT:**						
**Eligibility screen**	✓					
**Informed consent**		✓				
**Randomisation / Allocation**		✓				
**INTERVENTIONS:**						
Text message program			✓	✓	✓	
Usual care			✓	✓	✓	
**ASSESSMENTS:**						
Primary outcome question –Tooth brushing question (single question)*		✓	✓	✓	✓	✓
**Standardised oral health literacy questionnaire (measures a range of secondary outcomes including oral health behaviours, and oral health self-efficacy)#**		✓				✓

*This question will be asked at baseline in the questionnaire and in the middle of every month via text message for all participants. That is, both the group that receives the text message program and those who receive usual care will be sent this text message.
This message will be:

"In the past week : How often did you brush your child's teeth with toothpaste? Text back the number according to your answer
1)Don't know /unsure
2)Few times a week
3)Once a day
4)Twice a day
5)More than twice a day"

Notes about the standardised oral health literacy questionnaire.
Questions 1-6 of the standardised questionnaire asks about oral health behaviours
Questions 7-15 of the standardised questionnaire asks about oral health self-efficacy, based on the BCW[1] theoretical model.
This standardised oral health literacy questionnaire is the secondary outcome and will be measured pre-and post-intervention only. All participants that is, both the

group that receives the text message program and those who receive usual care will be sent the final questionnaire.

** Post -allocation time points.
T1= the middle of the first month, T2 = the middle of the second month, T3= the middle of third month of the intervention.

8.2 Population and sample size including power calculation

The population this study is parents/carers of children accessing the NSW public dental services who have dental caries (tooth decay). Our study seeks to work with them in designing the intervention and seeks their feedback to improve the program and scale it for the future.

PHASE 2 Single-blinded RCT (n=150)
A sample size of approximately 150 will achieve a 80% power, to observe a meaningful difference of 25% increase of participants who brush twice a day in the intervention compared to the control group at three months (52% to 77%), using a 5% level of significance and accounting for a drop out of 25%. A cross-sectional study of a similar Australian population group reported 52% of children between 3-8 years old brushing twice a day[20].

8.3 Statistical analyses

PHASE 2 Single-blinded RCT (n=150)

The primary analysis will include all available participant data and will be performed at the end of the study after all data has been collected. The analysis of the primary and secondary outcomes will be according to the intention-to-treat principle. The primary outcome will be a binary (yes/no) response to the question "How often do you brush your child's teeth with toothpaste?" A yes response will be considered for patients answering, "Twice a day" or "More than twice a day" and a no response to those answering, "once a day", "few times a week", or "don't know /unsure". The primary analysis will be conducted using logistic regression. The odds ratio will be reported with a 95% confidence interval, a statistical significance level of 0.05 will be used. Secondary outcomes will be analysed using logistic regression for binary outcomes and linear regression analysis for continuous variables. Sub-group analyses may also be conducted comparing demographic variables, e.g., parental level of education (high vs. low) or families with single vs. multiple children. All analysis will be undertaken using R[23] or SPSS v22[24].

Full Statistical Analysis Plan in Appendix 1 at the end of the protocol document.

9. SAFETY CONSIDERATIONS

It is unlikely that there will be adverse effects from the text message intervention. Parent participants can opt out of the program at any time. Participant withdrawals will be removed from the text messaging platform.
If any participants require clinical assistance with their child's oral health during the study period, they will be provided contact details to their nearest NSW public dental clinic. The TextCare™ platform will be monitored by a clinician researcher. An appointment may be provided according to the current eligibility policies in place within NSW public dental services. The details of a clinical contact person will also be provided on the PICF.

10. ETHICAL CONSIDERATIONS

Ethics and governance approval is being sought with the WSLHD and SLHD.
The trial will also be prospectively registered on the ANZCTR.
All research activities are voluntary for participants, and written informed consent is provided to the research team prior to any data collection. Participant Information Statements (PIS) and Participant Consent forms (PCF) will be collected prior to any participation in the study.

10.1 Risks

<u>PHASE 2</u>
The risks associated with participating the SMILESup text messaging program as an adjunct and considered comparable to standard medical care. As outlined in the background of this protocol, the key caries risk factors are known. The intervention associated with this RCT will be monitored and participants are allowed withdraw at any time.

The participants understanding of the risks of the research in all phases is clearly outlined in the specific PICF statements before consent is given for their participation in the study.

10.2 Benefits

<u>PHASE 2</u>
For participants in phase 2 with direct benefits from the intervention there is potential to improve the oral health behaviours (tooth brushing habits) of parents/ carers on their children with dental caries (tooth decay). This will have a direct impact on the prevention of tooth decay for their child. The flow on effects may also include sustained behaviours change that influences other or subsequent children in the family.

If effective, future projects may scale the program to allow a larger number of parents/carers to improve tooth brushing habits for their children.

The participants expectations of the perceived benefits of the research will be clearly outlined in the specific PICF documents before consent is given for participation in the study. In accordance with the NHMRC guidelines all research activities will be conducted by trained researchers.

11. DATA MANAGEMENT

This section will provide more detail collection & usage, confidentiality, storage, record retention and archiving as part of the data management plan for this research study.

Collection & usage

According to the *NHMRC's Privacy Act Definition of Personal, Sensitive and Health Information*, data collected from this study is personal, sensitive, and considered health information. All data for all phases of the study will be collected from the individual themselves. No additional data will be gathered from medical/ health records. Thus, there are no conditions placed on our data to be shared with the data custodian. All usage of the data will only be used for the purpose of this study as outlined in the aims, objectives and statistical analysis outlined elsewhere in this protocol.

Confidentiality

All data analysed will be deidentified. Individual identity will be protected through the reporting of results in deidentified aggregate form. On enrolment and once initial data is collected, all participants will be assigned a study ID (e.g., '01') to ensure their confidentiality is protected. Thus, no personally identifiable information will be used during the analysis. Regarding the data collection from the online workshops, all original online recordings will be deleted once transcribed. Individuals can login anonymously. Online forums (zoom) will be password protected to ensure privacy of group. When results are discussed in public forums or published no identifiers will be used and individuals will not be able to be identified.

Storage

PHASE 2

An enrolment log will be maintained by a delegated member of the research team. This log will be stored on WSLHD servers. Access is maintained by the research team with the express permission of the Principal Investigator. Access to the WSLHD server is restricted by password and those with access must also be employees/contingency workers of WSLHD.

Data collected from the devices will be regularly uploaded to the iMSX Server which is linked to our cloud-based text messaging platform hosted in Australia, TextCare™. TextCare™ data is stored inside a Virtual Private Cloud in AWS commonly known as VPC, which is a logical partition of TextCare separating from other AWS accounts. This ensures that all unauthorised access will be prohibited. TextCare™ is further protected by network security, so that access to the database is directed via the application server. All other external connections to the database are restricted.

Textcare™ uses Amazon Relational Database Service (Amazon RDS); its system components are stored in encrypted instances using the industry standard AES-256 encryption algorithm to encrypt the data with a data key. Once encrypted, data can only be accessed by decrypting data with master keys, which are highly protected from unauthorised access.

Whilst most questionnaires and PICF forms are collected electronically, there may be a small number of participants requesting hard copies. Hard copies of consent forms and questionnaires will be stored in lockable filing cabinets in a restricted access room, only accessible by project staff restricted by swipe access provided by the University of Sydney. All study-specific confidential information will be destroyed after five years from the publication of the final report. Electronic data will be permanently deleted of servers and hard copy documentation shredded and disposed of in secure confidential bins provide by WSLHD.

All data for Phase 2 will also be entered into a REDCap (Research Electronic Data Capture) database under the Office for Health and Medical Research licence. The REDCap system is an online secure capture data collection and management tool for research use. It been provided by the Office for Health and Medical Research for the use of researchers in NSW Public Health LHDs, Networks, and pillars. REDCap is hosted in the eHealth AWS cloud, maintained by eHealth, supported by Office for Health and Medical Research. It is secured, and backed up daily, with privacy and confidentiality considerations are protected.

Study staff at each site will need to login into the REDCap database with a unique username and password. Once in the database staff will only have access to data collected at their specific site. Only coded data will be analysed. Only the chief investigator and principal investigator will have access to all study data.

Record retention and archiving process
Only the researchers named, or the study research assistant will have access to these data. Data will be held securely in a locked filing cabinet or on a password protected computer at Westmead Hospital. Following the NHMRC guidelines on management of data and information in research paper and electronic study information, including the consent forms and questionnaires, will be securely stored until 5 years after the completion of the study. The destruction of the data will follow processes following the University of Sydney Research Data Management processes.

12. TIMELINES / MILESTONES

All phases of the study will be complete within 24 months.

Table 6. Timeline of the study

Phase	Recruitment period	Data collection	Data analysis
Phase 2- Randomised Control trial	June 2023 – June 2024	June 2023 – October 2024	October November 2024

13. DISSEMINATION OF RESULTS / PUBLICATION POLICY

To ensure the translation of research, research findings will be disseminated in high impact oral health and medical internet research journals and discussed at scientific conference and other relevant public forums. No identifying information from participants will be discussed or published. All authors on the publications will abide by the ICMJE authorship guidelines

14. REFERENCES

1. Michie S, Richardson M, Johnston M, et al. The Behavior Change Technique Taxonomy (v1) of 93 Hierarchically Clustered Techniques: Building an International Consensus for the Reporting of Behavior Change Interventions. Annals of Behavioral Medicine. 2013;46(1):81-95.
2. Oral health and dental care in Australia [press release]. Australian Institute or Health and Welfare 2020. https://www.aihw.gov.au/reports/dental-oral-health/oral-health-and-dental-care-in-australia/contents/introduction. Accessed 2022
3. Seow Kim W. Environmental, maternal, and child factors which contribute to early childhood caries: a unifying conceptual model. International Journal of Paediatric Dentistry. 2012;22(3):157-168.
4. World Health Organisation. *Ending childhood dental caries: WHO implementation manual.* Geneva 2019.
5. Chen R, Schneuer FJ, Irving MJ, et al. Socio-demographic and familial factors associated with hospital admissions and repeat admission for dental caries in early childhood: A population-based study. *Community dentistry and oral epidemiology.* 2021.
6. Kay M, Santos J, Takane M. mHealth: New horizons for health through mobile technologies. *World Health Organization.* 2011;64(7):66-71.
7. WHO. *Mobile technologies for oral health: an implementation guide.* Geneva: WHO and International Telecommunication Union;2021.
8. Gould GS, Bar-Zeev Y, Bovill M, et al. Designing an implementation intervention with the Behaviour Change Wheel for health provider smoking cessation care for Australian Indigenous pregnant women.(Report). *Implementation Science.* 2017;12(1).
9. Borrelli B, Henshaw M, Endrighi R, et al. An Interactive Parent-Targeted Text Messaging Intervention to Improve Oral Health in Children Attending Urban

Pediatric Clinics: Feasibility Randomized Controlled Trial. *JMIR mHealth and uHealth.* 2019;7(11):e14247-e14247.

10. Singleton A, Raeside R, Partridge SR, et al. Co-designing a Lifestyle-Focused Text Message Intervention for Women After Breast Cancer Treatment: Mixed Methods Study. *Journal of medical Internet research.* 2021;23(6):e27076-e27076.
11. Bennell K, Nelligan RK, Schwartz S, et al. Behavior Change Text Messages for Home Exercise Adherence in Knee Osteoarthritis: Randomized Trial. *Journal of medical Internet research.* 2020;22(9):e21749-e21749.
12. Chen R, Santo K, Wong G, et al. Mobile Apps for Dental Caries Prevention: Systematic Search and Quality Evaluation. *JMIR mHealth and uHealth.* 2021;9(1):e19958-e19958.
13. WHO. Health literacy from the WHO's Health Promotion Glossary. 1998. https://www.who.int/healthpromotion/about/HPR%20Glossary%201998.pdf. Accessed 2022.
14. WHO. *Mobile technologies for oral health: an implementation guide.* Geneva: WHO and International Telecommunication Union; 2021.
15. Slattery P, Saeri AK, Bragge P. Research co-design in health: a rapid overview of reviews. *Health research policy and systems.* 2020;18(1):17-17.
16. Greenhalgh T, Hinton L, Finlay T, et al. Frameworks for supporting patient and public involvement in research: Systematic review and co-design pilot. *Health expectations : an international journal of public participation in health care and health policy.* 2019;22(4):785-801.
17. Nelligan RK, Hinman RS, Atkins L, Bennell KL. A Short Message Service Intervention to Support Adherence to Home-Based Strengthening Exercise for People With Knee Osteoarthritis: Intervention Design Applying the Behavior Change Wheel. *JMIR mHealth and uHealth.* 2019;7(10):e14619-e14619.
18. Redfern J, Thiagalingam A, Jan S, et al. Development of a set of mobile phone text messages designed for prevention of recurrent cardiovascular events. *European journal of preventive cardiology.* 2014;21(4):492-499.
19. Pitts NB, Baez R, DiazGuallory C. Early Childhood Caries: IAPD Bangkok Declaration. *International Journal of Paediatric Dentistry.* 2019;29(3):384-386.
20. Tadakamadla SK, Rathore V, Mitchell AE, Kaul A, Morawska A. Child- and family-level factors associated with toothbrushing frequency in a sample of Australian children. *International* journal of paediatric dentistry. 2022;32(5):639-648.
21. NVivo qualitative data analysis software; QSR International Pty Ltd. Version 12, 2018.
22. Braun V, Clarke V. Thematic analysis : a practical guide. London: SAGE Publications Ltd; 2022.

23. R Core Team (2022). R: A language and environment for statistical computing. R Foundation for Statistical Computing, Vienna, Austria. URL http://www.R-project.org/
24. IBM Corp. Released 2013. IBM SPSS Statistics for Windows, Version 22.0. Armonk, NY: IBM Corp.

Appendix 1 Full Statistical Analysis Plan
Appendix 2 Participant information and consent form

THE UNIVERSITY OF SYDNEY
Westmead Applied Research Centre

SMILESup:

Text messaging support for parents/carers of young children with tooth decay targeting oral health lifestyle behaviours

A multicentre, single-blinded randomised controlled trial to determine the comparative effectiveness of a customised mobile phone text messaging intervention (SMILES up) for parents/carers of young children with tooth decay for improving oral health lifestyle behaviours.

STATISTICAL ANALYSIS PLAN

Prepared by: *Simone Marschner/Rebecca Chen*

Study Statistician

Date: 12 September 2024

Signed by: Digital signature Clara Chow

Principal Investigator: *Prof. Clara Chow* (Rebecca Chen PhD student)
Date: 12/09/2024

Contents

1 TRIAL DESIGN 4
1.1 Primary objective 4
1.2 Secondary objectives 4
1.3 Intervention 5
1.4 Power and sample size 5
1.5 Schedule of Data Collection 6
2 PATIENT POPULATION 6
2.1 Inclusion criterion 6
2.2 Exclusion criterion 7
3 ANALYSIS 7
3.1 Analysis principles 7
3.2 Analysis methodology 7
3.3 Data inclusion definitions 7
3.4 Patient flow chart 8
3.5 Summary of patient disposition 9
3.6 Data completeness (for primary and secondary data) 9
3.7 Baseline characteristics 10
3.8 Primary outcome: 3-month analysis: 15
3.8.1 Primary outcome determination 15
3.8.2 Primary outcome analysis 15
3.8.3 Additional analysis related to the primary outcome at 1- 2 months 16
3.8.4 Missing data - sensitivity Analysis 16
3.8.5 Heterogeneity analysis 16
3.8.6 Subgroup analysis 17
3.9 Secondary behavioural outcomes at 3 months 17
3.9.1 behavioural outcome determination 17
3.9.2 Secondary outcome analysis 18
3.10 Secondary analysis of Oral Health Self-efficacy 19
3.10.1 Analysis of Oral Health Self-efficacy using the COM-B model 19
3.11 Safety Analysis 21

List of Tables

Table 1 Schedule of data collection ... 6
Table 2 Data collection windows for each outcome measure ... 7
Table 3 Summary of patient disposition ... 9
Table 4 Summary of data completeness ... 9
Table 5 Baseline demographic characteristics of parents by treatment group ... 10
Table 6 Baseline demographic of child by treatment group ... 11
Table 7 Baseline oral health behaviours by treatment group ... 12
Table 8 Baseline oral health self-efficacy by treatment group ... 13
Table 9 Primary outcome analysis of tooth brushing ... 15
Table 10 Descriptive Analysis of tooth brushing frequency ... 15
Table 11 Summary analysis of tooth brushing adherence ... 16
Table 12 Secondary outcome analysis for all binary secondary outcomes ... 18
Table 13 Descriptive analysis of secondary outcome behaviours that have frequency categories ... 18
Table 14 Descriptive analysis of toothpaste usage ... 19
Table 15 Analysis of oral health self-efficacy oral hygiene ... 20
Table 16 Analysis of each COM-B domain of the for Oral Health Self-Efficacy (OHSE) ... 21
Table 17 Descriptive analysis of mean for each COMB-B domain for oral health self-efficacy ... 21

List of Figures

Figure 1: Consort diagram for the study ... 8
Figure 2: Example of forest plot presentation of heterogeneity or interaction analysis ... 17

Abbreviations

RCT	Randomised Clinical Trial
ITT	Intent to treat
SD	Standard Deviation
IQR	Interquartile range
CI	Confidence Interval
n	Number in that cell
N_C	Number in the control group
N_T	Number in the treatment group

1 TRIAL DESIGN

This is a multisite, open label active control randomised controlled trial with a focus on feasibility was used to determine the comparative efficacy of a semi-personalised mobile phone text messaging intervention (SmilesUp) for parents/carers of a child at high risk of dental caries (tooth decay) compared to usual care.

1.1 Primary objective

To determine in a single-blinded RCT whether a 3month text-messaging program will improve self-reported oral health behaviours (tooth brushing) of parents/carers with children at high risk of dental caries (tooth decay), compared to usual care.

1.2 Secondary objectives

To determine in a single-blinded RCT whether a 3-month text-messaging program will improve self-reported oral health behaviours (toothpaste use, sugared sweetened beverage consumption, bedtime milk consumptions, sweet foods, water consumption) and oral health self-efficacy (capability-physical , capability-psychological, opportunity-physical, opportuntiy-social, motivationa-reflective, motivation-automatic) of parents/carers with children at high risk of dental caries (tooth decay), compared to usual care.

Each of the measures for oral health self-efficacy are as follows:

Oral Hygiene

1. Capability (physical): I physically can brush my child's teeth.
2. Capability (psychological): I have all the knowledge I need to brush my child's teeth with a toothbrush and toothpaste.
3. Opportunity (physical): I have all the things (toothbrush and toothpaste) to brush my child's teeth.
4. Opportunity (social): People around me (friends or family members, in person or online) think it is normal to brush my child's teeth.
5. Motivation (reflective): I am motivated to brush my child's teeth with a toothbrush and toothpaste.
6. Motivation (automatic): Brushing my child's teeth morning and night is something I automatically do without thinking about it.

Diet

7. Capability (physical): I physically can provide healthy food and drink options for my child.

8. Capability (psychological): I have all the knowledge I need to provide healthy food and drink options for my child.
9. Opportunity (physical): I have all the things (water bottle, access to shops with healthy food options) to provide healthy food and drink options for my child.
10. Opportunity (social): People around me (friends or family members, in person or online) think it is normal to choose healthy food and drink options for my child.
11. Motivation (reflective): I am motivated to provide healthy food and drink options for my child.
12. Motivation (automatic): Healthy food and drink options for my child are automatically given without me having to think about it.

Bedtime routine

13. Capability (physical): I physically can maintain a healthy bedtime routine.
14. Capability (psychological): I have the knowledge to maintain a healthy bedtime routine for my child.
15. Opportunity (physical): I have all the things to maintain a healthy bedtime routine for my child.
16. Opportunity (social): People around me (friends or family members, in person or online) think it is normal to maintain a healthy bedtime routine for my child.
17. Motivation (reflective): I am motivated to maintain a healthy bedtime routine for my child.
18. Motivation (automatic): A healthy bedtime routine is something I can provide my child without having to think about it.

1.3 Intervention

The intervention, *SmilesUp* is a 3-month co-designed semi-personalised text message program for parents/carers of young children (0 to 6 years old) to support oral health promoting behaviour change for their children. The program aimed to innovatively provide oral health messages addressing modifiable risk factors related to oral hygiene, diet, and bedtime routines. Parents choose when they received the messages, and some messages were personalised to their child.

1.4 Power and sample size

A sample size of approximately 150 will achieve an 80% power, to observe a meaningful difference of 25% increase of participants who brush twice a day in the intervention compared to the control group at three months (53% to 78%), using a 5% level of significance and accounting for a drop out of 25%. A recent cross-sectional study of a similar Australian population group from lower socioeconomic areas accessing public dental services reported 53% of children between 3-8 years old are brushing twice a day (Tadakamadla, Rathore, Mitchell, Kaul, & Morawska, 2022). However, 78% of children in Australia from high-income families brush twice a day (Do, 2016). Thus, to determine a meaningful difference we aimed to increase 25% of families brushing twice a day from 53% of the population who were brushing twice a day to be comparable to the of 78% of children from high-income households who were currently achieving the target behaviour.

Heterogeneity analyses will examine whether the treatment effect varies between levels of prespecified covariates on the primary efficacy variable of toothbrushing twice a day at 3 months. Additional subgroup analysis set a priori based on feasibility and implementation metrics and hypotheses will be performed for:

- Parents who had a child who received treatment via GA or non-GA.

- Parents who were in the "Steps to Smile program" (which is defined as parents of children patients who received a GA procedure and attended Murrumbidgee, Southern and Sydney LHD)

As this is an exploratory feasibility trial the sample size may be insufficient to determine significant outcomes for all secondary measures. This pilot study provides the feasibility data towards the design of future research that will measure long term dental caries outcomes and service utilisation patterns including the reduction of general anaesthetic admissions.

1.5 Schedule of Data Collection

Table 1 Schedule of data collection

	Baseline	4 Weeks Post-enrolment	8 Weeks Post-enrolment	12 Weeks Post-enrolment
Informed Consent	X			
Inclusion/Exclusion Criteria	X			
Clinical Assessment	X			
Clinical and Obstetric History	X			
General Questions	X			
Demographics	X			
Primary outcome<br>Oral Health Behaviours toothbrushing	X	X(intervention group only**)	X(intervention group only**)	X
Secondary outcomes<br>Oral Health Behaviour: toothpaste use, sweet drinks, bedtime routine, sweet foods	X			X
Oral Health Self-efficacy	X			X
Invitation to participate in the qualitative evaluation				X

*Outside the scope of this analysis plan.

** Only the intervention group is being asked question about the primary outcome at two additional time points as they are receiving the intervention set of text messages.

2 PATIENT POPULATION

Participants will be parents who have children (0-6years old) who have dental caries (tooth decay). Participants will be recruited from a variety of dental clinics and associated waiting rooms across tertiary and non-tertiary sites within NSW Health. They may be recruited at any stage of their child's treatment journey within NSW Health dental clinics.

2.1 Inclusion criterion

Inclusion criteria are parents/carers of children, where children must have all the following criteria:

1)diagnosis of dental caries (tooth decay),

2)age 0 to 6 years old,

Parents/carers of these children must also own an operational mobile phone, capable of sending and receiving SMS text messages; and provide written informed consent.

2.2 Exclusion criterion

Parents/carers will be excluded if they are: 1) are already participating in a text message-based study on lifestyle risk factors for health, 2) have insufficient English skills to provide informed consent or read and understand the text messages; and 3)are unable to comply with study requirements.

3 ANALYSIS

3.1 Analysis principles

The intention to treat (ITT) analysis will include all randomised patients. All tests will be two-tailed and a p-values of less than 0.05 will be considered statistically significant. Odds ratios (for dichotomous variables) or relative risk ratios (for continuous variables) will be reported with 95% confidence intervals unless stated otherwise.

For descriptive results, means and medians will be reported. Normally distributed continuous variables will be reported as means with a standard deviation. Non-normally distributed variables or dichotomous variables will be expressed as a median with an interquartile range.

3.2 Analysis methodology

Outcomes at 3 months will be compared between the two treatment groups in a regression analysis adjusting for the baseline measures of the outcome. Dichotomous outcomes will use logistic regression, continuous outcomes will use linear regression. Distribution assumptions will be tested, and non-parametric methods will be used if they are violated.

3.3 Data inclusion definitions

We have applied the following observation window definitions at baseline and one, two and three months follow up:

Table 2 Data collection windows for each outcome measure

Randomisation	Data collection instrument provided to participants– post randomisation	Window of acceptable data collection - post randomisation
**Primary outcome**		
Tooth brushing behaviour	12 Weeks	12-20 Weeks
**Secondary outcomes**		
Toothpaste use	12 Weeks	12-20 Weeks
Sweet drinks	12 Weeks	12-20 Weeks
Bedtime milk consumption	12 Weeks	12-20 Weeks
Sweet snacks dietary behaviour	12 Weeks	12-20 Weeks
Water consumption	12 Weeks	12-20 Weeks
Oral health self-efficacy measure	12 Weeks	12-20 Weeks

3.4 Patient flow chart

Figure 1 shows the planned consort diagram which will be presented with the actual numbers in the final analysis.

Figure 1: Consort diagram for the study

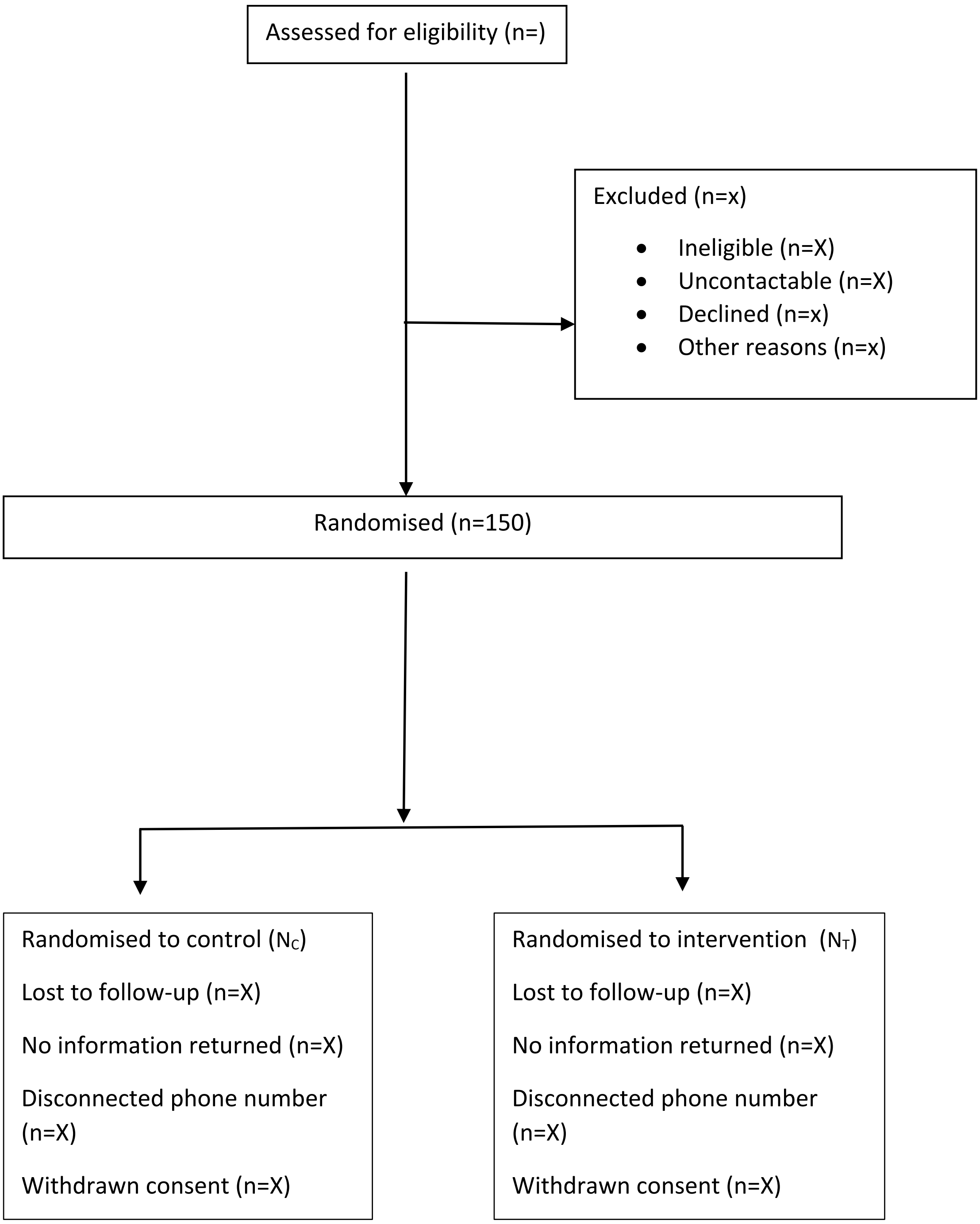

3.5 Summary of patient disposition

A summary of the number of patients randomised and reasons for not completing the study will be reported.

Table 3 Summary of patient disposition

	Control N_C	Intervention N_I	Total N
**Patients randomised**	n/N_C (100%)	n/N_I (100%)	n/N(100%)
**Lost to follow up**	n/N_C (%)	n/N_I (%)	n/N(%)
• **No information returned for the completion survey at the end of the program**	n/N_C (%)	n/N_I (%)	n/N(%)
• **Disconnected phone number**	n/N_C (%)	n/N_I (%)	n/N(%)
• **Withdrawn consent**	n/N_C (%)	n/N_I (%)	n/N(%)

3.6 Data completeness (for primary and secondary data)

A summary of data completeness will be provided in the table below.

Table 4 Summary of data completeness

Completeness of data collection from patients	Control N_C	Intervention N_I	Total N
**Primary outcome (tooth brushing)**			
**Number completed at baseline**	n/N_C (%)	n/N_I (%)	n/N (%)
**Number completed at post intervention**	n/N_C (%)	n/N_I (%)	n/N (%)
**Secondary outcome (Fluoride toothpaste)**			
**Number completed at baseline**	n/N_C (%)	n/N_I (%)	n/N (%)
**Number completed at post intervention**	n/N_C (%)	n/N_I (%)	n/N (%)
**Secondary outcome (Sugary drinks consumption)**			
**Number completed at baseline**	n/N_C (%)	n/N_I (%)	n/N (%)
**Number completed at post intervention**	n/N_C (%)	n/N_I (%)	n/N (%)
**Secondary outcome (Sugary foods consumption)**			
**Number completed at baseline**	n/N_C (%)	n/N_I (%)	n/N (%)
**Number completed at post intervention**	n/N_C (%)	n/N_I (%)	n/N (%)
**Secondary outcome (Water consumption)**			
**Number completed at baseline**	n/N_C (%)	n/N_I (%)	n/N (%)
**Number completed at post intervention**	n/N_C (%)	n/N_I (%)	n/N (%)
**Secondary outcome (Bedtime routine)**			
**Number completed at baseline**	n/N_C (%)	n/N_I (%)	n/N (%)
**Number completed at post intervention**	n/N_C (%)	n/N_I (%)	n/N (%)
**Secondary outcome (Oral health self-efficacy – oral hygiene)**			
**Number completed at baseline**	n/N_C (%)	n/N_I (%)	n/N (%)
**Number completed at post intervention**	n/N_C (%)	n/N_I (%)	n/N (%)
**Secondary outcome (Oral health self-efficacy – diet)**			
**Number completed at baseline**	n/N_C (%)	n/N_I (%)	n/N (%)
**Number completed at post intervention**	n/N_C (%)	n/N_I (%)	n/N (%)
**Secondary outcome (Oral health self-efficacy – bedtime routine)**			
**Number completed at baseline**	n/N_C (%)	n/N_I (%)	n/N (%)
**Number completed at post intervention**	n/N_C (%)	n/N_I (%)	n/N (%)

3.7 Baseline characteristics

All the baseline characteristics will be summarised by treatment group. Where the total possible number of observations are represented as N_C, N_I, N as shown in the header row. Each cell will be formatted with relevant n/ N_C, n/ N_I, or n/ N (%).

Table 5 Baseline demographic characteristics of parents by treatment group

	Control N_C	Intervention N_I	Total N
**Parent's gender**			
a. Male	n/N_C (%)	n/N_I (%)	n/N (%)
b. Female	n/N_C (%)	n/N_I (%)	n/N (%)
c. Other (non-binary)	n/N_C (%)	n/N_I (%)	n/N (%)
d. Other (different term)	n/N_C (%)	n/N_I (%)	n/N (%)
e. Prefer not to say	n/N_C (%)	n/N_I (%)	n/N (%)
**Parent's age**			
a. 18-20	n/N_C (%)	n/N_I (%)	n/N (%)
b. 20-25	n/N_C (%)	n/N_I (%)	n/N (%)
c. 26-35	n/N_C (%)	n/N_I (%)	n/N (%)
d. 36-45	n/N_C (%)	n/N_I (%)	n/N (%)
e. 46 or older	n/N_C (%)	n/N_I (%)	n/N (%)
f. Prefer not to say	n/N_C (%)	n/N_I (%)	n/N (%)
**Parent's level of education**			
a. University degree	n/N_C (%)	n/N_I (%)	n/N (%)
b. TAFE or similar	n/N_C (%)	n/N_I (%)	n/N (%)
c. Year 12	n/N_C (%)	n/N_I (%)	n/N (%)
d. Year 9-11	n/N_C (%)	n/N_I (%)	n/N (%)
e. Year 8 or below	n/N_C (%)	n/N_I (%)	n/N (%)
f. Never attended school	n/N_C (%)	n/N_I (%)	n/N (%)
g. Prefer not to say	n/N_C (%)	n/N_I (%)	n/N (%)
**Country of birth (will be grouped in ABS categories)**			
OCEANIA AND ANTARCTICA	n/N_C* (%)	n/N_I* (%)	n/N* (%)
NORTH-WEST EUROPE	n/N_C* (%)	n/N_I* (%)	n/N* (%)
SOUTHERN AND EASTERN EUROPE	n/N_C* (%)	n/N_I* (%)	n/N* (%)
NORTH AFRICA AND THE MIDDLE EAST	n/N_C* (%)	n/N_I* (%)	n/N* (%)
SOUTH-EAST ASIA	n/N_C* (%)	n/N_I* (%)	n/N* (%)
NORTH-EAST ASIA	n/N_C* (%)	n/N_I* (%)	n/N* (%)
SOUTHERN AND CENTRAL ASIA	n/N_C* (%)	n/N_I* (%)	n/N* (%)
AMERICAS	n/N_C* (%)	n/N_I* (%)	n/N* (%)
SUB-SAHARAN AFRICA	n/N_C* (%)	n/N_I* (%)	n/N* (%)
**Use of oral health apps**			
**a.** Yes	n/N_C (%)	n/N_I (%)	n/N_T (%)
**b.** No	n/N_C (%)	n/N_I (%)	n/N (%)
**Use of other health apps**			
**a.** Yes	n/N_C (%)	n/N_I (%)	n/N_T (%)
**b.** No	n/N_C (%)	n/N_I (%)	n/N (%)
**Local health district where parent was enrolled in**			
a. MLHD	n/N_C (%)	n/N_I (%)	n/N (%)

b. NBMLHD<br>c. NSLHD<br>d. Sydney LHD<br>e. Southern LHD<br>f. WSLHD	n/N_C (%)<br>n/N_C (%)<br>n/N_C (%)<br>n/N_C (%)<br>n/N_C (%)	n/N_I (%)<br>n/N_I (%)<br>n/N_I (%)<br>n/N_I (%)<br>n/N_I (%)	n/N (%)<br>n/N (%)<br>n/N (%)<br>n/N (%)

*N_C ,N_I and N exclude missing data so may differ for compared to the denominator stated for N_C , N_I, N

Table 6 Baseline demographic of child by treatment group

	**Control**<br>N_C	**Intervention**<br>N_I	**Total**<br>N
**Age of the child**	Mean (SD)<br>Median (IQR)<br>(missing=xx)	Mean (SD)<br>Median (IQR)<br>(missing=xx)	Mean (SD)<br>Median (IQR)<br>(missing=xx)
**Modality of care**<br>General Anaesthetic hospital admission(GA)<br>Community dental clinics	 n/N_C (%)<br>n/N_C (%)	 n/N_I (%)<br>n/N_I (%)	 n/N (%)<br>n/N (%)
**Number of siblings (i.e., other) children in the family**<br>1<br>2<br>3<br>4+	 n/N_C (%)<br>n/N_C (%)<br>n/N_C (%)<br>n/N_C (%)	 n/N_I (%)<br>n/N_I (%)<br>n/N_I (%)<br>n/N_I (%)	 n/N (%)<br>n/N (%)<br>n/N (%)<br>n/N (%)
**Birth order of other children***<br>younger than your child having treatment<br>older than your child having treatment<br>I have children that are both older and younger<br>my other child is a twin to the current child<br>not applicable to be (I have one child)	 n/NC* (%)<br>n/NC* (%)<br>n/NC* (%)<br>n/NC* (%)<br>n/NC* (%)	 n/N_I (%)<br>n/N_I (%)<br>n/N_I (%)<br>n/N_I (%)<br>n/N_I (%)	 n/N* (%)<br>n/N* (%)<br>n/N* (%)<br>n/N* (%)<br>n/N* (%)
**Postcode of the child converted to SEIFA scores**<br>1<br>2<br>3<br>4<br>5	 n/N_C (%)<br>n/N_C (%)<br>n/N_C (%)<br>n/N_C (%)<br>n/N_C (%)	 n/N_I (%)<br>n/N_I (%)<br>n/N_I (%)<br>n/N_I (%)<br>n/N_I (%)	 n/N (%)<br>n/N (%)<br>n/N (%)<br>n/N (%)<br>n/N (%)
**Postcode of the child converted to Local health district**<br>MLHD<br>NBMLHD<br>NSLHD<br>SLHD<br>WSLHD	 n/N_C (%)<br>n/N_C (%)<br>n/N_C (%)<br>n/N_C (%)<br>n/N_C (%)	 n/N_I (%)<br>n/N_I (%)<br>n/N_I (%)<br>n/N_I (%)<br>n/N_I (%)	 n/N (%)<br>n/N (%)<br>n/N (%)<br>n/N (%)<br>n/N (%)

N_{C*} ,N_{I*} and N may have multiple entries depending on the number of children in the family so the N will be more than the Nc, n/N_I and N so may differ for each variable.

Table 7 Baseline oral health behaviours by treatment group

	Control N_C	Intervention N_I	Total N
**1.How often do you brush your child's teeth with toothpaste?**			
Don't know /unsure	n/N_C (%)	n/N_I (%)	n/N (%)
Few times a week	n/N_C (%)	n/N_I (%)	n/N (%)
Once a day	n/N_C (%)	n/N_I (%)	n/N (%)
Twice a day	n/N_C (%)	n/N_I (%)	n/N (%)
More than twice a day	n/N_C (%)	n/N_I (%)	n/N (%)
**Tooth brushing (dichotomous)**			
Yes (options d and e) i.e., twice a day or more than twice a day	n/N_C (%)	n/N_I (%)	n/N (%)
No (option a-c)	n/N_C (%)	n/N_I (%)	n/N_T (%)
**2.What type of toothpaste do you use for your child ?**			
Don't know /unsure	n/N_C (%)	n/N_I (%)	n/N (%)
Non-fluoride toothpaste	n/N_C (%)	n/N_I (%)	n/N (%)
Children's fluoride toothpaste	n/N_C (%)	n/N_I (%)	n/N (%)
Standard fluoride toothpaste	n/N_C (%)	n/N_I (%)	n/N (%)
Prescribed toothpaste	n/N_C (%)	n/N_I (%)	n/N (%)
**Fluoride toothpaste**			
Yes (c, d and e)	n/N_C (%)	n/N_I (%)	n/N (%)
No (a-b)	n/N_C (%)	n/N_I (%)	n/N (%)
**3. How often does your child drink sugar sweetened drinks (such as soft drinks, cordials, sports drinks, flavoured milk, fruit juices, energy drinks or iced teas?)**			
Don't know /unsure	n/N_C (%)	n/N_I (%)	n/N (%)
Every day	n/N_C (%)	n/N_I (%)	n/N (%)
A few times a week	n/N_C (%)	n/N_I (%)	n/N (%)
Rarely	n/N_C (%)	n/N_I (%)	n/N (%)
Never	n/N_C (%)	n/N_I (%)	n/N (%)
**Sugar sweetened beverages**			
Yes (d and e)	n/N_C (%)	n/N_I (%)	n/N (%)
No (a, b, and c)	n/N_C (%)	n/N_I (%)	n/N (%)
Decisions for these cut offs are determined by the current ABS stats related to sugar sweetened and diet drink consumption(ABS, 2018).			
**4. How often does your child go to bed with a sippy cup or bottle of milk?**			
Don't know /unsure	n/N_C (%)	n/N_I (%)	n/N (%)

	Control N_C	Intervention N_I	Total N
Every day<br>A few times a week<br>Rarely<br>Never	n/N_C (%)<br>n/N_C (%)<br>n/N_C (%)<br>n/N_C (%)	n/N_I (%)<br>n/N_I (%)<br>n/N_I (%)<br>n/N_I (%)	n/N (%)<br>n/N (%)<br>n/N (%)<br>n/N (%)
**Bedtime routines**<br>Yes (d and e)<br>No (a, b, and c)	 n/N_C (%)<br>n/N_C (%)	 n/N_I (%)<br>n/N_I (%)	 n/N (%)<br>n/N (%)
**5. How often does your child eat sweet foods like chocolates, sweetened cereal, ice-cream, flavoured milk or yoghurt, biscuit, cakes, pastries, or snack/muesli bars?**<br>Don't know /unsure<br>Every day<br>A few times a week<br>Rarely<br>Never	 n/N_C (%)<br>n/N_C (%)<br>n/N_C (%)<br>n/N_C (%)<br>n/N_C (%)	 n/N_I (%)<br>n/N_I (%)<br>n/N_I (%)<br>n/N_I (%)<br>n/N_I (%)	 n/N (%)<br>n/N (%)<br>n/N (%)<br>n/N (%)<br>n/N (%)
**Sugary snack foods**<br>Yes (d and e)<br>No (a, b, and c)	 n/N_C (%)<br>n/N_C (%)	 n/N_I (%)<br>n/N_I (%)	 n/N (%)<br>n/N (%)
**6. How often does your child drink water?**<br>Don't know /unsure<br>Every day<br>A few times a week<br>Rarely<br>Never	 n/N_C (%)<br>n/N_C (%)<br>n/N_C (%)<br>n/N_C (%)<br>n/N_C (%)	 n/N_I (%)<br>n/N_I (%)<br>n/N_I (%)<br>n/N_I (%)<br>n/N_I (%)	 n/N (%)<br>n/N (%)<br>n/N (%)<br>n/N (%)<br>n/N (%)
**Consumption of water**<br>Yes (b)<br>No (a, c, d and e)	 n/N_C (%)<br>n/N_C (%)	 n/N_I (%)<br>n/N_I (%)	 n/N (%)<br>n/N (%)

Table 8 Baseline oral health self-efficacy by treatment group

Both the median and means have been recorded as the data will be descriptive.

	Control N_C	Intervention N_I	Total N
**CAPABILTIY Toothbrushing**			
7. I physically can brush my child's teeth.	Mean (SD)<br>Median<br>(missing=xx)	Mean (SD)<br>(missing=xx)	Mean (SD)<br>(missing=xx)
8. I have all the knowledge I need to brush my child's teeth with a toothbrush and toothpaste.	Mean (SD)<br>(missing=xx)	Mean (SD)<br>(missing=xx)	Mean (SD)<br>(missing=xx)
**OPPORTUNITY Toothbrushing**			

	Control N_C	Intervention N_I	Total N
9. I have all the things (toothbrush and toothpaste) to brush my child's teeth.	Mean (SD) (missing=xx)	Mean (SD) (missing=xx)	Mean (SD) (missing=xx)
10. People around me (friends or family members, in person or online) think it is normal to brush my child's teeth.	Mean (SD) (missing=xx)	Mean (SD) (missing=xx)	Mean (SD) (missing=xx)
**MOTIVATION Toothbrushing**			
11. I am motivated to brush my child's teeth with a toothbrush and toothpaste.	Mean (SD) (missing=xx)	Mean (SD) (missing=xx)	Mean (SD) (missing=xx)
12. Brushing my child's teeth morning and night is something I automatically do without thinking about it.	Mean (SD) (missing=xx)	Mean (SD) (missing=xx)	Mean (SD) (missing=xx)
**CAPABILTIY Food and drinks**			
13. I physically can provide healthy food and drink options for my child.	Mean (SD) (missing=xx)	Mean (SD) (missing=xx)	Mean (SD) (missing=xx)
14. I have all the knowledge I need to provide healthy food and drink options for my child.	Mean (SD) (missing=xx)	Mean (SD) (missing=xx)	Mean (SD) (missing=xx)
**OPPORTUNITY Food and drinks**			
15. I have all the things (water bottle, access to shops with healthy food options) to provide healthy food and drink options for my child.	Mean (SD) (missing=xx)	Mean (SD) (missing=xx)	Mean (SD) (missing=xx)
16. People around me (friends or family members, in person or online) think it is normal to choose healthy food and drink options for my child.	Mean (SD) (missing=xx)	Mean (SD) (missing=xx)	Mean (SD) (missing=xx)
**MOTIVATION Food and drinks**			
17. I am motivated to provide healthy food and drink options for my child.	Mean (SD) (missing=xx)	Mean (SD) (missing=xx)	Mean (SD) (missing=xx)
18. Healthy food and drink options for my child are automatically given without me having to think about it.	Mean (SD) (missing=xx)	Mean (SD) (missing=xx)	Mean (SD) (missing=xx)
**CAPABILITY Bedtime routine**			
19. I physically can maintain a healthy bedtime routine.	Mean (SD) (missing=xx)	Mean (SD) (missing=xx)	Mean (SD) (missing=xx)
20. I have the knowledge to maintain a healthy bedtime routine for my child.	Mean (SD) (missing=xx)	Mean (SD) (missing=xx)	Mean (SD) (missing=xx)
**OPPORTUNITY Bedtime routine**			

	Control N_C	Intervention N_I	Total N
21. I have all the things to maintain a healthy bedtime routine for my child.	Mean (SD) (missing=xx)	Mean (SD) (missing=xx)	Mean (SD) (missing=xx)
22. People around me (friends or family members, in person or online) think it is normal to maintain a healthy bedtime routine for my child.	Mean (SD) (missing=xx)	Mean (SD) (missing=xx)	Mean (SD) (missing=xx)
**MOTIVATION Bedtime routine**			
23. I am motivated to maintain a healthy bedtime routine for my child.	Mean (SD) (missing=xx)	Mean (SD) (missing=xx)	Mean (SD) (missing=xx)
24. A healthy bedtime routine is something I can provide my child without having to think about it.	Mean (SD) (missing=xx)	Mean (SD) (missing=xx)	Mean (SD) (missing=xx)

3.8 Primary analysis:

3.8.1 Primary outcome determination

The **primary outcome is whether tooth brushing requirements have been met.** It will be determined as a binary grouping of the question “How often do you brush your child’s teeth with toothpaste?” at 3 months. A ‘yes’ response will be considered for patients answering, “Twice a day” or “More than twice a day” and a ‘no’ response to those answering, “once a day”, “few times a week”, or “don’t know /unsure”.

3.8.2 Primary outcome analysis

The proportion of parents who meet the primary outcome of toothbrushing requirements at 3 months will be compared by treatment group using a logistic regression model adjusting for whether they had met tooth brushing requirements at baseline and stratification variables (modality of care [GA vs. non-GA] & site [tertiary vs. non-tertiary]). An odds ratio of toothbrushing requirements at 3-months comparing treatment vs. control (reference group) will be presented with a 95% CI as shown in Table 9.

Table 9 Primary outcome analysis of tooth brushing

Met toothbrushing requirements	Control N_C	Intervention N_I	aOR (95% CI)	p-value
**Baseline**	n/N_C (%)	n/N_I (%)		
**3months**	n/N_C (%)	n/N_I (%)	XX.X (XX.X, XX.X)	X.XXX

Additional descriptive granularity of the primary outcomes will be presented in a descriptive table (Table 10).

Table 10 Descriptive Analysis of tooth brushing frequency

Toothbrushing frequency	Control N_C	Intervention N_I
**Baseline**		
Don’t know /unsure	n/N_C (%)	n/N_I (%)
Few times a week	n/N_C (%)	n/N_I (%)

**Toothbrushing frequency**	**Control N_C**	**Intervention N_I**
Once a day	n/N_C (%)	n/N_I (%)
Twice a day	n/N_C (%)	n/N_I (%)
More than twice a day	n/N_C (%)	n/N_I (%)
**3 months**		
Don't know /unsure	n/N_C (%)	n/N_I (%)
Few times a week	n/N_C (%)	n/N_I (%)
Once a day	n/N_C (%)	n/N_I (%)
Twice a day	n/N_C (%)	n/N_I (%)
More than twice a day	n/N_C (%)	n/N_I (%)

3.8.3 Additional analysis related to the primary outcome at 1- 2 months

Additional descriptive analysis will be conducted to assess toothbrushing changes across the time of the trial for the intervention group only. This question was only asked of the intervention group at these additional data collection as theses time points were part of the TextCare intervention (see Table 11).

Table 11 Summary analysis of tooth brushing adherence

**Adherence to toothbrushing**	**Intervention group**
**1 month**	$n/N_{(\text{answered at 1 month})}$ (%)
**2 months**	$n/ N_{(\text{answered at 2 months})}$ (%)
**3 months**	$n/ N_{(\text{answered at 3 months})}$ (%)

3.8.4 Missing data - sensitivity Analysis

Missing data is defined as data that is "missing" or outside the window as defined in Section 3.3 and table 2. There may be data outside the window because the data is self-reported and may be reported late. To assess whether data is missing at random, the pattern of missingness for the primary endpoint will be explored to determine if some baseline characteristics predict missing data at 3 months using logistic regression analysis. If missingness is greater than 5% a sensitivity analysis will be conducted by a worst-case scenario analysis.

3.8.5 Heterogeneity analysis

To assess whether the treatment effect varies between levels of prespecified covariates on the primary efficacy variable of toothbrushing twice a day at 3 months, an interaction of these covariates with the treatment group will be added to a logistic regression.

The model will include:

- Treatment group
- Covariate
- Interaction of covariate and treatment group
- Toothbrushing twice a day at baseline
- Stratification variables (if not the covariate of interest)

If the interaction p-value is less than 0.05 then subgroup analyses will be conducted for that covariate. The baseline covariates to be examined are:

- Age of parent – this would be split by the media age to make it dichotomous, e.g., under 35 or over 35 with middle)
- Education (high school vs. university)
- Site (tertiary hospital/non-tertiary hospital)
- Modality of care (GA/non-GA)
- Ethnicity (Australian versus Other)
- Age of child (over and under 5)
- SEIFA of child (1,2 vs. 3,4,5)
- Other children in family (yes/no)

The odds ratio (dichotomous variable) for each group will be calculated from the above-mentioned models, including the interaction p-value, and presented in a forest plot as exampled in Figure 2.

Figure 2: Example of forest plot presentation of heterogeneity or interaction analysis

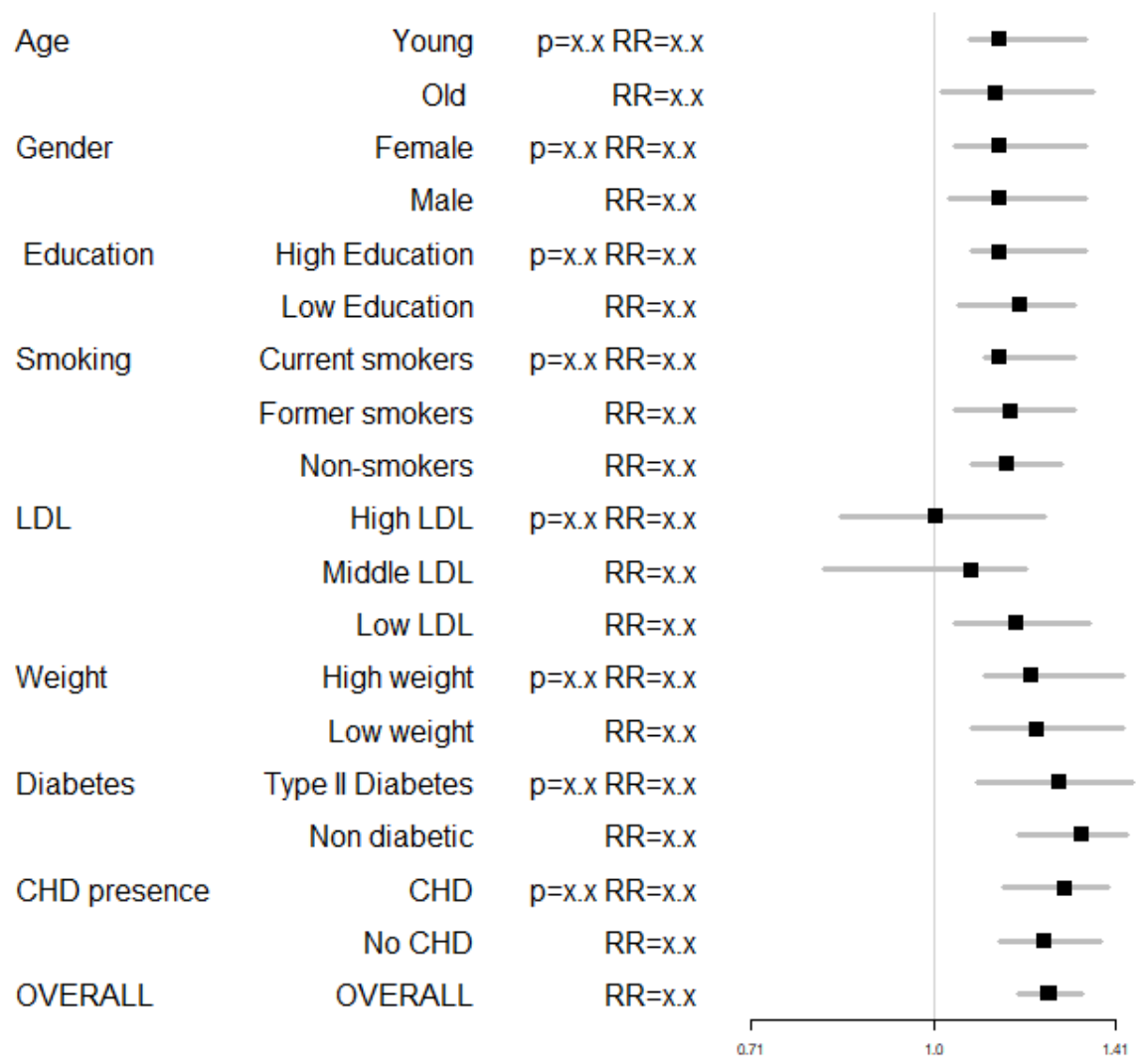

3.8.6 Subgroup analysis

Additional subgroup analysis set a priori based on feasibility and implementation metrics and hypotheses will be performed for:

- Parents who had a child who received treatment via GA or non-GA.
- Parents who were in the "Steps to Smile program" (which is defined as parents of children patients who received a GA procedure and attended Murrumbidgee, Southern and Sydney LHD).

3.9 Secondary analysis of behavioural outcomes

3.9.1 Secondary behavioural outcome determination

The following binary secondary behavioural outcomes are determined as follows:

- **Use of recommended toothpaste type** will be calculated based on the response to the question "What type of toothpaste do you put on our child's toothbrush?" A Yes response will be considered for "Children's fluoride toothpaste", "Standard fluoride toothpaste" or, "Prescribed toothpaste" and a no response to those answering, "Non-fluoride toothpaste" and "Don't know/unsure."

- **Oral health promoting bedtime routines** will be calculated based on the response to the question "How often does your child go to bed with milk in a sippy cup or bottle?" A Yes response will be considered for "rarely" or "never" and a no response for those answering, "Every day" "A few times a week" and "Don't know/unsure."
- **Sugar sweetened beverage consumption** will be calculated based on the response to the question "How often does your child drink sugar sweetened drinks (such as soft drinks, cordials, sports drinks, flavoured milk, fruit juices, energy drinks or iced teas?)" A "yes" response will be considered for "rarely" or "never" and a no response for those answering, "Every day" "A few times a week" and "Don't know/unsure."
- **Sugar-sweetened food consumption** will be calculated based on the response to the question "How often does your child eat sweet foods like chocolates, sweetened cereal, ice-cream, flavoured milk or yoghurt, biscuit, cakes, pastries, or snack/muesli bars?" A "yes" response will be considered for "rarely" or "never" and a no response for those answering, "Every day" "A few times a week" and "Don't know/unsure."
- **water consumption by the child** will be calculated based on the response to the question "How often does your child drink water?" A "yes" response will be considered for "Every day" and a no response for those answering, "A few times a week" "Don't know/unsure" "Rarely" or "Never."

3.9.2 Secondary behavioural outcome analysis

An odds ratio of the secondary behavioural outcomes at 3-months comparing treatment vs. control (reference group) will be estimated with a 95% CI using a logistic regression model adjusting for the relevant baseline behavioural and stratification variables (modality, and site). The format of the secondary outcome analysis will be presented for all secondary behavioural outcomes with an example like that shown in Table12.

Table 12 Secondary outcome analysis for all secondary behavioural outcomes

Met recommended secondary oral health behaviour	Control N_C	Intervention N_I	aOR (95% CI)	p-value
**Baseline**	n/N_C (%)	n/N_I (%)		
**3 months**	n/N_C (%)	n/N_I (%)	XX.X (XX.X, XX.X)	X.XXX

We will also provide granular descriptive details of the secondary outcome measure. All secondary behavioural outcomes will be presented in as shown in Table 13, except for the recommended type of toothpaste as shown in Table 14.

Table 13 Descriptive analysis of secondary outcome behaviours that have frequency categories

Frequency of secondary target behaviour	Control	Intervention
**Baseline**		
**Don't know/unsure**	n/N_C (%)	n/N_I (%)
**Every day**	n/N_C (%)	n/N_I (%)
**A few times a week**	n/N_C (%)	n/N_I (%)
**Rarely**	n/N_C (%)	n/N_I (%)
**Never**	n/N_C (%)	n/N_I (%)
**3 months**		
**Don't know/unsure**	n/N_C (%)	n/N_I (%)

Frequency of secondary target behaviour	Control	Intervention
**Every day**	n/N_C (%)	n/N_I (%)
**A few times a week**	n/N_C (%)	n/N_I (%)
**Rarely**	n/N_C (%)	n/N_I (%)
**Never**	n/N_C (%)	n/N_I (%)

Table 14 Descriptive analysis of toothpaste usage

Toothpaste usage	Control	Intervention
**Baseline**		
**Prescribed toothpaste**	n/N_C (%)	n/N_I (%)
**Standard fluoride toothpaste**	n/N_C (%)	n/N_I (%)
**Children's fluoride toothpaste**	n/N_C (%)	n/N_I (%)
**Non-fluoride toothpaste**	n/N_C (%)	n/N_I (%)
**Don't know/unsure**	n/N_C (%)	n/N_I (%)
**3 months**		
**Prescribed toothpaste**	n/N_C (%)	n/N_I (%)
**Standard fluoride toothpaste**	n/N_C (%)	n/N_I (%)
**Children's fluoride toothpaste**	n/N_C (%)	n/N_I (%)
**Non-fluoride toothpaste**	n/N_C (%)	n/N_I (%)
**Don't know/unsure**	n/N_C (%)	n/N_I (%)

3.10 Secondary analysis of Oral Health Self-efficacy

3.10.1 Secondary Oral Health Self-efficacy determination

Oral Health self-efficacy was assessed using a standardised Capability Opportunity Motivation scale modified from a validated questionnaire from (Keyworth et al., 2020) modified to the dental target behaviours. There are six questions measuring each of the six domains using a 10-point Likert scale. The maximum score was therefore 60 for the 6 questions.

The analysis of Oral Health Self-efficacy was conducted separately for the three-target behaviour the intervention addressed. These three target behaviours were:

- Oral hygiene
- Diet
- Bedtime routine

Oral Hygiene will be defined as the total score out of 60 for the following statements measured on a 10-point Likert scale:

1. Capability (physical): I physically can brush my child's teeth.
2. Capability (psychological): I have all the knowledge I need to brush my child's teeth with a toothbrush and toothpaste.
3. Opportunity (physical): I have all the things (toothbrush and toothpaste) to brush my child's teeth.
4. Opportunity (social): People around me (friends or family members, in person or online) think it is normal to brush my child's teeth.
5. Motivation (reflective): I am motivated to brush my child's teeth with a toothbrush and toothpaste.

6. Motivation (automatic): Brushing my child's teeth morning and night is something I automatically do without thinking about it.

Diet will be defined as the total score out of 60 for the following statements measured on a 10-point Likert scale:

1. Capability (physical): I physically can provide healthy food and drink options for my child.
2. Capability (psychological): I have all the knowledge I need to provide healthy food and drink options for my child.
3. Opportunity (physical): I have all the things (water bottle, access to shops with healthy food options) to provide healthy food and drink options for my child.
4. Opportunity (social): People around me (friends or family members, in person or online) think it is normal to choose healthy food and drink options for my child.
5. Motivation (reflective): I am motivated to provide healthy food and drink options for my child.
6. Motivation (automatic): Healthy food and drink options for my child are automatically given without me having to think about it.

Bedtime routines will be defined as the total score out of 60 for the following statements measured on a 10-point Likert scale:

1. Capability (physical): I physically can maintain a healthy bedtime routine.
2. Capability (psychological): I have the knowledge to maintain a healthy bedtime routine for my child.
3. Opportunity (physical): I have all the things to maintain a healthy bedtime routine for my child.
4. Opportunity (social): People around me (friends or family members, in person or online) think it is normal to maintain a healthy bedtime routine for my child.
5. Motivation (reflective): I am motivated to maintain a healthy bedtime routine for my child.
6. Motivation (automatic): A healthy bedtime routine is something I can provide my child without having to think about it.

3.10.2 Secondary Oral Health Self-efficacy analysis

A regression model for each of the three target behaviours for oral health self-efficacy will be conducted adjusting for the baseline oral health self-efficacy and stratification variables to report the results as shown in Table 15.

Table 15 Analysis of oral health self-efficacy oral hygiene

Composite oral health self-efficacy scores out of 60 for oral hygiene	Control	Intervention	Mean difference (95% CI)	p-value
Baseline Oral Health Self-efficacy (OHSE)	(n=xx)<br>Mean (SD)<br>95% CI	(n=xx)<br>Mean (SD)<br>95% CI	Difference<br>(95% CI)	
OHSE at 3 months	(n=xx)<br>Mean (SD)<br>95% CI	(n=xx)<br>Mean (SD)<br>95% CI	Difference<br>(95% CI)	
Change in OHSE from baseline to 3 months	(n=xx)<br>Mean (SD)<br>95% CI	(n=xx)<br>Mean (SD)<br>95% CI	Difference<br>(95% CI)	

**Adjusted analysis of OHSE at 3 months**	**Mean (SD)<br>95% CI**	**Mean (SD)<br>95% CI**	**Difference**<br>(95% CI)**	**p-value**

**The mean difference is estimated using regression analysis with the baseline value as a covariate and adjusting for site and treatment modality.

Each question will be dichotomised to having a score "greater or equal to 8" on the 10-point Likert scale or "less than 8". The treatment effect on each dichotomised domain will be analysed using a logistic regression and adjusting for the dichotomised domain at baseline and stratification variables. The adjusted odds ratio with a 95% confidence interval will be presented. This will be presented for each of the six domains in Table 16 below.

Table 16 Analysis of each COM-B domain of the for Oral Health Self-Efficacy (OHSE)

**Oral health self-efficacy domain**	**Control<br>N_C**	**Intervention<br>N_I**	**aOR<br>(95% CI)**	**p-value**
**Met the oral health self-efficacy domain**				
**Baseline**	n/N_{CAns} (%)	n/N_{IAns} (%)	1	
**3 months**	n/N_{CAns} (%)	n/N_{IAns} (%)	XX.X (XX.X, XX.X)	X.XXX

N_{CAns} denotes the number of people in the control group who have answered this question. This number will be variable as these questions were option for parents when enrolling in the program.

N_{IAns} denotes the number of people in the text message group who have answered this question. This number will be variable as these questions were option for parents when enrolling in the program.

For each of the 18 domains we will also summarise the results in a descriptive manner for each of the OHSE domains. This information will be presented using the format of the table 17 below.

Table 17 Descriptive analysis of mean for each COMB-B domain for oral health self-efficacy

**Oral health self-efficacy domains**	**Control<br>N**	**Intervention<br>N**
**Met the oral health self-efficacy domain**		
**Baseline**	(n=xx*)<br>Mean (SD)<br>95% CI	(n=xx*)<br>Mean (SD)<br>95% CI
**3 months**	(n=xx*)<br>Mean (SD)<br>95% CI	(n=xx*)<br>Mean (SD)<br>95% CI

* Denotes the number who have answered these questions. This number will be variable as these questions were option for parents when enrolling in the program.

3.11 Safety Analysis

There is not expected to be any safety issues related to the treatment. However, all known representations to the dental clinics will be listed and summarised by treatment group. Any serious adverse events will be reported on.

References List

ABS. (2018, 12/12/2018). Sugar sweetened and diet drink consumption. *Chidren's risk factors* Retrieved from https://www.abs.gov.au/statistics/health/health-conditions-and-risks/childrens-risk-factors/latest-

release#:~:text=Children%20aged%202%2D17%20years%20who%20are%20daily%20consumers%20of,day%20compared%20with%201.6%20cups).

Do, L. G. S. J. A. (2016). *Oral health of Australian Children : The National Child Oral Health Study 2012–14*: University of Adelaide Press.

Keyworth, C., Epton, T., Goldthorpe, J., Calam, R., & Armitage, C. J. (2020). Acceptability, reliability, and validity of a brief measure of capabilities, opportunities, and motivations ("COM-B"). *British journal of health psychology, 25*(3), 474-501. doi:10.1111/bjhp.12417

Tadakamadla, S. K., Rathore, V., Mitchell, A. E., Kaul, A., & Morawska, A. (2022). Child- and family-level factors associated with toothbrushing frequency in a sample of Australian children. *International Journal of Paediatric Dentistry, 32*(5), 639-648. doi:10.1111/ipd.12942

A A A

SMILESup Randomised Control Trial baseline

SMILESup: Supporting parents/carers of young children with tooth decay to use a **M**obile health **I**ntervention targeting oral health **L**ifestyle behaviours and **E**valuating opportunities to **S**cale-**up**: a randomised control trial

**Short Title**	**SMILESup**
**Coordinating Principal Investigator/ Principal Investigator**	*Prof. Clara Chow*

Tooth decay for young children is preventable. The key risk factors for tooth decay are known. Increasing oral health literacy, including knowledge and skills related to good oral health, can prevent tooth decay. Usually, this information is provided to parents/carers at the clinical appointment only.

The SMILESup text message program aims to innovatively provide these oral health messages to parents at home in a timely way (4 times per week). It is a co-designed 3-month text message program to support parents/carers of young children (0 to 6 years old) with tooth decay. You can take part in this research project because you are a parent/carer with a child (0 to 6 years old) who has tooth decay undergoing treatment at NSW public dental clinics.

This project aims to assess whether the SMILESup program supports parents/carers to change oral health behaviors more than usual care. This program may send you text messages to support you in creating healthy habits for your child's oral health.

Which location did your child attend for the dental appointment?
* must provide value

- ○ Sydney Dental Hospital - Sydney Local Health District
- ● Westmead Hospital - Western Sydney Local Health District
- ○ Northern Sydney Local Health District
- ○ Nepean Blue Mountains Local Health District
- ○ Southern Local Health Districts
- ○ Murrumbidgee Local Health Districts

Part 1 What does my participation involve?

1. Introduction

Tooth decay for young children is preventable. The key risk factors for tooth decay are known. Increasing oral health literacy, including knowledge and skills related to good oral health, can prevent tooth decay. Usually, this information is provided to parents/carers at the clinical appointment only.

The SMILESup text message program aims to innovatively provide these oral health messages to parents at home in a timely way (4 times per week). It is a co-designed 3-month text message program to support parents/carers of young children (0 to 6 years old) with tooth decay. You can take part in this research project because you are a parent/carer with a child (0 to 6 years old) who has tooth decay undergoing treatment at NSW public dental clinics.

This project aims to assess whether the SMILESup program supports parents/carers to change oral health behaviors more than usual care. This program may send you text messages to support you in creating healthy habits for your child's oral health.

This Participant Information Sheet/Consent Form tells you about the research project. Knowing what is involved can help you decide if you want to take part in the research.

Please read this information carefully. Ask questions about anything that you don't understand or want to know more about.

Participation in this research is voluntary.

If you decide you want to take part in the research project, you will be asked to sign the consent section. By signing it you are telling us that you:

- Understand what you have read
- Consent to take part in the research project
- Consent to the use of your personal information as described

You will be given a copy of this Participant Information and Consent Form to keep.

2. What is the purpose of this research?

This randomised control trial (RCT) aims to find out whether the SMILESup oral health literacy text message program will improve oral health behaviours of parents/carers, compared to those receiving usual care.

3. What does participation in this research involve?

Taking part involves providing consent at the end of this form, no study activity can happen before this.

We will then ask you to fill in a questionnaire (5-10 mins) about your child's teeth. Some questions will ask about how often tooth brushing happens, how often your child has sugary drinks or snacks, and questions about how confident you are to help them maintain good oral health.

This information will also allow us to tailor the text messages about you and your child.

You will be placed in one of two groups. Group 1 will get text messages 4 per week over the next 3 months. Group 2 will get a message at the start and 1 at the end of the 3-month period with a link to the NSW Health Oral health website. Both groups will still complete the first questionnaire when you first enrol and at 3 months after the program.

Sometimes we don't know which programs are better to support parents/carers caring for their child's oral health. To find out we will compare different groups. Our study is a single-blinded randomised

controlled trial, which means it is not possible to choose the group you are in. A computer system will randomly choose you to get text messages right away (intervention group) or one message then no further messages until after 3 months (control group). You will be assigned a group with the same chance of tossing a coin, and researchers won't know which group you are in.

The following Monday you will get a message to say that you have enrolled into the program.

Whether you are in group 1 or 2 we ask you to reply to text messages asking about tooth brushing habits in the middle of each month. At the end of 3 months, you will also be asked to fill in a final questionnaire to finish the program.

If you are in group 2 (control group), you will still fill in the first questionnaire when you enrol and 3 months after the program. You may choose to get all the text messages after the 3-month study period.

There are no costs with taking part in this research project, nor will you be paid.

4. What do I have to do?

Whether you are in group 1 or 2 we ask you to reply to text messages asking about tooth brushing habits in the middle of each month. At the end of 3 months, you will also be asked to fill in a final questionnaire to finish the program.

The text messages may also prompt you change behaviours that will support your child's smile.

5. Other relevant information about the research project.

You can give feedback at the end of the study. If you are in group1 and have received the SMILESup messages you will be invited to a focus group. Here you can tell us what you liked and didn't like about the program.

6. Do I have to take part in this research project?

Taking part in any research project is voluntary. If you do not wish to take part, you are free to withdraw from the project at any stage.

Your decision whether to take part or not, or to take part and then withdraw, will not affect your child's treatment, or your relationship with those treating your child at NSW public dental clinics.

7. What are the alternatives to participation?

You do not have to take part in this research project to receive treatment at NSW Public dental clinics. Other options are available; these include the current oral health information on the NSW Oral Health website and brochures in the clinic.

8. What are the possible benefits of taking part?

Possible benefits may include the support you receive to change oral health habits for your child. This may include brushing your child's teeth more or swapping sugary drinks with water for your child's oral health.

9. What are the possible risks and disadvantages of taking part?

As the program is only providing health information by text messages, it is very unlikely that there will be major risks or disadvantages of participating in the study.

However, if you become upset or distressed because of your participation in the research, we will offer you support. You will be assisted with arranging appropriate support, this may include counselling. Any

counselling or support will be provided by qualified staff who are not members of the research project team. This counselling will be provided free of charge.

10. What if new information arises during this research project?

Although unlikely if new information related to the SMILESup project becomes available, for example new advice on improving your child's teeth, you will be told about this via the text messages.

11. What if I withdraw from this research project?

If you decide to withdraw from this research project, please notify a member of the research team before you withdraw.

If you withdraw your consent during the research project, the relevant study staff will not collect additional personal information from you, although personal information already collected will be retained to ensure that the results of the research project can be measured properly and to comply with law. You should be aware that some information already collected has been de-identified, we will not be able to identify this information to remove it. Thus, these results may form part of the research project results.

12. Could this research project be stopped unexpectedly?

It is unlikely that this research project may be stopped unexpectedly.

13. What happens when the research project ends?

A summary of the results of the trial including the evaluation will be published 18 months after all data collection. All participants will be notified about the publication.

Part 2 How is the research project being conducted?

14. What will happen to information about me?

By signing the consent form, you consent to the research team collecting and using de-identified personal information about you for the research project. Any information will remain confidential. All participants will be assigned a study ID and will not be identified in the data analysis.

All confidentiality and data security considerations will be in alignment with the University of Sydney and Western Sydney Local Health District data management requirements. Your information will only be used for the purpose of this research project, and it will only be disclosed with your permission, except as required by law.

It is anticipated that the results of this research project will be published and/or presented in a variety of forums. In any publication and/or presentation, information will be de-identified.

In accordance with relevant Australian and/or NSW privacy and other relevant laws, you have the right to request access to the information about you that is collected and stored by the research team. You also have the right to request that any information with which you disagree with be corrected. Please inform the research team member named at the end of this document if you would like access to your information.

15. Complaints and compensation

It is unlikely that you will be distressed in this study. If you become upset because of your participation in the research, we will offer you support. You will be assisted with arranging appropriate support, this

may include counselling. Any support will be provided by qualified staff who are not members of the research project team. Any counselling support will be provided free of charge.

16. Who is organising and funding the research?

This research has been initiated by Prof. Clara Chow. In collaboration with co-investigators: Dr. Harleen Kumar, and PhD researcher Ms. Rebecca Chen.
No member of the research team will receive a personal financial benefit from your involvement in this research project (other than their ordinary wages) [NS 2.2.6 (i)]

17. Who has reviewed the research project?

All research in Australia involving humans is reviewed by an independent group of people called a Human Research Ethics Committee (HREC). The ethical aspects of this research project have been approved by the HREC of Western Sydney Local Health District with a Site-Specific Assessment at Western Sydney Local Health District and Sydney Local Health District.

This project will be carried out according to the *National Statement on Ethical Conduct in Human Research (2007)*. This statement has been developed to protect the interests of people who agree to participate in human research studies.

18. Further information and who to contact

The person you may need to contact will depend on the nature of your query. If you want any further information concerning the SMILE-SUP project or if you have any problems which may be related to your involvement in the project, you can contact the researcher on 8627 3580 or any of the following people: Prof. Clara Chow, Ms. Rebecca Chen and Dr. Neeta Prabhu.

Research contact person: Prof. Clara Chow (Staff Specialist)
Telephone: 02 8890 3125
Email: Clara.chow@health.nsw.gov.au
For matters relating to research at the site at which you are participating, the details of the local site complaints person are below.
Complaints contact person: Research Governance Officer
Telephone: 02 8890 9007
Email: Wslhd-researchoffice@health.nsw.gov.au

If you have any complaints about any aspect of the project, the way it is being conducted or any questions about being a research participant in general, then you may contact:

WSLHD Human Research Ethics Committee contact person: Kellie Hansen
Telephone: 02 8890 9007
Email: Wslhd-researchoffice@health.nsw.gov.au

Site Research Governance office contact: Kellie Hansen
Telephone: 02 8890 9007
Email: Wslhd-researchoffice@health.nsw.gov.au

I have read the Participant Information Sheet or someone has read it to me in a language that I

understand.

I understand the purposes, procedures and risks of the research described in the project.

I have had an opportunity to ask questions and I am satisfied with the answers I have received.

I freely agree to participate in this research project as described and understand that I am free to withdraw at any time during the project without affecting my child's future care.

I understand that I will be given a signed copy of this document to keep.

First name of participant

* must provide value

Last name of participant

* must provide value

Signature

* must provide value

Date

* must provide value

D-M-Y

PICF Westmead Hospital Phase 1 consumer questionnaire Version 1 dated 81222 based on Master V2 1811122

Next Page >>
