# Supplementary material for: Co-Designed Text Messaging Intervention for Parents of Children With Early Childhood Caries: Randomized Controlled Trial
Source: JMIR Mhealth Uhealth. 2026 Nov 3;14:e91720. doi: 10.2196/91720 (PMC13730512; doi:10.2196/91720)
Supplement: Supplementary file 2 [file mhealth-v14-e91720-s002.pdf]

Sample messages from the SmilesUp program

Risk factor	Sample text message
Oral hygiene (tooth brushing)	Have you watched your children brushing their teeth today, did they do a good job? Brushing your teeth together can help them learn great habits @SMILESup
Diet	Top tip of the week: when you go to the shops, try and avoid the snack aisle. Let [insert child's name] help you pick fruits and veggies instead :) @SMILESup
Bedtime routines	A little reminder for you to help [insert child's name] brush their teeth before they go to bed. If they have clean teeth before they sleep, it will prevent tooth decay. @SMILESup
