# Supplementary material for: Co-Designed Text Messaging Intervention for Parents of Children With Early Childhood Caries: Randomized Controlled Trial
Source: JMIR Mhealth Uhealth. 2026 Nov 3;14:e91720. doi: 10.2196/91720 (PMC13730512; doi:10.2196/91720)
Supplement: Supplementary file 3 [file mhealth-v14-e91720-s004.pdf]

CONSORT-EHEALTH (V 1.6.1) - Submission/Publication Form

The CONSORT-EHEALTH checklist is intended for authors of randomized trials evaluating web-based and Internet-based applications/interventions, including mobile interventions, electronic games (incl multiplayer games), social media, certain telehealth applications, and other interactive and/or networked electronic applications. Some of the items (e.g. all subitems under item 5 - description of the intervention) may also be applicable for other study designs.

The goal of the CONSORT EHEALTH checklist and guideline is to be
a) a guide for reporting for authors of RCTs,
b) to form a basis for appraisal of an ehealth trial (in terms of validity)

CONSORT-EHEALTH items/subitems are MANDATORY reporting items for studies published in the Journal of Medical Internet Research and other journals / scientific societies endorsing the checklist.

Items numbered 1., 2., 3., 4a., 4b etc are original CONSORT or CONSORT-NPT (non-pharmacologic treatment) items.
Items with Roman numerals (i., ii, iii, iv etc.) are CONSORT-EHEALTH extensions/clarifications.

As the CONSORT-EHEALTH checklist is still considered in a formative stage, we would ask that you also RATE ON A SCALE OF 1-5 how important/useful you feel each item is FOR THE PURPOSE OF THE CHECKLIST and reporting guideline (optional).

Mandatory reporting items are marked with a red *.
In the textboxes, either copy & paste the relevant sections from your manuscript into this form - please include any quotes from your manuscript in QUOTATION MARKS,
or answer directly by providing additional information not in the manuscript, or elaborating on why the item was not relevant for this study.

YOUR ANSWERS WILL BE PUBLISHED AS A SUPPLEMENTARY FILE TO YOUR PUBLICATION IN JMIR AND ARE CONSIDERED PART OF YOUR PUBLICATION (IF ACCEPTED).
Please fill in these questions diligently. Information will not be copyedited, so please use proper spelling and grammar, use correct capitalization, and avoid abbreviations.

DO NOT FORGET TO SAVE AS PDF _AND_ CLICK THE SUBMIT BUTTON SO YOUR ANSWERS ARE IN OUR DATABASE !!!

Citation Suggestion (if you append the pdf as Appendix we suggest to cite this paper in the caption):
Eysenbach G, CONSORT-EHEALTH Group
CONSORT-EHEALTH: Improving and Standardizing Evaluation Reports of Web-based and Mobile Health Interventions
J Med Internet Res 2011;13(4):e126

URL: http://www.jmir.org/2011/4/e126/
doi: 10.2196/jmir.1923
PMID: 22209829

rebecca.chen@sydney.edu.au Switch account

Draft not saved

Not shared

* Indicates required question

Your name *

First Last

Rebecca Chen

Primary Affiliation (short), City, Country *

University of Toronto, Toronto, Canada

University of Sydney, Sydney, Australia

Your e-mail address *

abc@gmail.com

Rebecca.chen@sydney.edu.au

Title of your manuscript *

Provide the (draft) title of your manuscript.

Co-designed SMS intervention for parents of children with early childhood caries: A randomised controlled trial

Name of your App/Software/Intervention *

If there is a short and a long/alternate name, write the short name first and add the long name in brackets.

SmilesUp

Evaluated Version (if any)

e.g. "V1", "Release 2017-03-01", "Version 2.0.27913"

Your answer

Language(s) *

What language is the intervention/app in? If multiple languages are available, separate by comma (e.g. "English, French")

English

URL of your Intervention Website or App

e.g. a direct link to the mobile app on app in appstore (itunes, Google Play), or URL of the website. If the intervention is a DVD or hardware, you can also link to an Amazon page.

Your answer

URL of an image/screenshot (optional)

Your answer

Accessibility *

Can an enduser access the intervention presently?

- ◯ access is free and open
- ◯ access only for special usergroups, not open
- ◯ access is open to everyone, but requires payment/subscription/in-app purchases
- ◉ app/intervention no longer accessible
- ◯ Other:

Primary Medical Indication/Disease/Condition *

e.g. "Stress", "Diabetes", or define the target group in brackets after the condition, e.g. "Autism (Parents of children with)", "Alzheimers (Informal Caregivers of)"

Early Childhood Dental Caries (Parents of chilc

Primary Outcomes measured in trial *

comma-separated list of primary outcomes reported in the trial

parents brushing their child's teeth twice a day

Secondary/other outcomes

Are there any other outcomes the intervention is expected to affect?

Secondary outcomes were oral health-promoting behaviours: use of fluoride toothpaste, reduced sugared beverage consumption, reduced sweet food consumption, bedtime routines related to the frequency and timing of milk consumption at night, and water consumption.

Recommended "Dose" *

What do the instructions for users say on how often the app should be used?

- ○ Approximately Daily
- ◉ Approximately Weekly
- ○ Approximately Monthly
- ○ Approximately Yearly
- ○ "as needed"
- ○ Other:

Approx. Percentage of Users (starters) still using the app as recommended after 3 months *

- ◉ unknown / not evaluated
- ○ 0-10%
- ○ 11-20%
- ○ 21-30%
- ○ 31-40%
- ○ 41-50%
- ○ 51-60%
- ○ 61-70%
- ○ 71%-80%
- ○ 81-90%
- ○ 91-100%
- ○ Other:

Overall, was the app/intervention effective? *

- ○ yes: all primary outcomes were significantly better in intervention group vs control
- ◉ partly: SOME primary outcomes were significantly better in intervention group vs control
- ○ no statistically significant difference between control and intervention
- ○ potentially harmful: control was significantly better than intervention in one or more outcomes
- ○ inconclusive: more research is needed
- ○ Other:

Article Preparation Status/Stage *

At which stage in your article preparation are you currently (at the time you fill in this form)

- ○ not submitted yet - in early draft status
- ◉ not submitted yet - in late draft status, just before submission
- ○ submitted to a journal but not reviewed yet
- ○ submitted to a journal and after receiving initial reviewer comments
- ○ submitted to a journal and accepted, but not published yet
- ○ published
- ○ Other:

Journal *

If you already know where you will submit this paper (or if it is already submitted), please provide the journal name (if it is not JMIR, provide the journal name under "other")

- [ ] not submitted yet / unclear where I will submit this
- [ ] Journal of Medical Internet Research (JMIR)
- [x] JMIR mHealth and UHealth
- [ ] JMIR Serious Games
- [ ] JMIR Mental Health
- [ ] JMIR Public Health
- [ ] JMIR Formative Research
- [ ] Other JMIR sister journal
- [ ] Other:

Is this a full powered effectiveness trial or a pilot/feasibility trial? *

- [x] Pilot/feasibility
- [ ] Fully powered

Manuscript tracking number *

If this is a JMIR submission, please provide the manuscript tracking number under "other" (The ms tracking number can be found in the submission acknowledgement email, or when you login as author in JMIR. If the paper is already published in JMIR, then the ms tracking number is the four-digit number at the end of the DOI, to be found at the bottom of each published article in JMIR)

- [x] no ms number (yet) / not (yet) submitted to / published in JMIR
- [ ] Other:

TITLE AND ABSTRACT

1a) TITLE: Identification as a randomized trial in the title

1a) Does your paper address CONSORT item 1a? *

I.e does the title contain the phrase "Randomized Controlled Trial"? (if not, explain the reason under "other")

- (selected) yes
- Other:

1a-i) Identify the mode of delivery in the title

Identify the mode of delivery. Preferably use “web-based” and/or “mobile” and/or “electronic game” in the title. Avoid ambiguous terms like “online”, “virtual”, “interactive”. Use “Internet-based” only if Intervention includes non-web-based Internet components (e.g. email), use “computer-based” or “electronic” only if offline products are used. Use “virtual” only in the context of “virtual reality” (3-D worlds). Use “online” only in the context of “online support groups”. Complement or substitute product names with broader terms for the class of products (such as “mobile” or “smart phone” instead of “iphone”), especially if the application runs on different platforms.

	1	2	3	4	5	
subitem not at all important	○	○	○	○	○	essential

Does your paper address subitem 1a-i? *

Copy and paste relevant sections from manuscript title (include quotes in quotation marks "like this" to indicate direct quotes from your manuscript), or elaborate on this item by providing additional information not in the ms, or briefly explain why the item is not applicable/relevant for your study

Co-designed SMS intervention for parents of children with early childhood caries: A randomised controlled trial

1a-ii) Non-web-based components or important co-interventions in title

Mention non-web-based components or important co-interventions in title, if any (e.g., “with telephone support”).

	1	2	3	4	5	
subitem not at all important	○	○	○	○	○	essential

Does your paper address subitem 1a-ii?

Copy and paste relevant sections from manuscript title (include quotes in quotation marks "like this" to indicate direct quotes from your manuscript), or elaborate on this item by providing additional information not in the ms, or briefly explain why the item is not applicable/relevant for your study

Your answer

1a-iii) Primary condition or target group in the title

Mention primary condition or target group in the title, if any (e.g., “for children with Type I Diabetes”) Example: A Web-based and Mobile Intervention with Telephone Support for Children with Type I Diabetes: Randomized Controlled Trial

	1	2	3	4	5	
subitem not at all important	○	○	○	○	○	essential

Does your paper address subitem 1a-iii? *

Copy and paste relevant sections from manuscript title (include quotes in quotation marks "like this" to indicate direct quotes from your manuscript), or elaborate on this item by providing additional information not in the ms, or briefly explain why the item is not applicable/relevant for your study

Co-designed SMS intervention for parents of children with early childhood caries: A randomised controlled trial

1b) ABSTRACT: Structured summary of trial design, methods, results, and conclusions

NPT extension: Description of experimental treatment, comparator, care providers, centers, and blinding status.

1b-i) Key features/functionalities/components of the intervention and comparator in the METHODS section of the ABSTRACT

Mention key features/functionalities/components of the intervention and comparator in the abstract. If possible, also mention theories and principles used for designing the site. Keep in mind the needs of systematic reviewers and indexers by including important synonyms. (Note: Only report in the abstract what the main paper is reporting. If this information is missing from the main body of text, consider adding it)

	1	2	3	4	5	
subitem not at all important	○	○	○	○	○	essential

Does your paper address subitem 1b-i? *

Copy and paste relevant sections from the manuscript abstract (include quotes in quotation marks "like this" to indicate direct quotes from your manuscript), or elaborate on this item by providing additional information not in the ms, or briefly explain why the item is not applicable/relevant for your study

"Background Early childhood caries (ECC) significantly impacts young children's health and quality of life and is preventable through behavioural modification. The WHO Global Health Action Plan (2023-2030) advocates for mHealth programs, to support sustained oral health behaviour change.
Objective: The aim of this study was to assess the effect of a 12-week text message education and support program for parents on managing their child's oral health compared to standard clinical care.
Methods We conducted a multi-site, single-blinded, parallel design randomised controlled trial among parents of children aged 0–6 years who had ECC who accessed services from one of six public dental clinics in metro, regional, and rural locations in NSW, Australia. Participants were randomly assigned to either an intervention group, receiving the fully automated 12-week SMS intervention (SmilesUp), a co-designed program addressing oral health literacy delivered three times a week, or a control group. Both groups continued to receive usual clinical dental treatment. The primary outcome was meeting the target behaviour of the child having their teeth brushed twice a day with fluoride toothpaste, assessed on parental self-report questionnaire. Secondary outcomes included: healthy bedtime routines, frequency of consumption of child's sugar sweetened beverages, and diets low in sugars.
Results 154 participants were enrolled and randomised across six sites, with 124 (80%) completing the follow-up assessment. 96% [73 /76] of intervention participants completed the program. More SmilesUp participants reported brushing their child's teeth twice daily (73.8% vs 66.7%; aOR 2.45 [95% CI, 0.95-6.74], p=0.07), following bedtime routines (88.5% vs 71.4%; aOR 3.25 [95% CI, 1.15-10.18], p=0.03), and having greater knowledge on tooth brushing (92.0% vs 78.8%; aOR 5.47 [95% CI, 1.48-25.19], p=0.02) and knowledge about bedtime routines (91.8% vs 80.8%; aOR 5.76 [95% CI, 1.38-32.16], p=0.03). A reduction in sugar-sweetened food consumption in the intervention group, with boarderline significance (16.4% vs 6.3%; aOR 3.72 [95% CI, 1.02, 17.14], p=0.06). No significant differences were found in sugar-sweetened beverage consumption (41.0% vs. 42.9%; aOR 1.08 [95% CI, 0.49, 2.30], p=0.85).
Conclusions: Most participants completed the SmilesUp mHealth program and together with the findings suggestive of behavioural changes indicate such a program may have positive benefits if provided at scale to parents of children with dental caries. "

1b-ii) Level of human involvement in the METHODS section of the ABSTRACT

Clarify the level of human involvement in the abstract, e.g., use phrases like “fully automated” vs. “therapist/nurse/care provider/physician-assisted” (mention number and expertise of providers involved, if any). (Note: Only report in the abstract what the main paper is reporting. If this information is missing from the main body of text, consider adding it)

	1	2	3	4	5	
subitem not at all important	○	○	○	○	○	essential

Does your paper address subitem 1b-ii?

Copy and paste relevant sections from the manuscript abstract (include quotes in quotation marks "like this" to indicate direct quotes from your manuscript), or elaborate on this item by providing additional information not in the ms, or briefly explain why the item is not applicable/relevant for your study

"Participants were randomly assigned to either an intervention group, receiving the fully automated 12-week SMS intervention (SmilesUp), a co-designed program addressing oral health literacy delivered three times a week, or a control group. "

1b-iii) Open vs. closed, web-based (self-assessment) vs. face-to-face assessments in the METHODS section of the ABSTRACT

Mention how participants were recruited (online vs. offline), e.g., from an open access website or from a clinic or a closed online user group (closed usergroup trial), and clarify if this was a purely web-based trial, or there were face-to-face components (as part of the intervention or for assessment). Clearly say if outcomes were self-assessed through questionnaires (as common in web-based trials). Note: In traditional offline trials, an open trial (open-label trial) is a type of clinical trial in which both the researchers and participants know which treatment is being administered. To avoid confusion, use “blinded” or “unblinded” to indicated the level of blinding instead of “open”, as “open” in web-based trials usually refers to “open access” (i.e. participants can self-enrol). (Note: Only report in the abstract what the main paper is reporting. If this information is missing from the main body of text, consider adding it)

	1	2	3	4	5	
subitem not at all important	○	○	○	○	○	essential

Does your paper address subitem 1b-iii?

Copy and paste relevant sections from the manuscript abstract (include quotes in quotation marks "like this" to indicate direct quotes from your manuscript), or elaborate on this item by providing additional information not in the ms, or briefly explain why the item is not applicable/relevant for your study

"The primary outcome was meeting the target behaviour of the child having their teeth brushed twice a day with fluoride toothpaste, assessed on parental self-report questionnaire. "

1b-iv) RESULTS section in abstract must contain use data

Report number of participants enrolled/assessed in each group, the use/uptake of the intervention (e.g., attrition/adherence metrics, use over time, number of logins etc.), in addition to primary/secondary outcomes. (Note: Only report in the abstract what the main paper is reporting. If this information is missing from the main body of text, consider adding it)

	1	2	3	4	5	
subitem not at all important	○	○	○	○	○	essential

Does your paper address subitem 1b-iv?

Copy and paste relevant sections from the manuscript abstract (include quotes in quotation marks "like this" to indicate direct quotes from your manuscript), or elaborate on this item by providing additional information not in the ms, or briefly explain why the item is not applicable/relevant for your study

"154 participants were enrolled and randomised across six sites, with 124 (80%) completing the follow-up assessment. 96% [73 /76] of intervention participants completed the program. More SmilesUp participants reported brushing their child's teeth twice daily (73.8% vs 66.7%; aOR 2.45 [95% CI, 0.95-6.74], p=0.07), following bedtime routines (88.5% vs 71.4%; aOR 3.25 [95% CI, 1.15-10.18], p=0.03), and having greater knowledge on tooth brushing (92.0% vs 78.8%; aOR 5.47 [95% CI, 1.48-25.19], p=0.02) and knowledge about bedtime routines (91.8% vs 80.8%; aOR 5.76 [95% CI, 1.38-32.16], p=0.03). A reduction in sugar-sweetened food consumption in the intervention group, with boarderline significance (16.4% vs 6.3%; aOR 3.72 [95% CI, 1.02, 17.14], p=0.06). No significant differences were found in sugar-sweetened beverage consumption (41.0% vs. 42.9%; aOR 1.08 [95% CI, 0.49, 2.30], p=0.85)."

1b-v) CONCLUSIONS/DISCUSSION in abstract for negative trials

Conclusions/Discussions in abstract for negative trials: Discuss the primary outcome - if the trial is negative (primary outcome not changed), and the intervention was not used, discuss whether negative results are attributable to lack of uptake and discuss reasons. (Note: Only report in the abstract what the main paper is reporting. If this information is missing from the main body of text, consider adding it)

	1	2	3	4	5	
subitem not at all important	○	○	○	○	○	essential

Does your paper address subitem 1b-v?

Copy and paste relevant sections from the manuscript abstract (include quotes in quotation marks "like this" to indicate direct quotes from your manuscript), or elaborate on this item by providing additional information not in the ms, or briefly explain why the item is not applicable/relevant for your study

"Conclusions: Most participants completed the SmilesUp mHealth program and together with the findings, suggestive of behavioural changes indicate such a program may have positive benefits if provided at scale to parents of children with dental caries. "

INTRODUCTION

2a) In INTRODUCTION: Scientific background and explanation of rationale

2a-i) Problem and the type of system/solution

Describe the problem and the type of system/solution that is object of the study: intended as stand-alone intervention vs. incorporated in broader health care program? Intended for a particular patient population? Goals of the intervention, e.g., being more cost-effective to other interventions, replace or complement other solutions? (Note: Details about the intervention are provided in “Methods” under 5)

	1	2	3	4	5	
subitem not at all important	○	○	○	○	○	essential

Does your paper address subitem 2a-i? *

Copy and paste relevant sections from the manuscript (include quotes in quotation marks "like this" to indicate direct quotes from your manuscript), or elaborate on this item by providing additional information not in the ms, or briefly explain why the item is not applicable/relevant for your study

"Traditional chairside advice and demonstration have limited effectiveness [8] because behaviour change requires repeated, practical, and accessible prompts that build parental oral health literacy and capability within the home environment [9].

Recognising this gap, the WHO Global Health Action Plan (2023–2030) [10] advocates the use of digital innovations, including mobile health (mHealth). mHealth interventions are scalable, accessible, and effective across a range of non-communicable diseases, yet their use in early childhood oral health remains emerging [11]. Existing programs largely target older adults and adolescents, focus narrowly on toothbrushing, and are rarely grounded in behavioural theory [12, 13]. A recent systematic review has identified only a limited number of trials that have tested mHealth interventions targeting parents with young children for ECC prevention [14].
"

2a-ii) Scientific background, rationale: What is known about the (type of) system

Scientific background, rationale: What is known about the (type of) system that is the object of the study (be sure to discuss the use of similar systems for other conditions/diagnoses, if appropiate), motivation for the study, i.e. what are the reasons for and what is the context for this specific study, from which stakeholder viewpoint is the study performed, potential impact of findings [2]. Briefly justify the choice of the comparator.

	1	2	3	4	5	
subitem not at all important	○	○	○	○	○	essential

Does your paper address subitem 2a-ii? *

Copy and paste relevant sections from the manuscript (include quotes in quotation marks "like this" to indicate direct quotes from your manuscript), or elaborate on this item by providing additional information not in the ms, or briefly explain why the item is not applicable/relevant for your study

"Recognising this gap, the WHO Global Health Action Plan (2023–2030) [10] advocates the use of digital innovations, including mobile health (mHealth). mHealth interventions are scalable, accessible, and effective across a range of non-communicable diseases, yet their use in early childhood oral health remains emerging [11]. Existing programs largely target older adults and adolescents, focus narrowly on toothbrushing, and are rarely grounded in behavioural theory [12, 13]. A recent systematic review has identified only a limited number of trials that have tested mHealth interventions targeting parents with young children for ECC prevention [14]. To address these limitations, our research group undertook a comprehensive co-design process with parents to develop a the SmilesUp intervention which is a theory-informed mHealth intervention tailored to their needs, preferences, and identified barriers and enablers [15]. The SmilesUp program enhances parental oral health literacy by providing tailored information, including practical video demonstrations, and timely prompts delivered directly within the home environment where oral health habits are formed. "

2b) In INTRODUCTION: Specific objectives or hypotheses

Does your paper address CONSORT subitem 2b? *

Copy and paste relevant sections from the manuscript (include quotes in quotation marks "like this" to indicate direct quotes from your manuscript), or elaborate on this item by providing additional information not in the ms, or briefly explain why the item is not applicable/relevant for your study

"This randomised controlled trial aims to evaluate the impact of SmilesUp on parental oral health behaviours, knowledge, oral health self-efficacy (capability, opportunity, and motivation), and overall engagement, addressing a critical gap in mHealth strategies to prevent early childhood caries."

METHODS

3a) Description of trial design (such as parallel, factorial) including allocation ratio

Does your paper address CONSORT subitem 3a? *

Copy and paste relevant sections from the manuscript (include quotes in quotation marks "like this" to indicate direct quotes from your manuscript), or elaborate on this item by providing additional information not in the ms, or briefly explain why the item is not applicable/relevant for your study

"A single-blinded parallel, randomised controlled trial (RCT) (1:1 allocation ratio)."

3b) Important changes to methods after trial commencement (such as eligibility criteria), with reasons

Does your paper address CONSORT subitem 3b? *

Copy and paste relevant sections from the manuscript (include quotes in quotation marks "like this" to indicate direct quotes from your manuscript), or elaborate on this item by providing additional information not in the ms, or briefly explain why the item is not applicable/relevant for your study

no changes to the methods.

3b-i) Bug fixes, Downtimes, Content Changes

Bug fixes, Downtimes, Content Changes: ehealth systems are often dynamic systems. A description of changes to methods therefore also includes important changes made on the intervention or comparator during the trial (e.g., major bug fixes or changes in the functionality or content) (5-iii) and other “unexpected events” that may have influenced study design such as staff changes, system failures/downtimes, etc. [2].

	1	2	3	4	5	
subitem not at all important	○	○	○	○	○	essential

Does your paper address subitem 3b-i?

Copy and paste relevant sections from the manuscript (include quotes in quotation marks "like this" to indicate direct quotes from your manuscript), or elaborate on this item by providing additional information not in the ms, or briefly explain why the item is not applicable/relevant for your study

Your answer

4a) Eligibility criteria for participants

Does your paper address CONSORT subitem 4a? *

Copy and paste relevant sections from the manuscript (include quotes in quotation marks "like this" to indicate direct quotes from your manuscript), or elaborate on this item by providing additional information not in the ms, or briefly explain why the item is not applicable/relevant for your study

"Parents of children with ECC, who are either awaiting or undergoing dental treatment within the NSW public dental service in Australia, were approached face-to-face for the study within community dental clinics at six sites across metropolitan, rural, and regional areas. These areas collectively serve a population of approximately four million people and cover an area of 150,000 sqm. All clinics primarily offer a fee-free dental service for families unable to afford private care, with clinical and preventive care guidelines based on existing NSW public dental policies [17]. All participants were given access to a participant Information and consent form, prior to enrolment. Eligibility included parents of children who have all the following: 1) their child has a clinical diagnosis of dental caries (tooth decay), 2) age 0 to 6 years inclusive, and 3) waiting (or have attended) a public NSW dental service for dental caries (tooth decay). Only one child per family was recruited. Parents will be excluded if they 1) are already participating in a text message-based study on lifestyle risk factors for health, 2) did not have proficient English skills to provide written informed consent or 3) are unable to comply with study requirements. Parents of these children must also have basic computer/smartphone literacy and own an operational mobile phone capable of sending and receiving SMS text messages, accessing the internet. "

4a-i) Computer / Internet literacy

Computer / Internet literacy is often an implicit “de facto” eligibility criterion - this should be explicitly clarified.

	1	2	3	4	5	
subitem not at all important	○	○	○	○	○	essential

Does your paper address subitem 4a-i?

Copy and paste relevant sections from the manuscript (include quotes in quotation marks "like this" to indicate direct quotes from your manuscript), or elaborate on this item by providing additional information not in the ms, or briefly explain why the item is not applicable/relevant for your study

"Parents of these children must also have basic computer/smartphone literacy and own an operational mobile phone capable of sending and receiving SMS text messages, accessing the internet."

4a-ii) Open vs. closed, web-based vs. face-to-face assessments:

Open vs. closed, web-based vs. face-to-face assessments: Mention how participants were recruited (online vs. offline), e.g., from an open access website or from a clinic, and clarify if this was a purely web-based trial, or there were face-to-face components (as part of the intervention or for assessment), i.e., to what degree got the study team to know the participant. In online-only trials, clarify if participants were quasi-anonymous and whether having multiple identities was possible or whether technical or logistical measures (e.g., cookies, email confirmation, phone calls) were used to detect/prevent these.

	1	2	3	4	5	
subitem not at all important	○	○	○	○	○	essential

Does your paper address subitem 4a-ii? *

Copy and paste relevant sections from the manuscript (include quotes in quotation marks "like this" to indicate direct quotes from your manuscript), or elaborate on this item by providing additional information not in the ms, or briefly explain why the item is not applicable/relevant for your study

"Parents of children with ECC, who are either awaiting or undergoing dental treatment within the NSW public dental service in Australia, were approached face-to-face for the study within community dental clinics at six sites across metropolitan, rural, and regional areas. "

4a-iii) Information giving during recruitment

Information given during recruitment. Specify how participants were briefed for recruitment and in the informed consent procedures (e.g., publish the informed consent documentation as appendix, see also item X26), as this information may have an effect on user self-selection, user expectation and may also bias results.

	1	2	3	4	5	
subitem not at all important	○	○	○	○	○	essential

Does your paper address subitem 4a-iii?

Copy and paste relevant sections from the manuscript (include quotes in quotation marks "like this" to indicate direct quotes from your manuscript), or elaborate on this item by providing additional information not in the ms, or briefly explain why the item is not applicable/relevant for your study

"All participants were given access to a participant Information and consent form (Appendix X), prior to enrolment."

4b) Settings and locations where the data were collected

Does your paper address CONSORT subitem 4b? *

Copy and paste relevant sections from the manuscript (include quotes in quotation marks "like this" to indicate direct quotes from your manuscript), or elaborate on this item by providing additional information not in the ms, or briefly explain why the item is not applicable/relevant for your study

"Parents of children with ECC, who are either awaiting or undergoing dental treatment within the NSW public dental service in Australia, were approached face-to-face for the study within community dental clinics at six sites across metropolitan, rural, and regional areas. These areas collectively serve a population of approximately four million people and cover an area of 150,000 sqm. "

4b-i) Report if outcomes were (self-)assessed through online questionnaires

Clearly report if outcomes were (self-)assessed through online questionnaires (as common in web-based trials) or otherwise.

	1	2	3	4	5	
subitem not at all important	○	○	○	○	○	essential

Does your paper address subitem 4b-i? *

Copy and paste relevant sections from the manuscript (include quotes in quotation marks "like this" to indicate direct quotes from your manuscript), or elaborate on this item by providing additional information not in the ms, or briefly explain why the item is not applicable/relevant for your study

"Outcomes were ascertained through parental self-report using a standardised NSW Health questionnaire [23]. "

4b-ii) Report how institutional affiliations are displayed

Report how institutional affiliations are displayed to potential participants [on ehealth media], as affiliations with prestigious hospitals or universities may affect volunteer rates, use, and reactions with regards to an intervention.(Not a required item – describe only if this may bias results)

	1	2	3	4	5	
subitem not at all important	○	○	○	○	○	essential

Does your paper address subitem 4b-ii?

Copy and paste relevant sections from the manuscript (include quotes in quotation marks "like this" to indicate direct quotes from your manuscript), or elaborate on this item by providing additional information not in the ms, or briefly explain why the item is not applicable/relevant for your study

Appendix item - where we provide a copy of our Participant Information and Consent forms.

5) The interventions for each group with sufficient details to allow replication, including how and when they were actually administered

5-i) Mention names, credential, affiliations of the developers, sponsors, and owners

Mention names, credential, affiliations of the developers, sponsors, and owners [6] (if authors/evaluators are owners or developer of the software, this needs to be declared in a “Conflict of interest” section or mentioned elsewhere in the manuscript).

	1	2	3	4	5	
subitem not at all important	○	○	○	○	○	essential

Does your paper address subitem 5-i?

Copy and paste relevant sections from the manuscript (include quotes in quotation marks "like this" to indicate direct quotes from your manuscript), or elaborate on this item by providing additional information not in the ms, or briefly explain why the item is not applicable/relevant for your study

"Participants received 36 semi-personalised messages (three per week) delivered through the TextCare™ platform via an automated and pre-programmed schedule shaped by consumer engagement. " Further information about TextCare is stated in out ethical protcol which is an Appendix item.

5-ii) Describe the history/development process

Describe the history/development process of the application and previous formative evaluations (e.g., focus groups, usability testing), as these will have an impact on adoption/use rates and help with interpreting results.

	1	2	3	4	5	
subitem not at all important	○	○	○	○	○	essential

Does your paper address subitem 5-ii?

Copy and paste relevant sections from the manuscript (include quotes in quotation marks "like this" to indicate direct quotes from your manuscript), or elaborate on this item by providing additional information not in the ms, or briefly explain why the item is not applicable/relevant for your study

This has been previously published in JMIR Pediatrics and Parenting. "The SmilesUp intervention is a 12-week SMS program co-designed with parents and dental practitioners to target key oral health behaviours, including toothbrushing with fluoride toothpaste, bedtime routines, and consumption of sugar-sweetened foods and drinks [15]. The intervention aligns with WHO guidance to improve oral health literacy for behaviour change and is informed by the Behaviour Change Wheel (BCW) theory [20]. The BCW synthesises 19 major behaviour change theories to identify the capabilities, opportunities and motivations required and the most appropriate intervention functions to support effective behaviour change [21, 22]. Messages aim to improve oral health literacy and provide brief reminders, reassurance, and links to smartphone-accessible video resources (Appendix X)."

5-iii) Revisions and updating

Revisions and updating. Clearly mention the date and/or version number of the application/intervention (and comparator, if applicable) evaluated, or describe whether the intervention underwent major changes during the evaluation process, or whether the development and/or content was “frozen” during the trial. Describe dynamic components such as news feeds or changing content which may have an impact on the replicability of the intervention (for unexpected events see item 3b).

	1	2	3	4	5	
subitem not at all important	○	○	○	○	○	essential

Does your paper address subitem 5-iii?

Copy and paste relevant sections from the manuscript (include quotes in quotation marks "like this" to indicate direct quotes from your manuscript), or elaborate on this item by providing additional information not in the ms, or briefly explain why the item is not applicable/relevant for your study

Your answer

5-iv) Quality assurance methods

Provide information on quality assurance methods to ensure accuracy and quality of information provided [1], if applicable.

	1	2	3	4	5	
subitem not at all important	○	○	○	○	○	essential

Does your paper address subitem 5-iv?

Copy and paste relevant sections from the manuscript (include quotes in quotation marks "like this" to indicate direct quotes from your manuscript), or elaborate on this item by providing additional information not in the ms, or briefly explain why the item is not applicable/relevant for your study

Your answer

5-v) Ensure replicability by publishing the source code, and/or providing screenshots/screen-capture video, and/or providing flowcharts of the algorithms used

Ensure replicability by publishing the source code, and/or providing screenshots/screen-capture video, and/or providing flowcharts of the algorithms used. Replicability (i.e., other researchers should in principle be able to replicate the study) is a hallmark of scientific reporting.

	1	2	3	4	5	
subitem not at all important	○	○	○	○	○	essential

Does your paper address subitem 5-v?

Copy and paste relevant sections from the manuscript (include quotes in quotation marks "like this" to indicate direct quotes from your manuscript), or elaborate on this item by providing additional information not in the ms, or briefly explain why the item is not applicable/relevant for your study

Your answer

5-vi) Digital preservation

Digital preservation: Provide the URL of the application, but as the intervention is likely to change or disappear over the course of the years; also make sure the intervention is archived (Internet Archive, webcitation.org, and/or publishing the source code or screenshots/videos alongside the article). As pages behind login screens cannot be archived, consider creating demo pages which are accessible without login.

	1	2	3	4	5	
subitem not at all important	○	○	○	○	○	essential

Does your paper address subitem 5-vi?

Copy and paste relevant sections from the manuscript (include quotes in quotation marks "like this" to indicate direct quotes from your manuscript), or elaborate on this item by providing additional information not in the ms, or briefly explain why the item is not applicable/relevant for your study

Your answer

5-vii) Access

Access: Describe how participants accessed the application, in what setting/context, if they had to pay (or were paid) or not, whether they had to be a member of specific group. If known, describe how participants obtained “access to the platform and Internet” [1]. To ensure access for editors/reviewers/readers, consider to provide a “backdoor” login account or demo mode for reviewers/readers to explore the application (also important for archiving purposes, see vi).

	1	2	3	4	5	
subitem not at all important	○	○	○	○	○	essential

Does your paper address subitem 5-vii? *

Copy and paste relevant sections from the manuscript (include quotes in quotation marks "like this" to indicate direct quotes from your manuscript), or elaborate on this item by providing additional information not in the ms, or briefly explain why the item is not applicable/relevant for your study

"Participants directly received 36 semi-personalised messages (three per week) to their mobile phones, delivered through the TextCare™ platform via an automated and pre-programmed schedule shaped by consumer engagement. Personalisation included the child’s name and parents’ choice of morning or evening delivery. "

5-viii) Mode of delivery, features/functionalities/components of the intervention and comparator, and the theoretical framework

Describe mode of delivery, features/functionalities/components of the intervention and comparator, and the theoretical framework [6] used to design them (instructional strategy [1], behaviour change techniques, persuasive features, etc., see e.g., [7, 8] for terminology). This includes an in-depth description of the content (including where it is coming from and who developed it) [1],” whether [and how] it is tailored to individual circumstances and allows users to track their progress and receive feedback” [6]. This also includes a description of communication delivery channels and – if computer-mediated communication is a component – whether communication was synchronous or asynchronous [6]. It also includes information on presentation strategies [1], including page design principles, average amount of text on pages, presence of hyperlinks to other resources, etc. [1].

	1	2	3	4	5	
subitem not at all important	○	○	○	○	○	essential

Does your paper address subitem 5-viii? *

Copy and paste relevant sections from the manuscript (include quotes in quotation marks "like this" to indicate direct quotes from your manuscript), or elaborate on this item by providing additional information not in the ms, or briefly explain why the item is not applicable/relevant for your study

"The intervention aligns with WHO guidance to improve oral health literacy for behaviour change and is informed by the Behaviour Change Wheel (BCW) theory [20]. The BCW synthesises 19 major behaviour change theories to identify the capabilities, opportunities and motivations required and the most appropriate intervention functions to support effective behaviour change [21, 22]. Messages aim to improve oral health literacy and provide brief reminders, reassurance, and links to smartphone-accessible video resources (Appendix X).

Participants directly received 36 semi-personalised messages (three per week) to their mobile phones, delivered through the TextCare™ platform via an automated and pre-programmed schedule shaped by consumer engagement. Personalisation included the child’s name and parents’ choice of morning or evening delivery."

5-ix) Describe use parameters

Describe use parameters (e.g., intended “doses” and optimal timing for use). Clarify what instructions or recommendations were given to the user, e.g., regarding timing, frequency, heaviness of use, if any, or was the intervention used ad libitum.

	1	2	3	4	5	
subitem not at all important	○	○	○	○	○	essential

Does your paper address subitem 5-ix?

Copy and paste relevant sections from the manuscript (include quotes in quotation marks "like this" to indicate direct quotes from your manuscript), or elaborate on this item by providing additional information not in the ms, or briefly explain why the item is not applicable/relevant for your study

Your answer

5-x) Clarify the level of human involvement

Clarify the level of human involvement (care providers or health professionals, also technical assistance) in the e-intervention or as co-intervention (detail number and expertise of professionals involved, if any, as well as “type of assistance offered, the timing and frequency of the support, how it is initiated, and the medium by which the assistance is delivered”. It may be necessary to distinguish between the level of human involvement required for the trial, and the level of human involvement required for a routine application outside of a RCT setting (discuss under item 21 – generalizability).

	1	2	3	4	5	
subitem not at all important	○	○	○	○	○	essential

Does your paper address subitem 5-x?

Copy and paste relevant sections from the manuscript (include quotes in quotation marks "like this" to indicate direct quotes from your manuscript), or elaborate on this item by providing additional information not in the ms, or briefly explain why the item is not applicable/relevant for your study

"The program was primarily one-way, but allowed two-way communication monitored asynchronously by a clinician-researcher. Participants needing clinical assistance receive automated messages that contain the contact details for their nearest NSW public dental clinic or emergency services if the dental concern is urgent. Feedback on the program is monitored asynchronously by a clinician-researcher, with additional feedback collected at the 12-week follow-up.

The SmilesUp program was implemented alongside standard clinical dental care. The control group received standard care only, consisting of clinical assessment, treatment, and preventative advice provided by a dental practitioner. After completing the study period, parents in the control group were offered access to the SmilesUp intervention."

5-xi) Report any prompts/reminders used

Report any prompts/reminders used: Clarify if there were prompts (letters, emails, phone calls, SMS) to use the application, what triggered them, frequency etc. It may be necessary to distinguish between the level of prompts/reminders required for the trial, and the level of prompts/reminders for a routine application outside of a RCT setting (discuss under item 21 – generalizability).

	1	2	3	4	5	
subitem not at all important	○	○	○	○	○	essential

Does your paper address subitem 5-xi? *

Copy and paste relevant sections from the manuscript (include quotes in quotation marks "like this" to indicate direct quotes from your manuscript), or elaborate on this item by providing additional information not in the ms, or briefly explain why the item is not applicable/relevant for your study

"Participants directly received 36 semi-personalised messages (three per week) to their mobile phones, delivered through the TextCare™ platform via an automated and pre-programmed schedule shaped by consumer engagement. Personalisation included the child's name and parents' choice of morning or evening delivery. The program was primarily one-way, but allowed two-way communication monitored asynchronously by a clinician-researcher. "

5-xii) Describe any co-interventions (incl. training/support)

Describe any co-interventions (incl. training/support): Clearly state any interventions that are provided in addition to the targeted eHealth intervention, as ehealth intervention may not be designed as stand-alone intervention. This includes training sessions and support [1]. It may be necessary to distinguish between the level of training required for the trial, and the level of training for a routine application outside of a RCT setting (discuss under item 21 – generalizability.

	1	2	3	4	5	
subitem not at all important	○	○	○	○	○	essential

Does your paper address subitem 5-xii? *

Copy and paste relevant sections from the manuscript (include quotes in quotation marks "like this" to indicate direct quotes from your manuscript), or elaborate on this item by providing additional information not in the ms, or briefly explain why the item is not applicable/relevant for your study

"The SmilesUp program was implemented alongside standard clinical dental care. The control group received standard care only, consisting of clinical assessment, treatment, and preventative advice provided by a dental practitioner. "

6a) Completely defined pre-specified primary and secondary outcome measures, including how and when they were assessed

Does your paper address CONSORT subitem 6a? *

Copy and paste relevant sections from the manuscript (include quotes in quotation marks "like this" to indicate direct quotes from your manuscript), or elaborate on this item by providing additional information not in the ms, or briefly explain why the item is not applicable/relevant for your study

"Outcomes
The primary outcome was meeting the target behaviour of the child having their teeth brushed twice a day with fluoride toothpaste. Secondary outcomes were oral health-promoting behaviours: use of fluoride toothpaste, reduced sugared beverage consumption, reduced sweet food consumption, bedtime routines related to the frequency and timing of milk consumption at night, and water consumption. Outcomes were ascertained through parental self-report using a standardised NSW Health questionnaire [23]. The primary outcome was met if participants responded yes to either “Twice a day” or “More than twice a day” and otherwise no. Definitions for primary and secondary behavioural outcomes are described in (Appendix p.35-36, 40).

Additional outcomes of Oral Health self-efficacy were assessed through questionnaire, with these questions being optional to reduce survey fatigue for participants to complete at enrolment. Three target behaviours (oral hygiene, diet, and bedtime routines) were assessed via six questions covering 1) physical capability, 2) psychological capability i.e. knowledge capability), 3) physical opportunity, 4) social opportunity, 5) reflective motivation, and 6) automatic motivation [9] (see Appendix p. 42). Acceptance of the program was also assessed by the number of participants who completed the SmilesUp text message intervention.

Statistical Analysis

A sample size of 150 participants will provide 80% power to detect a 25% increase of the target toothbrushing behaviour in the intervention group from 53% to 78% compared to the control group at three months, considering a 5% significance level and a 25% dropout rate. A cross-sectional study of a similar Australian population accessing public dental services found that 53% of children aged 3-8 brushed twice a day with toothpaste [24].

Our statistical analysis followed an intention-to-treat principle and a pre-specified statistical analysis plan. To assess the effectiveness of the intervention, a logistic regression model, adjusting for whether parents are toothbrushing for their child twice a day with toothpaste at baseline and the stratification variables will be used, an adjusted Odds Ratio (aOR) with 95% confidence intervals (CI) will be reported. Similarly, all secondary behavioural outcomes will be analysed using the same logistic regression model. A statistical significance level of 0.05 will be used. Interaction between treatment and pre-specified covariates (planned dental procedure requiring GA vs. non-GA) and (tertiary vs. non-tertiary sites) was conducted to explore the heterogeneity of our results. No multiplicity adjustments will be performed for secondary outcomes, as they are exploratory by nature. If missingness is above 10% we will assess missingness at random for the primary outcome using relevant t-tests or chi-square tests for the relevant covariate. We did not conduct further interim
analyses in alignment with stopping guidelines. All analyses will be undertaken using R. "

6a-i) Online questionnaires: describe if they were validated for online use and apply CHERRIES items to describe how the questionnaires were designed/deployed

If outcomes were obtained through online questionnaires, describe if they were validated for online use and apply CHERRIES items to describe how the questionnaires were designed/deployed [9].

	1	2	3	4	5	
subitem not at all important	○	○	○	○	○	essential

Does your paper address subitem 6a-i?

Copy and paste relevant sections from manuscript text

Your answer

6a-ii) Describe whether and how "use" (including intensity of use/dosage) was defined/measured/monitored

Describe whether and how "use" (including intensity of use/dosage) was defined/measured/monitored (logins, logfile analysis, etc.). Use/adoption metrics are important process outcomes that should be reported in any ehealth trial.

	1	2	3	4	5	
subitem not at all important	○	○	○	○	○	essential

Does your paper address subitem 6a-ii?

Copy and paste relevant sections from manuscript text

Your answer

6a-iii) Describe whether, how, and when qualitative feedback from participants was obtained

Describe whether, how, and when qualitative feedback from participants was obtained (e.g., through emails, feedback forms, interviews, focus groups).

	1	2	3	4	5	
subitem not at all important	○	○	○	○	○	essential

Does your paper address subitem 6a-iii?

Copy and paste relevant sections from manuscript text

Your answer

6b) Any changes to trial outcomes after the trial commenced, with reasons

Does your paper address CONSORT subitem 6b? *

Copy and paste relevant sections from the manuscript (include quotes in quotation marks "like this" to indicate direct quotes from your manuscript), or elaborate on this item by providing additional information not in the ms, or briefly explain why the item is not applicable/relevant for your study

There were no changes to the trial outcomes after the trial commenced.

7a) How sample size was determined

NPT: When applicable, details of whether and how the clustering by care provides or centers was addressed

7a-i) Describe whether and how expected attrition was taken into account when calculating the sample size

Describe whether and how expected attrition was taken into account when calculating the sample size.

	1	2	3	4	5	
subitem not at all important	○	○	○	○	○	essential

Does your paper address subitem 7a-i?

Copy and paste relevant sections from manuscript title (include quotes in quotation marks "like this" to indicate direct quotes from your manuscript), or elaborate on this item by providing additional information not in the ms, or briefly explain why the item is not applicable/relevant for your study

"A sample size of 150 participants will provide 80% power to detect a 25% increase of the target toothbrushing behaviour in the intervention group from 53% to 78% compared to the control group at three months, considering a 5% significance level and a 25% dropout rate. A cross-sectional study of a similar Australian population accessing public dental services found that 53% of children aged 3-8 brushed twice a day with toothpaste [24]. "

7b) When applicable, explanation of any interim analyses and stopping guidelines

Does your paper address CONSORT subitem 7b? *

Copy and paste relevant sections from the manuscript (include quotes in quotation marks "like this" to indicate direct quotes from your manuscript), or elaborate on this item by providing additional information not in the ms, or briefly explain why the item is not applicable/relevant for your study

" We did not conduct further interim analyses in alignment with stopping guidelines. All analyses will be undertaken using R. "

8a) Method used to generate the random allocation sequence

NPT: When applicable, how care providers were allocated to each trial group

Does your paper address CONSORT subitem 8a? *

Copy and paste relevant sections from the manuscript (include quotes in quotation marks "like this" to indicate direct quotes from your manuscript), or elaborate on this item by providing additional information not in the ms, or briefly explain why the item is not applicable/relevant for your study

"Randomisation was generated and accessed only by the study statistician using the Randomise R library of R statistical software (V.4.3.2) and is based on permuted block sizes (2,4,6) [19]. "

8b) Type of randomisation; details of any restriction (such as blocking and block size)

Does your paper address CONSORT subitem 8b? *

Copy and paste relevant sections from the manuscript (include quotes in quotation marks "like this" to indicate direct quotes from your manuscript), or elaborate on this item by providing additional information not in the ms, or briefly explain why the item is not applicable/relevant for your study

"Randomisation was generated and accessed only by the study statistician using the Randomise R library of R statistical software (V.4.3.2) and is based on permuted block sizes (2,4,6) [19]. "

9) Mechanism used to implement the random allocation sequence (such as sequentially numbered containers), describing any steps taken to conceal the sequence until interventions were assigned

Does your paper address CONSORT subitem 9? *

Copy and paste relevant sections from the manuscript (include quotes in quotation marks "like this" to indicate direct quotes from your manuscript), or elaborate on this item by providing additional information not in the ms, or briefly explain why the item is not applicable/relevant for your study

"Randomisation was generated and accessed only by the study statistician using the Randomise R library of R statistical software (V.4.3.2) and is based on permuted block sizes (2,4,6) [19]. "

10) Who generated the random allocation sequence, who enrolled participants, and who assigned participants to interventions

Does your paper address CONSORT subitem 10? *

Copy and paste relevant sections from the manuscript (include quotes in quotation marks "like this" to indicate direct quotes from your manuscript), or elaborate on this item by providing additional information not in the ms, or briefly explain why the item is not applicable/relevant for your study

"Clinicians recruited participants, parents self-enrolled in the study, and participants were assigned to the randomisation group via computer generation and notified via text message. All researchers were blinded as the TextCare™ system automatically implemented the text messaging program to the intervention arm. "

11a) If done, who was blinded after assignment to interventions (for example, participants, care providers, those assessing outcomes) and how

NPT: Whether or not administering co-interventions were blinded to group assignment

11a-i) Specify who was blinded, and who wasn't

Specify who was blinded, and who wasn't. Usually, in web-based trials it is not possible to blind the participants [1, 3] (this should be clearly acknowledged), but it may be possible to blind outcome assessors, those doing data analysis or those administering co-interventions (if any).

	1	2	3	4	5	
subitem not at all important	○	○	○	○	○	essential

Does your paper address subitem 11a-i? *

Copy and paste relevant sections from the manuscript (include quotes in quotation marks "like this" to indicate direct quotes from your manuscript), or elaborate on this item by providing additional information not in the ms, or briefly explain why the item is not applicable/relevant for your study

"Due to the nature of the intervention, participants were not blinded, but dental care providers and those analysing the data were blinded. "

11a-ii) Discuss e.g., whether participants knew which intervention was the "intervention of interest" and which one was the "comparator"

Informed consent procedures (4a-ii) can create biases and certain expectations - discuss e.g., whether participants knew which intervention was the "intervention of interest" and which one was the "comparator".

	1	2	3	4	5	
subitem not at all important	○	○	○	○	○	essential

Does your paper address subitem 11a-ii?

Copy and paste relevant sections from the manuscript (include quotes in quotation marks "like this" to indicate direct quotes from your manuscript), or elaborate on this item by providing additional information not in the ms, or briefly explain why the item is not applicable/relevant for your study

"Due to the nature of the intervention, participants were not blinded, but dental care providers and those analysing the data were blinded. "

11b) If relevant, description of the similarity of interventions

(this item is usually not relevant for ehealth trials as it refers to similarity of a placebo or sham intervention to a active medication/intervention)

Does your paper address CONSORT subitem 11b? *

Copy and paste relevant sections from the manuscript (include quotes in quotation marks "like this" to indicate direct quotes from your manuscript), or elaborate on this item by providing additional information not in the ms, or briefly explain why the item is not applicable/relevant for your study

This was not relevant for the trial as there was no placebo or sham intervention.

12a) Statistical methods used to compare groups for primary and secondary outcomes

NPT: When applicable, details of whether and how the clustering by care providers or centers was addressed

Does your paper address CONSORT subitem 12a? *

Copy and paste relevant sections from the manuscript (include quotes in quotation marks "like this" to indicate direct quotes from your manuscript), or elaborate on this item by providing additional information not in the ms, or briefly explain why the item is not applicable/relevant for your study

"Our statistical analysis followed an intention-to-treat principle and a pre-specified statistical analysis plan. To assess the effectiveness of the intervention, a logistic regression model, adjusting for whether parents are toothbrushing for their child twice a day with toothpaste at baseline and the stratification variables will be used, an adjusted Odds Ratio (aOR) with 95% confidence intervals (CI) will be reported. Similarly, all secondary behavioural outcomes will be analysed using the same logistic regression model. A statistical significance level of 0.05 will be used. Interaction between treatment and pre-specified covariates (planned dental procedure requiring GA vs. non-GA) and (tertiary vs. non-tertiary sites) was conducted to explore the heterogeneity of our results. No multiplicity adjustments will be performed for secondary outcomes, as they are exploratory by nature. If missingness is above 10% we will assess missingness at random for the primary outcome using relevant t-tests or chi-square tests for the relevant covariate. We did not conduct further interim analyses in alignment with stopping guidelines. All analyses will be undertaken using R. "

12a-i) Imputation techniques to deal with attrition / missing values

Imputation techniques to deal with attrition / missing values: Not all participants will use the intervention/comparator as intended and attrition is typically high in ehealth trials. Specify how participants who did not use the application or dropped out from the trial were treated in the statistical analysis (a complete case analysis is strongly discouraged, and simple imputation techniques such as LOCF may also be problematic [4]).

	1	2	3	4	5	
subitem not at all important	○	○	○	○	○	essential

Does your paper address subitem 12a-i? *

Copy and paste relevant sections from the manuscript (include quotes in quotation marks "like this" to indicate direct quotes from your manuscript), or elaborate on this item by providing additional information not in the ms, or briefly explain why the item is not applicable/relevant for your study

"If missingness is above 10% we will assess missingness at random for the primary outcome using relevant t-tests or chi-square tests for the relevant covariate. We did not conduct further interim analyses in alignment with stopping guidelines. All analyses will be undertaken using R. "

12b) Methods for additional analyses, such as subgroup analyses and adjusted analyses

Does your paper address CONSORT subitem 12b? *

Copy and paste relevant sections from the manuscript (include quotes in quotation marks "like this" to indicate direct quotes from your manuscript), or elaborate on this item by providing additional information not in the ms, or briefly explain why the item is not applicable/relevant for your study

"Our statistical analysis followed an intention-to-treat principle and a pre-specified statistical analysis plan. To assess the effectiveness of the intervention, a logistic regression model, adjusting for whether parents are toothbrushing for their child twice a day with toothpaste at baseline and the stratification variables will be used, an adjusted Odds Ratio (aOR) with 95% confidence intervals (CI) will be reported. Similarly, all secondary behavioural outcomes will be analysed using the same logistic regression model. A statistical significance level of 0.05 will be used. Interaction between treatment and pre-specified covariates (planned dental procedure requiring GA vs. non-GA) and (tertiary vs. non-tertiary sites) was conducted to explore the heterogeneity of our results. No multiplicity adjustments will be performed for secondary outcomes, as they are exploratory by nature. "

X26) REB/IRB Approval and Ethical Considerations [recommended as subheading under "Methods"] (not a CONSORT item)

X26-i) Comment on ethics committee approval

	1	2	3	4	5	
subitem not at all important	○	○	○	○	○	essential

Does your paper address subitem X26-i?

Copy and paste relevant sections from the manuscript (include quotes in quotation marks "like this" to indicate direct quotes from your manuscript), or elaborate on this item by providing additional information not in the ms, or briefly explain why the item is not applicable/relevant for your study

"This study was conducted in accordance with the principles of the Declaration of Helsinki. The protocol was reviewed and approved by the Western Sydney Local Health District Human Research Ethics Committee 2022/ETH01920. All participants took part voluntarily and provided written informed consent prior to enrolment. To ensure privacy and confidentiality, all collected data was deidentified. Additionally, the trial was prospectively registered with the Australia New Zealand Clinical Trials Registry (ACTRN12623000325606)."

x26-ii) Outline informed consent procedures

Outline informed consent procedures e.g., if consent was obtained offline or online (how? Checkbox, etc.?), and what information was provided (see 4a-ii). See [6] for some items to be included in informed consent documents.

	1	2	3	4	5	
subitem not at all important	○	○	○	○	○	essential

Does your paper address subitem X26-ii?

Copy and paste relevant sections from the manuscript (include quotes in quotation marks "like this" to indicate direct quotes from your manuscript), or elaborate on this item by providing additional information not in the ms, or briefly explain why the item is not applicable/relevant for your study

"All participants were given access to a participant Information and consent form via eConsent through the REDCap platform(Appendix X), prior to enrolment. "

X26-iii) Safety and security procedures

Safety and security procedures, incl. privacy considerations, and any steps taken to reduce the likelihood or detection of harm (e.g., education and training, availability of a hotline)

	1	2	3	4	5	
subitem not at all important	○	○	○	○	○	essential

Does your paper address subitem X26-iii?

Copy and paste relevant sections from the manuscript (include quotes in quotation marks "like this" to indicate direct quotes from your manuscript), or elaborate on this item by providing additional information not in the ms, or briefly explain why the item is not applicable/relevant for your study

"We did not conduct further interim analyses in alignment with stopping guidelines." More information about stopping guidelines is in our previously published protocol paper. https://journals.plos.org/plosone/article?id=10.1371/journal.pone.0310561

RESULTS

13a) For each group, the numbers of participants who were randomly assigned, received intended treatment, and were analysed for the primary outcome

NPT: The number of care providers or centers performing the intervention in each group and the number of patients treated by each care provider in each center

Does your paper address CONSORT subitem 13a? *

Copy and paste relevant sections from the manuscript (include quotes in quotation marks "like this" to indicate direct quotes from your manuscript), or elaborate on this item by providing additional information not in the ms, or briefly explain why the item is not applicable/relevant for your study

"Of the 154 participants who were recruited and randomised in this study between 24 August 2023 and 10 May 2024 (76 in the intervention group and 78 in the control group), 124 (80%) completed follow-up data collection of primary and secondary behavioural outcomes at the end of the 12-week intervention (Figure 1)."

13b) For each group, losses and exclusions after randomisation, together with reasons

Does your paper address CONSORT subitem 13b? (NOTE: Preferably, this is shown in a CONSORT flow diagram) *

Copy and paste relevant sections from the manuscript (include quotes in quotation marks "like this" to indicate direct quotes from your manuscript), or elaborate on this item by providing additional information not in the ms, or briefly explain why the item is not applicable/relevant for your study

Figure 1 is the CONSORT diagram that outlines this information.

13b-i) Attrition diagram

Strongly recommended: An attrition diagram (e.g., proportion of participants still logging in or using the intervention/comparator in each group plotted over time, similar to a survival curve) or other figures or tables demonstrating usage/dose/engagement.

	1	2	3	4	5	
subitem not at all important	○	○	○	○	○	essential

Does your paper address subitem 13b-i?

Copy and paste relevant sections from the manuscript or cite the figure number if applicable (include quotes in quotation marks "like this" to indicate direct quotes from your manuscript), or elaborate on this item by providing additional information not in the ms, or briefly explain why the item is not applicable/relevant for your study

Figure 1 is the CONSORT diagram that outlines this information.

14a) Dates defining the periods of recruitment and follow-up

Does your paper address CONSORT subitem 14a? *

Copy and paste relevant sections from the manuscript (include quotes in quotation marks "like this" to indicate direct quotes from your manuscript), or elaborate on this item by providing additional information not in the ms, or briefly explain why the item is not applicable/relevant for your study

"Of the 154 participants who were recruited and randomised in this study between 24 August 2023 and 10 May 2024 (76 in the intervention group and 78 in the control group), 124 (80%) completed follow-up data collection of primary and secondary behavioural outcomes at the end of the 12-week intervention (Figure 1)."

14a-i) Indicate if critical “secular events” fell into the study period

Indicate if critical “secular events” fell into the study period, e.g., significant changes in Internet resources available or “changes in computer hardware or Internet delivery resources”

	1	2	3	4	5	
subitem not at all important	○	○	○	○	○	essential

Does your paper address subitem 14a-i?

Copy and paste relevant sections from the manuscript (include quotes in quotation marks "like this" to indicate direct quotes from your manuscript), or elaborate on this item by providing additional information not in the ms, or briefly explain why the item is not applicable/relevant for your study

This was not relevant in our study as none of these events occurred.

14b) Why the trial ended or was stopped (early)

Does your paper address CONSORT subitem 14b? *

Copy and paste relevant sections from the manuscript (include quotes in quotation marks "like this" to indicate direct quotes from your manuscript), or elaborate on this item by providing additional information not in the ms, or briefly explain why the item is not applicable/relevant for your study

"Recruitment was completed once the anticipated sample size was reached."

15) A table showing baseline demographic and clinical characteristics for each group

NPT: When applicable, a description of care providers (case volume, qualification, expertise, etc.) and centers (volume) in each group

Does your paper address CONSORT subitem 15? *

Copy and paste relevant sections from the manuscript (include quotes in quotation marks "like this" to indicate direct quotes from your manuscript), or elaborate on this item by providing additional information not in the ms, or briefly explain why the item is not applicable/relevant for your study

This information is presented in Table 1 and 2.

15-i) Report demographics associated with digital divide issues

In ehealth trials it is particularly important to report demographics associated with digital divide issues, such as age, education, gender, social-economic status, computer/Internet/ehealth literacy of the participants, if known.

	1	2	3	4	5	
subitem not at all important	○	○	○	○	○	essential

Does your paper address subitem 15-i? *

Copy and paste relevant sections from the manuscript (include quotes in quotation marks "like this" to indicate direct quotes from your manuscript), or elaborate on this item by providing additional information not in the ms, or briefly explain why the item is not applicable/relevant for your study

This information is presented in Table 1.

16) For each group, number of participants (denominator) included in each analysis and whether the analysis was by original assigned groups

16-i) Report multiple “denominators” and provide definitions

Report multiple “denominators” and provide definitions: Report N’s (and effect sizes) “across a range of study participation [and use] thresholds” [1], e.g., N exposed, N consented, N used more than x times, N used more than y weeks, N participants “used” the intervention/comparator at specific pre-defined time points of interest (in absolute and relative numbers per group). Always clearly define “use” of the intervention.

	1	2	3	4	5	
subitem not at all important	○	○	○	○	○	essential

Does your paper address subitem 16-i? *

Copy and paste relevant sections from the manuscript (include quotes in quotation marks "like this" to indicate direct quotes from your manuscript), or elaborate on this item by providing additional information not in the ms, or briefly explain why the item is not applicable/relevant for your study

Additional outcomes of oral health self-efficacy were reported in a subset of 102 (68/102) in the intervention group and (64/102) in the control group participants. They were overall suggestive of a pattern of improved self-efficacy though few measures met statistical significance. A greater percentage of intervention participants reported improved knowledge about how to brush their child's teeth aOR, 5.47 [95% CI, 1.48-25.19]) and about how to maintain a healthy bedtime routine (aOR, 5.76 [95% CI, 1.38-32.16]) there was no difference in knowledge around providing health drink and snack options (aOR 2.7 [95% CI, 0.60-14.97]), and automatic Motivation around Oral health promoting bedtime routines (aOR 3.01 [95% CI, 00.96,10.54) (Table 4).

16-ii) Primary analysis should be intent-to-treat

Primary analysis should be intent-to-treat, secondary analyses could include comparing only "users", with the appropriate caveats that this is no longer a randomized sample (see 18-i).

	1	2	3	4	5	
subitem not at all important	○	○	○	○	○	essential

Does your paper address subitem 16-ii?

Copy and paste relevant sections from the manuscript (include quotes in quotation marks "like this" to indicate direct quotes from your manuscript), or elaborate on this item by providing additional information not in the ms, or briefly explain why the item is not applicable/relevant for your study

"Our statistical analysis followed an intention-to-treat principle and a pre-specified statistical analysis plan. "

17a) For each primary and secondary outcome, results for each group, and the estimated effect size and its precision (such as 95% confidence interval)

Does your paper address CONSORT subitem 17a? *

Copy and paste relevant sections from the manuscript (include quotes in quotation marks "like this" to indicate direct quotes from your manuscript), or elaborate on this item by providing additional information not in the ms, or briefly explain why the item is not applicable/relevant for your study

These results are reported in Table 3 and Table 4.

17a-i) Presentation of process outcomes such as metrics of use and intensity of use

In addition to primary/secondary (clinical) outcomes, the presentation of process outcomes such as metrics of use and intensity of use (dose, exposure) and their operational definitions is critical. This does not only refer to metrics of attrition (13-b) (often a binary variable), but also to more continuous exposure metrics such as "average session length". These must be accompanied by a technical description how a metric like a "session" is defined (e.g., timeout after idle time) [1] (report under item 6a).

	1	2	3	4	5	
subitem not at all important	○	○	○	○	○	essential

Does your paper address subitem 17a-i?

Copy and paste relevant sections from the manuscript (include quotes in quotation marks "like this" to indicate direct quotes from your manuscript), or elaborate on this item by providing additional information not in the ms, or briefly explain why the item is not applicable/relevant for your study

Process outcomes will be published in a future paper.

17b) For binary outcomes, presentation of both absolute and relative effect sizes is recommended

Does your paper address CONSORT subitem 17b? *

Copy and paste relevant sections from the manuscript (include quotes in quotation marks "like this" to indicate direct quotes from your manuscript), or elaborate on this item by providing additional information not in the ms, or briefly explain why the item is not applicable/relevant for your study

These results are reported in Table 3 and Table 4.

18) Results of any other analyses performed, including subgroup analyses and adjusted analyses, distinguishing pre-specified from exploratory

Does your paper address CONSORT subitem 18? *

Copy and paste relevant sections from the manuscript (include quotes in quotation marks "like this" to indicate direct quotes from your manuscript), or elaborate on this item by providing additional information not in the ms, or briefly explain why the item is not applicable/relevant for your study

"Oral Health Self-efficacy Outcomes
Additional outcomes of oral health self-efficacy were reported in a subset of 102 (68/102) in the intervention group and (64/102) in the control group participants. They were overall suggestive of a pattern of improved self-efficacy though few measures met statistical significance. A greater percentage of intervention participants reported improved knowledge about how to brush their child's teeth aOR, 5.47 [95% CI, 1.48-25.19]) and about how to maintain a healthy bedtime routine (aOR, 5.76 [95% CI, 1.38-32.16]) there was no difference in knowledge around providing health drink and snack options (aOR 2.7 [95% CI, 0.60-14.97]), and automatic Motivation around Oral health promoting bedtime routines (aOR 3.01 [95% CI, 00.96,10.54) (Table 4). "

18-i) Subgroup analysis of comparing only users

A subgroup analysis of comparing only users is not uncommon in ehealth trials, but if done, it must be stressed that this is a self-selected sample and no longer an unbiased sample from a randomized trial (see 16-iii).

	1	2	3	4	5	
subitem not at all important	○	○	○	○	○	essential

Does your paper address subitem 18-i?

Copy and paste relevant sections from the manuscript (include quotes in quotation marks "like this" to indicate direct quotes from your manuscript), or elaborate on this item by providing additional information not in the ms, or briefly explain why the item is not applicable/relevant for your study

a specific subgroup analysis was not explicitly conducted but this information could be extrapolated from the information we provide in Table 3 and 4.

19) All important harms or unintended effects in each group
(for specific guidance see CONSORT for harms)

Does your paper address CONSORT subitem 19? *

Copy and paste relevant sections from the manuscript (include quotes in quotation marks "like this" to indicate direct quotes from your manuscript), or elaborate on this item by providing additional information not in the ms, or briefly explain why the item is not applicable/relevant for your study

There were no reported harms related to the intervention. As mentioned in our published protocol, it is unlikely that there are harms associated with our intervention. Any relevant information is held in the participant consent form attached as an appendix item within our manuscript.

19-i) Include privacy breaches, technical problems

Include privacy breaches, technical problems. This does not only include physical “harm” to participants, but also incidents such as perceived or real privacy breaches [1], technical problems, and other unexpected/unintended incidents. “Unintended effects” also includes unintended positive effects [2].

	1	2	3	4	5	
subitem not at all important	○	○	○	○	○	essential

Does your paper address subitem 19-i?

Copy and paste relevant sections from the manuscript (include quotes in quotation marks "like this" to indicate direct quotes from your manuscript), or elaborate on this item by providing additional information not in the ms, or briefly explain why the item is not applicable/relevant for your study

This was not applicable to our study.

19-ii) Include qualitative feedback from participants or observations from staff/researchers

Include qualitative feedback from participants or observations from staff/researchers, if available, on strengths and shortcomings of the application, especially if they point to unintended/unexpected effects or uses. This includes (if available) reasons for why people did or did not use the application as intended by the developers.

	1	2	3	4	5	
subitem not at all important	○	○	○	○	○	essential

Does your paper address subitem 19-ii?

Copy and paste relevant sections from the manuscript (include quotes in quotation marks "like this" to indicate direct quotes from your manuscript), or elaborate on this item by providing additional information not in the ms, or briefly explain why the item is not applicable/relevant for your study

This will be addressed and published in a future study. "A process evaluation is now required to inform implementation at scale, including culturally appropriate adaptation of the programs and alignment with other family-focused preventive mHealth programs delivered in primary care settings [28]. Interoperability with electronic oral health records will enable health systems to prospectively and longitudinally measure this program’s long-term effects, and link this to long-term outcomes including clinical dental caries measures (dmft/DMFT), service utilisation, and hospital admission related to ECC [27]."

DISCUSSION

22) Interpretation consistent with results, balancing benefits and harms, and considering other relevant evidence

NPT: In addition, take into account the choice of the comparator, lack of or partial blinding, and unequal expertise of care providers or centers in each group

22-i) Restate study questions and summarize the answers suggested by the data, starting with primary outcomes and process outcomes (use)

Restate study questions and summarize the answers suggested by the data, starting with primary outcomes and process outcomes (use).

	1	2	3	4	5	
subitem not at all important	○	○	○	○	○	essential

Does your paper address subitem 22-i? *

Copy and paste relevant sections from the manuscript (include quotes in quotation marks "like this" to indicate direct quotes from your manuscript), or elaborate on this item by providing additional information not in the ms, or briefly explain why the item is not applicable/relevant for your study

"This RCT examined an SMS intervention program aimed at parents of children with dental caries. Most participants completed the SmilesUp program, and while the primary outcome was not significantly different, some secondary and additional outcomes were suggestive of positive behavioural changes and raised oral health literacy in intervention compared to control participants. These findings suggest such a program may have positive benefits if provided at scale to parents of children with caries.

In our sample, 18.6% of parents reported using health apps, but only 5.2% use oral health apps. This highlights both a gap and an opportunity to design and scale high-quality mHealth oral health programs that respond to growing demand for digital preventive care. We found no interaction effects among families whose children had planned dental procedure requiring general anaesthesia (GA) and those not requiring general anaesthesia and receiving care in community dental clinics. This suggests that the effect of the intervention is likely to be effective for a broad range of families. The interventions were also highly acceptable, with a 96% completion rate, indicating good feasibility for wider implementation. In the post COVID-19 context, digital oral health interventions have become increasingly acceptable to consumers in Australia and internationally [25].

Comparison With Prior Studies

This trial builds on existing evidence demonstrating that mHealth interventions can enhance parental knowledge and influence behaviours associated with ECC risk. Our findings are consistent with Ajay et al.'s 2023 systematic reviews of mHealth interventions targeting ECC risk factors, which showed improvements in oral health knowledge [14]. Our study extends this evidence by demonstrating effectiveness with behavioural outcomes, such as toothbrushing routines and bedtime oral health habits. The impact of the positive change in these behavioural outcomes are strongly connected to ECC clinical indicators, including Decayed Missing Filled Teeth (DMFT), caries incidence and the need for repeat invasive dental treatment [6].

Strengths

The strengths of this study include its randomised clinical trial design, its focus on a broad set of oral health promoting behavioural outcomes, implementation across a diverse participant population in metropolitan, regional, and rural NSW. Importantly, the intervention was co-designed with parents and pre-evaluated for acceptability and usefulness, which likely contributed to the high engagement and completion rates. By targeting multiple oral-health promoting behaviours, and evaluating the intervention alongside standard clinical care, demonstrates the added value of the intervention and highlights the importance of multi-level approaches to support sustained preventative oral health promoting behaviours [26].
"

22-ii) Highlight unanswered new questions, suggest future research

Highlight unanswered new questions, suggest future research.

	1	2	3	4	5	
subitem not at all important	○	○	○	○	○	essential

Does your paper address subitem 22-ii?

Copy and paste relevant sections from the manuscript (include quotes in quotation marks "like this" to indicate direct quotes from your manuscript), or elaborate on this item by providing additional information not in the ms, or briefly explain why the item is not applicable/relevant for your study

"Future Directions

Clinically, the results of the SmilesUp trial indicate that digital interventions can improve oral health behaviour and parental self-efficacy, which may translate into reduced ECC outcomes. As an mHealth intervention, it offers a less resource-intensive program delivered directly to families, supporting them in the home settings compared to traditional chairside advice. The limited effect on some behaviours, including sugar consumption, also highlights the need for multi-pronged and collaborative approaches to reducing common risk factors. From a policy perspective, these findings support integrating mHealth interventions into value-added clinical care models that emphasise preventative behaviour change in home settings as equally important to the clinical dental treatment the children receive [27].

A process evaluation is now required to inform implementation at scale, including culturally appropriate adaptation of the programs and alignment with other family-focused preventive mHealth programs delivered in primary care settings [28]. Interoperability with electronic oral health records will enable health systems to prospectively and longitudinally measure this program's long-term effects, and link this to long-term outcomes including clinical dental caries measures (dmft/DMFT), service utilisation, and hospital admission related to ECC [27]. Leveraging technologies, including artificial intelligence (AI) may further support tailored content delivery and continuous improvement within learning health systems [29]. Future research should examine the impact of scaling mHealth programs that leverage AI on the patient experience, sustained oral health behaviour change, long-term effectiveness and cost-effectiveness.
"

20) Trial limitations, addressing sources of potential bias, imprecision, and, if relevant, multiplicity of analyses

20-i) Typical limitations in ehealth trials

Typical limitations in ehealth trials: Participants in ehealth trials are rarely blinded. Ehealth trials often look at a multiplicity of outcomes, increasing risk for a Type I error. Discuss biases due to non-use of the intervention/usability issues, biases through informed consent procedures, unexpected events.

	1	2	3	4	5	
subitem not at all important	○	○	○	○	○	essential

Does your paper address subitem 20-i? *

Copy and paste relevant sections from the manuscript (include quotes in quotation marks "like this" to indicate direct quotes from your manuscript), or elaborate on this item by providing additional information not in the ms, or briefly explain why the item is not applicable/relevant for your study

"Limitations

This study has limitations, including: a follow-up rate of 82%, balanced between the intervention and control groups, likely due to the challenges of doing research among families with young children. The reliance on self-reported outcomes, which may be subject to social desirability bias, although this is balanced between the groups. The higher than expected rates of oral health compliance with the target behaviour in the study population (63% compared to the anticipated 53%), and the study being powered with an expectation of a large difference in efficacy between intervention and control of 25%. It is also important to note that trial participants may be a more highly motivated population than the broader population. Recruitment within public dental clinics introduces selection bias, however the inclusion of participants were from diverse settings in metro, regional and rural areas may also enhance representativeness. The high level of missingness could have influenced results; however, this was accounted for as participant characteristics were similar between groups (Appendix p.52-61). In fact, the high completion rate of participants previously not engaged in the target behaviour of tooth brushing suggests the intervention is particularly relevant for those most in need, highlighting potential benefits in health equity (Appendix p.58). The short follow-up period (12 weeks) limits conclusions regarding long-term behavioural maintenance and clinical outcomes although there is a strong indication that these sustained oral health behaviours will positively impact longer-term outcomes [27]. Future longitudinal studies should explore whether mHealth initiatives like SmilesUp can be effective in preventing longer-term clinical and service utilisation outcomes related to ECC.
"

21) Generalisability (external validity, applicability) of the trial findings

NPT: External validity of the trial findings according to the intervention, comparators, patients, and care providers or centers involved in the trial

21-i) Generalizability to other populations

Generalizability to other populations: In particular, discuss generalizability to a general Internet population, outside of a RCT setting, and general patient population, including applicability of the study results for other organizations

	1	2	3	4	5	
subitem not at all important	○	○	○	○	○	essential

Does your paper address subitem 21-i?

Copy and paste relevant sections from the manuscript (include quotes in quotation marks "like this" to indicate direct quotes from your manuscript), or elaborate on this item by providing additional information not in the ms, or briefly explain why the item is not applicable/relevant for your study

"Future longitudinal studies should explore whether mHealth initiatives like SmilesUp can be effective in preventing longer-term clinical and service utilisation outcomes related to ECC. Recruiting from a larger sample may also enhance the generalisability of these findings."

21-ii) Discuss if there were elements in the RCT that would be different in a routine application setting

Discuss if there were elements in the RCT that would be different in a routine application setting (e.g., prompts/reminders, more human involvement, training sessions or other co-interventions) and what impact the omission of these elements could have on use, adoption, or outcomes if the intervention is applied outside of a RCT setting.

	1	2	3	4	5	
subitem not at all important	○	○	○	○	○	essential

Does your paper address subitem 21-ii?

Copy and paste relevant sections from the manuscript (include quotes in quotation marks "like this" to indicate direct quotes from your manuscript), or elaborate on this item by providing additional information not in the ms, or briefly explain why the item is not applicable/relevant for your study

"Interoperability with electronic oral health records (eOHRs) will enable increased ease of opting into the program and will allow health systems to prospectively and longitudinally measure this program's long-term effects. This can include long-term outcomes available on eOHRs, including clinical dental caries measures (dmft/DMFT), service utilisation, and hospital admission related to ECC [27]. "

OTHER INFORMATION

23) Registration number and name of trial registry

Does your paper address CONSORT subitem 23? *

Copy and paste relevant sections from the manuscript (include quotes in quotation marks "like this" to indicate direct quotes from your manuscript), or elaborate on this item by providing additional information not in the ms, or briefly explain why the item is not applicable/relevant for your study

Trial Registration: Registration with the Australia New Zealand Clinical Trials Registry, ACTRN12623000325606.

24) Where the full trial protocol can be accessed, if available

Does your paper address CONSORT subitem 24? *

Cite a Multimedia Appendix, other reference, or copy and paste relevant sections from the manuscript (include quotes in quotation marks "like this" to indicate direct quotes from your manuscript), or elaborate on this item by providing additional information not in the ms, or briefly explain why the item is not applicable/relevant for your study

A full protocol has been previously published and available at:
https://journals.plos.org/plosone/article?id=10.1371/journal.pone.0310561
"A single-blinded parallel, randomised controlled trial (RCT) (1:1 allocation ratio). The protocol has been previously published [16], a concise summary of our methods is provided below. "

25) Sources of funding and other support (such as supply of drugs), role of funders

Does your paper address CONSORT subitem 25? *

Copy and paste relevant sections from the manuscript (include quotes in quotation marks "like this" to indicate direct quotes from your manuscript), or elaborate on this item by providing additional information not in the ms, or briefly explain why the item is not applicable/relevant for your study

"Funding

Western Sydney Local Health District. The funders did not have any roles in the study design, data collection and analysis, decision to publish, or preparation of the manuscript.
"

X27) Conflicts of Interest (not a CONSORT item)

X27-i) State the relation of the study team towards the system being evaluated

In addition to the usual declaration of interests (financial or otherwise), also state the relation of the study team towards the system being evaluated, i.e., state if the authors/evaluators are distinct from or identical with the developers/sponsors of the intervention.

	1	2	3	4	5	
subitem not at all important	○	○	○	○	○	essential

Does your paper address subitem X27-i?

Copy and paste relevant sections from the manuscript (include quotes in quotation marks "like this" to indicate direct quotes from your manuscript), or elaborate on this item by providing additional information not in the ms, or briefly explain why the item is not applicable/relevant for your study

No Conflicts of interest declared by authors.

About the CONSORT EHEALTH checklist

As a result of using this checklist, did you make changes in your manuscript? *

- ○ yes, major changes
- ◉ yes, minor changes
- ○ no

What were the most important changes you made as a result of using this checklist?

clarifying trial procedures.

How much time did you spend on going through the checklist INCLUDING making changes in your manuscript *

I spent four hours revising the document.

As a result of using this checklist, do you think your manuscript has improved? *

- (x) yes
- ( ) no
- ( ) Other:

Would you like to become involved in the CONSORT EHEALTH group?

This would involve for example becoming involved in participating in a workshop and writing an "Explanation and Elaboration" document

- ( ) yes
- (x) no
- ( ) Other:

Clear selection

Any other comments or questions on CONSORT EHEALTH

Your answer

STOP - Save this form as PDF before you click submit

To generate a record that you filled in this form, we recommend to generate a PDF of this page (on a Mac, simply select "print" and then select "print as PDF") before you submit it.

When you submit your (revised) paper to JMIR, please upload the PDF as supplementary file.

Don't worry if some text in the textboxes is cut off, as we still have the complete information in our database. Thank you!

Final step: Click submit !

Click submit so we have your answers in our database!

Submit Clear form

Never submit passwords through Google Forms.

This content is neither created nor endorsed by Google. - Contact form owner - Terms of Service - Privacy Policy

Does this form look suspicious? Report

Google Forms
