# Supplementary material for: Co-Designed Text Messaging Intervention for Parents of Children With Early Childhood Caries: Randomized Controlled Trial
Source: JMIR Mhealth Uhealth. 2026 Nov 3;14:e91720. doi: 10.2196/91720 (PMC13730512; doi:10.2196/91720)
Supplement: Supplementary file 4 [file mhealth-v14-e91720-s003.docx]

Supplement 4 Additional Analysis of missingness

We repeat tables 1, 2, and 3 by missingness of the primary outcome (N = 30).

| **Table 1. Baseline demographic characteristics of parents by missingness** | | | | |
| --- | --- | --- | --- | --- |
| **Characteristic** | **Missing** N = 30*^1^* | **Not missing** N = 124*^1^* | **Overall** N = 154*^1^* | **p-value***^2^* |
| **Parent's gender** |  |  |  | 0.077 |
| male | 10 / 30 (33.3%) | 30 / 124 (24.2%) | 40 / 154 (26.0%) |  |
| female | 19 / 30 (63.3%) | 94 / 124 (75.8%) | 113 / 154 (73.4%) |  |
| other (non-binary) | 0 / 30 (0.0%) | 0 / 124 (0.0%) | 0 / 154 (0.0%) |  |
| other (different term) | 0 / 30 (0.0%) | 0 / 124 (0.0%) | 0 / 154 (0.0%) |  |
| prefer not to answer | 1 / 30 (3.3%) | 0 / 124 (0.0%) | 1 / 154 (0.6%) |  |
| **Parent's age** |  |  |  | >0.9 |
| 18-20 | 0 / 30 (0.0%) | 1 / 124 (0.8%) | 1 / 154 (0.6%) |  |
| 20-25 | 0 / 30 (0.0%) | 5 / 124 (4.0%) | 5 / 154 (3.2%) |  |
| 26-35 | 12 / 30 (40.0%) | 44 / 124 (35.5%) | 56 / 154 (36.4%) |  |
| 36-45 | 15 / 30 (50.0%) | 63 / 124 (50.8%) | 78 / 154 (50.6%) |  |
| 46 or older | 2 / 30 (6.7%) | 8 / 124 (6.5%) | 10 / 154 (6.5%) |  |
| prefer not to answer | 1 / 30 (3.3%) | 3 / 124 (2.4%) | 4 / 154 (2.6%) |  |
| **Parent's level of education** |  |  |  | 0.7 |
| university degree | 14 / 30 (46.7%) | 59 / 124 (47.6%) | 73 / 154 (47.4%) |  |
| TAFE (or something similar) | 11 / 30 (36.7%) | 27 / 124 (21.8%) | 38 / 154 (24.7%) |  |
| year 12 | 2 / 30 (6.7%) | 14 / 124 (11.3%) | 16 / 154 (10.4%) |  |
| year 9-11 | 2 / 30 (6.7%) | 13 / 124 (10.5%) | 15 / 154 (9.7%) |  |
| year 8 or below | 0 / 30 (0.0%) | 4 / 124 (3.2%) | 4 / 154 (2.6%) |  |
| never attended school | 0 / 30 (0.0%) | 1 / 124 (0.8%) | 1 / 154 (0.6%) |  |
| prefer not to answer | 1 / 30 (3.3%) | 6 / 124 (4.8%) | 7 / 154 (4.5%) |  |
| **Country of birth** |  |  |  | 0.6 |
| Americas | 0 / 29 (0.0%) | 1 / 124 (0.8%) | 1 / 153 (0.7%) |  |
| North Africa and the Middle East | 5 / 29 (17.2%) | 11 / 124 (8.9%) | 16 / 153 (10.5%) |  |
| North-East Asia | 4 / 29 (13.8%) | 10 / 124 (8.1%) | 14 / 153 (9.2%) |  |
| North-West Europe | 1 / 29 (3.4%) | 2 / 124 (1.6%) | 3 / 153 (2.0%) |  |
| Oceania and Antarctica | 13 / 29 (44.8%) | 53 / 124 (42.7%) | 66 / 153 (43.1%) |  |
| Other codes | 0 / 29 (0.0%) | 1 / 124 (0.8%) | 1 / 153 (0.7%) |  |
| South-East Asia | 1 / 29 (3.4%) | 10 / 124 (8.1%) | 11 / 153 (7.2%) |  |
| Southern and Central Asia | 5 / 29 (17.2%) | 35 / 124 (28.2%) | 40 / 153 (26.1%) |  |
| Southern and Eastern Europe | 0 / 29 (0.0%) | 1 / 124 (0.8%) | 1 / 153 (0.7%) |  |
| (Missing) | 1 | 0 | 1 |  |
| **Do you use oral health apps?** | 1 / 30 (3.3%) | 7 / 124 (5.6%) | 8 / 154 (5.2%) | >0.9 |
| **Do you use other health apps?** | 6 / 30 (20.0%) | 23 / 124 (18.5%) | 29 / 154 (18.8%) | 0.9 |
| **Local health district where parent was enrolled in** |  |  |  | 0.2 |
| MurLHD | 0 / 30 (0.0%) | 3 / 124 (2.4%) | 3 / 154 (1.9%) |  |
| NBMLHD | 2 / 30 (6.7%) | 7 / 124 (5.6%) | 9 / 154 (5.8%) |  |
| NSLHD | 7 / 30 (23.3%) | 33 / 124 (26.6%) | 40 / 154 (26.0%) |  |
| SLHD-Sydney Dental Hospital | 8 / 30 (26.7%) | 16 / 124 (12.9%) | 24 / 154 (15.6%) |  |
| SouthLHD | 4 / 30 (13.3%) | 8 / 124 (6.5%) | 12 / 154 (7.8%) |  |
| WSLHD-WCOH | 9 / 30 (30.0%) | 57 / 124 (46.0%) | 66 / 154 (42.9%) |  |
| *^1^* n / N (%) | | | | |
| *^2^* Fisher’s exact test; Pearson’s Chi-squared test | | | | |
| **Table 2. Baseline demographic characteristics of child by missingness** | | | | |
| **Characteristic** | **Missing** N = 30*^1^* | **Not missing** N = 124*^1^* | **Overall** N = 154*^1^* | **p-value***^2^* |
| **child age** | 4.4 (1.8) | 4.2 (1.7) | 4.2 (1.7) | 0.4 |
| **Is your child receiving or have they received care with a general anaesthetic (GA)** | 11 / 30 (36.7%) | 38 / 124 (30.6%) | 49 / 154 (31.8%) | 0.5 |
| **Total number of children in your family** |  |  |  | 0.2 |
| 1 | 3 / 30 (10.0%) | 23 / 124 (18.5%) | 26 / 154 (16.9%) |  |
| 2 | 13 / 30 (43.3%) | 52 / 124 (41.9%) | 65 / 154 (42.2%) |  |
| 3 | 4 / 30 (13.3%) | 26 / 124 (21.0%) | 30 / 154 (19.5%) |  |
| 4 or more | 10 / 30 (33.3%) | 23 / 124 (18.5%) | 33 / 154 (21.4%) |  |
| **Are your other children younger than your child having the dental treatment?** | 10 / 30 (33.3%) | 42 / 124 (33.9%) | 52 / 154 (33.8%) | >0.9 |
| **Are your other children older than your child having the dental treatment?** | 14 / 30 (46.7%) | 50 / 124 (40.3%) | 64 / 154 (41.6%) | 0.5 |
| **I have children that are both older and younger** | 3 / 30 (10.0%) | 15 / 124 (12.1%) | 18 / 154 (11.7%) | >0.9 |
| **My other child is a twin to the current child** | 0 / 30 (0.0%) | 1 / 124 (0.8%) | 1 / 154 (0.6%) | >0.9 |
| **Not applicable to me (I have one child)** | 5 / 30 (16.7%) | 24 / 124 (19.4%) | 29 / 154 (18.8%) | 0.7 |
| **SEIFA (IRSAD quintile)** |  |  |  | 0.9 |
| 1 | 3 / 30 (10.0%) | 22 / 124 (17.7%) | 25 / 154 (16.2%) |  |
| 2 | 4 / 30 (13.3%) | 16 / 124 (12.9%) | 20 / 154 (13.0%) |  |
| 3 | 2 / 30 (6.7%) | 11 / 124 (8.9%) | 13 / 154 (8.4%) |  |
| 4 | 7 / 30 (23.3%) | 26 / 124 (21.0%) | 33 / 154 (21.4%) |  |
| 5 | 14 / 30 (46.7%) | 49 / 124 (39.5%) | 63 / 154 (40.9%) |  |
| *^1^* Mean (SD); n / N (%) | | | | |
| *^2^* Wilcoxon rank sum test; Pearson’s Chi-squared test; Fisher’s exact test | | | | |

| **Table 2. Baseline oral health behaviours by missingness** | | | | |
| --- | --- | --- | --- | --- |
| **Characteristic** | **Missing** N = 30*^1^* | **Not missing** N = 124*^1^* | **Overall** N = 154*^1^* | **p-value***^2^* |
| **How often do you brush your child's teeth with toothpaste? (baseline)** |  |  |  | 0.3 |
| Don’t know /unsure | 2 / 30 (6.7%) | 7 / 124 (5.6%) | 9 / 154 (5.8%) |  |
| Few times a week | 1 / 30 (3.3%) | 9 / 124 (7.3%) | 10 / 154 (6.5%) |  |
| Once a day | 5 / 30 (16.7%) | 38 / 124 (30.6%) | 43 / 154 (27.9%) |  |
| Twice a day | 22 / 30 (73.3%) | 66 / 124 (53.2%) | 88 / 154 (57.1%) |  |
| More than twice a day | 0 / 30 (0.0%) | 4 / 124 (3.2%) | 4 / 154 (2.6%) |  |
| **Met recommended toothbrushing requirements (baseline)** | 22 / 30 (73.3%) | 70 / 124 (56.5%) | 92 / 154 (59.7%) | 0.091 |
| **What type of toothpaste do you use for your child?** |  |  |  | 0.4 |
| Dont know /unsure | 1 / 30 (3.3%) | 10 / 124 (8.1%) | 11 / 154 (7.1%) |  |
| Non-fluoride toothpaste | 5 / 30 (16.7%) | 8 / 124 (6.5%) | 13 / 154 (8.4%) |  |
| Childrens fluoride toothpaste | 16 / 30 (53.3%) | 77 / 124 (62.1%) | 93 / 154 (60.4%) |  |
| Standard fluoride toothpaste | 8 / 30 (26.7%) | 28 / 124 (22.6%) | 36 / 154 (23.4%) |  |
| Prescribed toothpaste | 0 / 30 (0.0%) | 1 / 124 (0.8%) | 1 / 154 (0.6%) |  |
| **Met recommended Fluoride toothpaste usage (baseline)** | 24 / 30 (80.0%) | 106 / 124 (85.5%) | 130 / 154 (84.4%) | 0.6 |
| **How often does your child drink sugar sweetened drinks such as soft drinks, cordials, sports drinks, flavoured milk, fruit juices, energy drinks or iced teas?** |  |  |  | 0.5 |
| Dont know /unsure | 0 / 30 (0.0%) | 1 / 124 (0.8%) | 1 / 154 (0.6%) |  |
| Every day | 9 / 30 (30.0%) | 30 / 124 (24.2%) | 39 / 154 (25.3%) |  |
| A few times a week | 16 / 30 (53.3%) | 51 / 124 (41.1%) | 67 / 154 (43.5%) |  |
| Rarely | 5 / 30 (16.7%) | 39 / 124 (31.5%) | 44 / 154 (28.6%) |  |
| Never | 0 / 30 (0.0%) | 3 / 124 (2.4%) | 3 / 154 (1.9%) |  |
| **Met recommended sugar sweetened beverage consumption (baseline)** | 5 / 30 (16.7%) | 42 / 124 (33.9%) | 47 / 154 (30.5%) | 0.066 |
| **How often does your child go to bed with a sippy cup or bottle of milk?** |  |  |  | 0.6 |
| Dont know /unsure | 0 / 30 (0.0%) | 1 / 124 (0.8%) | 1 / 154 (0.6%) |  |
| Every day | 3 / 30 (10.0%) | 22 / 124 (17.7%) | 25 / 154 (16.2%) |  |
| A few times a week | 2 / 30 (6.7%) | 8 / 124 (6.5%) | 10 / 154 (6.5%) |  |
| Rarely | 2 / 30 (6.7%) | 17 / 124 (13.7%) | 19 / 154 (12.3%) |  |
| Never | 23 / 30 (76.7%) | 76 / 124 (61.3%) | 99 / 154 (64.3%) |  |
| **Met oral health promoting bedtime routines (baseline)** | 25 / 30 (83.3%) | 93 / 124 (75.0%) | 118 / 154 (76.6%) | 0.3 |
| **How often does your child eat sweet foods like chocolates, sweetened cereal, ice-cream, flavoured milk or yoghurt, biscuit, cakes, pastries, or snack/muesli bars?** |  |  |  | 0.7 |
| Dont know /unsure | 1 / 30 (3.3%) | 1 / 124 (0.8%) | 2 / 154 (1.3%) |  |
| Every day | 13 / 30 (43.3%) | 57 / 124 (46.0%) | 70 / 154 (45.5%) |  |
| A few times a week | 14 / 30 (46.7%) | 53 / 124 (42.7%) | 67 / 154 (43.5%) |  |
| Rarely | 2 / 30 (6.7%) | 12 / 124 (9.7%) | 14 / 154 (9.1%) |  |
| Never | 0 / 30 (0.0%) | 1 / 124 (0.8%) | 1 / 154 (0.6%) |  |
| **Met recommended sugar-sweetened food consumption (baseline)** | 2 / 30 (6.7%) | 13 / 124 (10.5%) | 15 / 154 (9.7%) | 0.7 |
| **How often does your child drink water? (Baseline)** |  |  |  | 0.4 |
| Dont know /unsure | 0 / 30 (0.0%) | 3 / 124 (2.4%) | 3 / 154 (1.9%) |  |
| Every day | 29 / 30 (96.7%) | 118 / 124 (95.2%) | 147 / 154 (95.5%) |  |
| A few times a week | 1 / 30 (3.3%) | 0 / 124 (0.0%) | 1 / 154 (0.6%) |  |
| Rarely | 0 / 30 (0.0%) | 2 / 124 (1.6%) | 2 / 154 (1.3%) |  |
| Never | 0 / 30 (0.0%) | 1 / 124 (0.8%) | 1 / 154 (0.6%) |  |
| **Met recommended water consumption by the child (baseline)** | 29 / 30 (96.7%) | 118 / 124 (95.2%) | 147 / 154 (95.5%) | >0.9 |
| *^1^* n / N (%) | | | | |
| *^2^* Fisher’s exact test; Pearson’s Chi-squared test | | | | |
